# Global ocean resistome revealed: Exploring antibiotic resistance gene abundance and distribution in TARA Oceans samples

**DOI:** 10.1093/gigascience/giaa046

**Published:** 2020-05-11

**Authors:** Rafael R C Cuadrat, Maria Sorokina, Bruno G Andrade, Tobias Goris, Alberto M R Dávila

**Affiliations:** 1 Department of Molecular Epidemiology, German Institute of Human Nutrition Potsdam-Rehbruecke - DIfE, Arthur-Scheunert-Allee 114–116, 14558 Nuthetal, Germany; 2 Institute for Inorganic and Analytical Chemistry, Friedrich-Schiller University, Lessingstrasse 8, 07743 Jena, Germany; 3 Animal Biotechnology Laboratory, Embrapa Southeast Livestock, EMBRAPA, Rodovia Washington Luiz, Km 234 s/n°, 13560-970 São Carlos, SP, Brazil; 4 Department of Molecular Toxicology, Research Group Intestinal Microbiology, German Institute of Human Nutrition Potsdam-Rehbruecke - DIfE, Arthur-Scheunert-Allee 114–116, 14558 Nuthetal, Germany; 5 Computational and Systems Biology Laboratory, Oswaldo Cruz Institute, FIOCRUZ, Av Brasil 4365, 21040-900 Rio de Janeiro, RJ, Brazil; 6 Graduate Program in Biodiversity and Health, Oswaldo Cruz Institute, FIOCRUZ, Av. Brasil 4365, 21040-900 Rio de Janeiro, RJ, Brazil

**Keywords:** β-lactamase, machine learning, marine metagenomics, colistin, tetracycline, multidrug resistance

## Abstract

**Background:**

The rise of antibiotic resistance (AR) in clinical settings is of great concern. Therefore, the understanding of AR mechanisms, evolution, and global distribution is a priority for patient survival. Despite all efforts in the elucidation of AR mechanisms in clinical strains, little is known about its prevalence and evolution in environmental microorganisms. We used 293 metagenomic samples from the TARA Oceans project to detect and quantify environmental antibiotic resistance genes (ARGs) using machine learning tools.

**Results:**

After manual curation of ARGs, their abundance and distribution in the global ocean are presented. Additionally, the potential of horizontal ARG transfer by plasmids and their correlation with environmental and geographical parameters is shown. A total of 99,205 environmental open reading frames (ORFs) were classified as 1 of 560 different ARGs conferring resistance to 26 antibiotic classes. We found 24,567 ORFs in putative plasmid sequences, suggesting the importance of mobile genetic elements in the dynamics of environmental ARG transmission. Moreover, 4,804 contigs with >=2 putative ARGs were found, including 2 plasmid-like contigs with 5 different ARGs, highlighting the potential presence of multi-resistant microorganisms in the natural ocean environment. Finally, we identified ARGs conferring resistance to some of the most relevant clinical antibiotics, revealing the presence of 15 ARGs similar to mobilized colistin resistance genes (*mcr*) with high abundance on polar biomes. Of these, 5 are assigned to *Psychrobacter*, a genus including opportunistic human pathogens.

**Conclusions:**

This study uncovers the diversity and abundance of ARGs in the global ocean metagenome. Our results are available on Zenodo in MySQL database dump format, and all the code used for the analyses, including a Jupyter notebook js avaliable on Github. We also developed a dashboard web application (http://www.resistomedb.com) for data visualization.

## Introduction

Antibiotic-resistant bacteria are a global public health issue and an economic burden to the entire world, especially in developing countries. Projections have shown that, if the emergence of multi-resistant bacteria continues at the same rate, they will cause 10 million deaths per year, which would outnumber cancer-related deaths [1, 2]. Despite its impact on human health, antibiotic resistance (AR) is a natural phenomenon and one of the most common bacterial defense mechanisms. For example, the resistance to β-lactam antibiotics, conferred by β-lactamase activity, is estimated to have emerged >1 billion years ago [3, 4]. Some authors argue that β-lactamase genes are part of inter- and intra-community communication and used in the defense repertoires of organisms sharing the same biological niche [5, 6].

The collection of antibiotic resistance genes (ARGs) in a given environment or organism is known as the resistome, and such genes have been detected in different natural environments, such as oceans [7], lakes [8], rivers [9], remote pristine Antarctic soils [10], and impacted Arctic tundra wetlands [11]. Studies also showed that anthropogenic activity (e.g., overuse of antibiotics and their subsequent release via wastewater into the environment) could lead to the spread of clinically relevant ARGs across natural environments [12, 13]. Therefore, the investigation of the natural context of ARGs, their geographic distribution, dynamics, and, in particular, their presence on horizontally transferable mobile genetic elements, such as plasmids, transposons, and phages, is crucial to assess their potential to emerge and spread [14–16]. Owing to modern advances in DNA sequencing and bioinformatics, it is now possible to study the presence and prevalence of ARGs in different environments. However, most of the published studies targeted only 1 or a few classes of ARGs and were limited to specific environments and geographic locations. The oceans cover ∼70% of Earth's surface, harbouring a significant diversity of planktonic microorganisms, forming a complex ecological network that is still understudied [17, 18]. To tackle this problem, the number of ocean metagenomic projects stored in public databases has been growing. Again, the lack of related metadata have made it challenging to conduct high-throughput gene screenings and correlations with environmental factors. Fortunately, the TARA Oceans project [19] measured several marine environmental conditions across the globe and stored them as structured metadata. This rich and unique dataset, together with the metagenome sequences [19], will allow the use of machine and deep learning approaches to search for gene and species distributions and their correlation to environmental parameters. In this study, we applied deepARG [20], a deep learning approach for ARG identification, to screen co-assembled TARA Oceans contigs [21]. After the manual curation of ARGs, we classified the results of the deepARG screening taxonomically. Furthermore, ARG abundance was quantified, and ordinary least squares (OLS) regression with association analyses between the quantification of ARGs and environmental parameters was used. We also explored the presence of ARGs located on putative plasmids to investigate the potential of these oceanic environments to act as a reservoir of potentially mobile ARGs.

## Methods

### Metagenomic data

A total of 12 co-assembled metagenomes from different oceanic regions explored by the TARA Oceans expedition, with contigs larger than 1 kb, were obtained from the dataset published in 2017 by Delmont et al. [22]. Raw reads of 243 samples (378 sequencing runs; accession numbers PRJEB1787, PRJEB6606, and PRJEB4419) were obtained from the European Bioinformatics Institute (EBI) European Nucleotide Archive (ENA) database [23].

Sample identifiers and metadata were obtained from the TARA Oceans companion website tables [24]. Samples were collected at different sites and depths and successively filtered using a single or a combination of membranes with pore sizes of 0.1, 0.2, 0.45, 0.8, 1.6, and 3 μm to retain different size fractions (i.e., viruses, giant viruses, and prokaryotes) [24]. We created a variable called “fraction,” where the upper and lower filtration membrane size were used together to define groups. However, owing to methodological limitations (described in the Results and Methods sections), viruses and giant viruses (giruses) enriched samples were excluded from quantitative analysis.

### Environmental ARG prediction

Open reading frame (ORF) prediction was performed on the 12 co-assembled metagenomes using MetaGeneMark v3.26 [25] with default parameters (sequences larger than 60 nucleotides). The screening for ARGs was performed with DeepARG [20] on the predicted ORFs using gene models. The deepARG tool was developed, taking into account a dissimilarity matrix using all ARG categories of 3 curated and merged databases (Antibiotic Resistance Genes Database [ARDB], Comprehensive Antibiotic Resistance Database [CARD], and UniProt) [20]. This approach is an alternative to the “best hits” of sequence searches against existing databases, which produces a high rate of false-negative results [20]. An ORF was classified as ARG if the estimated probability was ≥0.8. Contigs containing ≥1 putative ARG were analysed with PlasFlow 1.1 [26] using a probability threshold of 0.7 to check for a potential plasmidial location of ARGs. We also investigated the number and distribution of contigs with 2 or more putative ARGs to check for multiple resistance and/or whole ARG operons from environmental samples. Putative ARGs (and their respective contig) were submitted to Kaiju v1.6.2 [27] for taxonomic classification, with the option “run mode” set as “greedy.” Later, we conducted a manual curation of each ARG to check for misannotations and inconsistencies. BLASTp searches [28] were performed against the non-redundant (NR) protein database, with default parameters. Results with an e-value lower than e^−5^ were considered. Conserved domains (CDDs) and annotations in the source databases (ARDB [29], CARD [30], and UniProt [31]) were manually inspected. These results were used to classify misannotated/misclassified ARGs into different categories: (i) misannotated genes or gene families with low support for ARG prediction, i.e., all source database sequences exhibiting non-ARGs as top 5 BLASTp (against NR database) hits with an e-value cut-off of e^−5^. Included are especially cases with an unambiguously erroneous original annotation (examples are described in the Results). All of these misannotated ARGs were removed from our database and the downstream analyses; (ii) housekeeping genes that confer resistance only when specifically mutated; (iii) housekeeping genes conferring resistance when overexpressed; (iv) regulatory sequences responsible for ARG activation or overexpression of housekeeping genes leading to a resistance phenotype. The ARG family descriptions of the source databases (mainly those of the CARD database) were used (in addition to literature information) to classify ARGs into this scenario; (v) sequences with both similarities to ARGs and non-ARGs, belonging to the same superfamily and/or sharing domains. BLASTp and CDD analysis were used to classify ARGs into this scenario in cases where the TARA sequences show non-ARGs and no specific CDD domain for that ARG among the top 10 BLASTp hits.

### ARG quantification and statistical tests on metagenomic samples

Environmental ARGs identified were used as a reference for raw read mapping by BBMAP v37.90 (default parameters) [32] after manual curation. The coverage, in terms of read count per gene, and the abundance, in terms of fragments per kilobase per million mapped reads (FPKM), of each ARG was then calculated for each sample by BBMAP. The average genome size (AGS) and genome equivalents (GEs) were estimated by the software MicrobeCensus v1.0.7 (default parameters) [33] to calculate reads per kilobase per genome equivalents (RPKG) as described [33]. The RPKG of an ARG in a metagenome was calculated by (i) counting the number of reads mapped to the ARG, (ii) dividing (i) by the length of the ARG in kilobase pairs, and (iii) dividing the result of (ii) by the number of sequenced genome equivalents:

$$\begin{equation*}
\mathrm{RPKG} = \frac{{\mathrm{Mapped}\,\mathrm{reads}/\mathrm{Gene}\,\mathrm{Length}\,(\mathrm{kb})}}{{\mathrm{Genome}\,\mathrm{equivalents}}},
\end{equation*}$$

where

$$\begin{equation*}
\mathrm{GE} = \frac{{\mathrm{Library}\,\mathrm{size}\,(\mathrm{bp})}}{{\mathrm{AGS}\,(\mathrm{bp})}}
\end{equation*}$$

and library size is the total number of sequenced base pairs.

RPKG values for all ORFs classified as the same ARG were summed for each sample. Environmental features, such as sample depth, biogeographic biomes, ocean and sea regions, and fractions, were used for sample grouping and statistical tests. Pairwise Tukey HSD and multivariate linear regression using OLS models were conducted in Python 3.6 using the library “statsmodels.” The OLS was performed considering the following formula:

$$\begin{eqnarray*}
&& {\rm{AR}}{{\rm{G}}_{{\rm{RPKG}}}}\sim{\rm{fraction}} + {\rm{Latitude}} + {\rm{Longitude}} + {\rm{depth}}\nonumber \\
&& \quad + {\rm{temp}}\_{\rm{c}} + {\rm{NO}}_{ 2}{\rm{NO}}_{ 3} + {\rm{PO}}_{ 4} + {\rm{SI}} + {\rm{Mean}}\_{\rm{Oxygen}} \nonumber \\
&&\quad + {\rm{Mean}}\_{\rm{Salinity}} + {\rm{OG}}\_{\rm{Shannon}},
\end{eqnarray*}$$

where ARG_RPKG_ (the dependent variable) is the sum of RPKM of all ARGs in a given class, and all the dependent variables are the selected environmental features. A 2-way ANOVA analysis was conducted on the coefficients obtained from the OLS regression to infer the significance of a feature. A Python Jupyter notebook with the code and the results for all the exploratory and statistical analyses is provided on GitHub [34].

### Phylogenetic analysis of environmental ARGs

Phylogenetic analyses were performed on environmental nucleotide sequences identified as clinically relevant ARGs, such as mobilized colistin resistance (MCR)-related sequences, for which reference sequences were retrieved from public databases (e.g., NCBI and deepARGdb). Multiple protein sequence alignments and phylogenetic trees were generated using the standard pipeline of Phylogeny.fr [35]. In short, sequences were aligned using MUSCLE (default parameters) [36], conserved blocks extracted with gblocks (default parameters) [37], and phylogenetic trees generated with phyML [38], using Whelan and Goldman (WAG) matrix substitution model and approximate likelihood-ratio test (ALRT) statistical test.

### Database design and implementation

A manually curated MySQL database was created with the environmental ARGs described and all the subsequent analysis results. Data downloaded and processed as described above were parsed with Java 8 and stored in the database with Hibernate. The database model is also managed by Hibernate in Java. The code is available on GitHub [39]. The resulting database contains 5 main data tables: “orf”, “arg”, “sample”, “organism”, and “xref”, containing cross-references between the different data sources. The additional 5 connection tables map in an SQL engine-free way the correspondences between the items from different tables. We provide the SQL dump and the database schema at Zenodo [40].

### Dash web application for data exploration and visualization

We developed a Python dashboard web application where the user can explore the results through interactive graphics (plotted with the plotly library). The application includes a geographical scatterplot, where it is possible to visualize the abundance of each ARG (or antibiotic class) selected by the user across all the samples in a world map; a boxplot, where environmental features can be chosen to group the samples and compare their abundances; a barplot with taxonomic classification of the selected ARG (different taxonomic levels for the visualization can be chosen); and a scatterplot with marginal distribution plots and trend line (OLS), where the X-axis represents the selected ARG, and the Y-axis, the environmental variables selected by the user (e.g., oxygen concentration, salinity, temperature, depth). In addition, a table containing information for each ORF is displayed. The additional information includes ORF ID, contig ID, antibiotic class, deepARG probability value, plasmid classification by PlasFlow, taxonomic classification by Kaiju (on the deepest level), the abundance of additional ARG ORFs in the same contig, and the total of ARG ORFs in the contig. A link to download the multi-fasta file of the selected ARG is also provided. The application can be accessed at [41]. The code and data for the dash app can be accessed at [42].

### Pipeline and code availability

**Figure 1:**
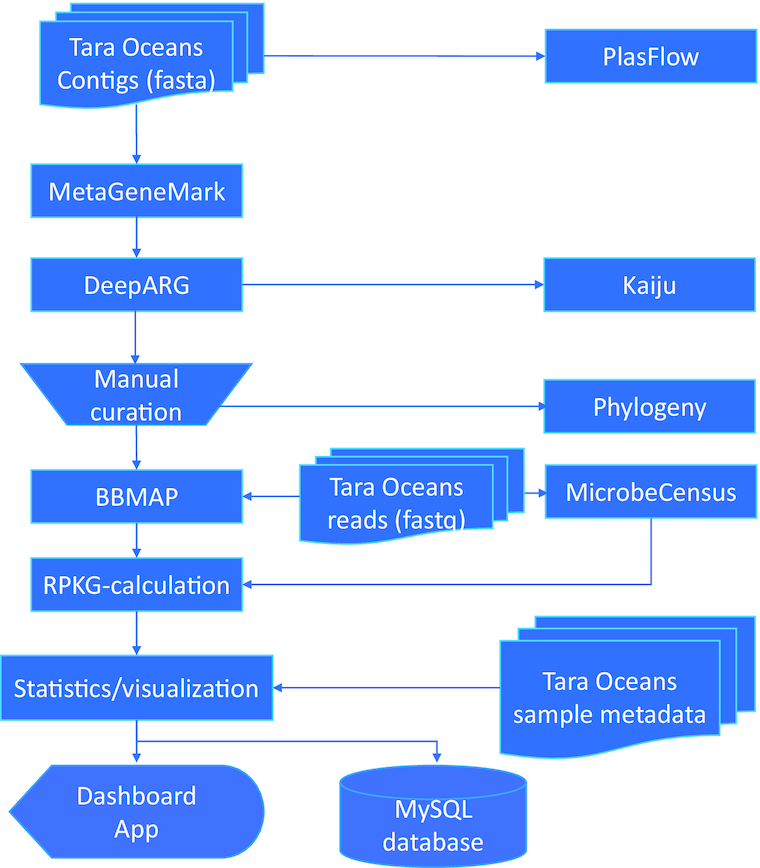
Flow chart used for ARG classification. The single steps and data used in the pipeline applied for the analyses presented in this work.

The code of the complete pipeline (Fig. 1) is in Bash and Python and is available at the project repository on GitHub [43].

## Results and Discussion

### Environmental ARG prediction and manual curation

A total of 41,249,791 ORFs were predicted from 15,600,278 assembled contigs by MetaGeneMark. These ORFs were used as input for ARG screening with the deepARG software [20], resulting in the classification of 116,425 TARA ORFs (0.28%) as putative ARGs, related to 594 clinically relevant ARGs that confer resistance to 28 antibiotic classes (classes defined in the deepARGdb). The number of contigs, ORFs, and putative ARGs from each oceanic region is available in Supplementary Table 1. It was necessary to conduct an extensive manual curation on the results owing to misannotations and misclassifications of ARGs in the databases used by deepARG. This curated dataset represents an important resource for further studies, including evolutionary and comparative studies.

A total of 34 ARGs were identified as misannotated or with low-quality annotation in the source database, leaving 560 ARGs for further analyses. A prominent example of a misannotated ARG is the *msr*B gene: while the *msr*B classified as ARG encodes an ABC-F subfamily protein leading to erythromycin and streptogramin B resistance, the corresponding fasta sequence in the CARD database [30] belongs to the *msrB* gene encoding methionine sulfoxide reductases B, not conferring AR. Another misannotated ARG is the *pat*A gene, an ABC transporter of *Streptococcus pneumoniae*, conferring resistance to fluoroquinolones, whose sequence is a putrescine aminotransferase (*pat*A) in the CARD database. A total of 99,205 ORFs identified as putative ARGs in categories (ii), (iii), (iv), and (v) (see Methods parts) were kept in the MySQL database for further studies, while they were not used in the quantification and statistical analyses. Category (ii) includes the identification of 10 families of housekeeping genes and the corresponding mutations that could infer resistance. Category (iii) included 9 ARGs whose overexpression can lead to resistance. For category (iv), we identified 41 regulatory sequences that have been identified as responsible for ARG expression or overexpression of housekeeping genes, causing the resistance phenotype. Category (v) included 187 putative ARGs that cannot be distinguished from non-ARGs by similarity alone (mostly due to commonly shared domains, e.g., ATPases). After the removal of those genes, a total of 13,163 ORFs (from the initial 116,425) classified as 313 ARGs were retained for quantification and further analysis (Supplementary Table 2).

**Figure 2:**
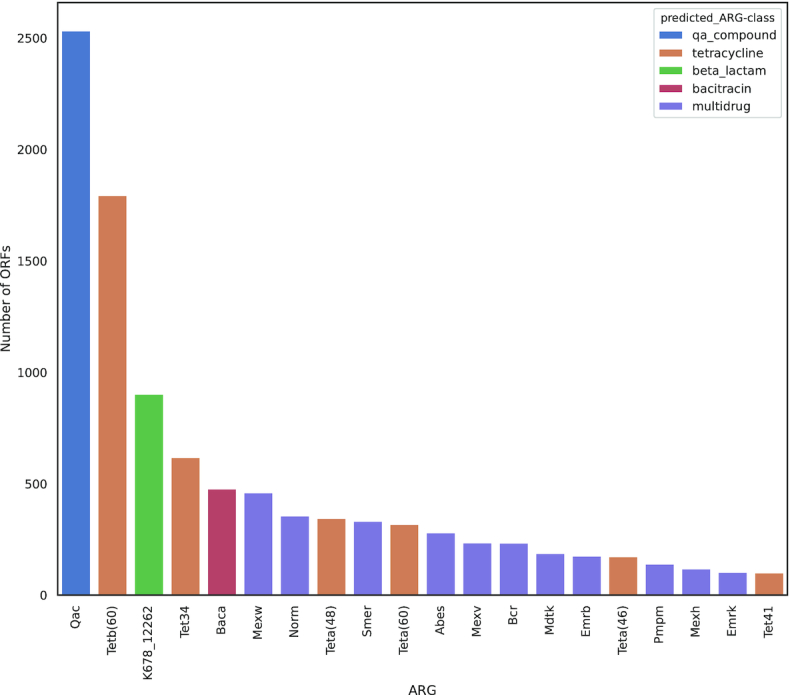
The 20 most frequent ARGs after manual curation (in number of ORFs on co-assembled contigs). The corresponding antibiotic resistance classes is depicted in the upper right.

The most frequent ARG (in number of ORFs) identified in the co-assembly dataset was Qac (multidrug efflux pumps named after their conferring resistance to quaternary ammonium compounds) with >2,500 overall occurrences, followed by TETB(60) (Fig. 2). The latter is an ABC transporter that confers resistance to tetracycline and tigecycline identified in a human saliva metagenomic library [44]. The ORFs conferring resistance to tetracycline combined are the most widespread, with several Tet and TetA classes accounting for ∼4,000 occurrences. Also, the most frequent ARG that confers resistance to β-lactams was identified as K678_12262, with ∼1,000 occurrences.

### Environmental ARGs in chromosomes and plasmids

We found a total of 24,567 putative ARGs (24.76% of the ORFs considered for the downstream analysis) present in contigs classified as plasmids by PlasFlow, which indicates the potential of horizontal genetic transfer (HGT). The occurrence of HGT of ARGs has already been described in clinical environments [45], wastewater treatment plants (activated sludge) [14, 46], and in fertilized soil [47], but little is known about ARG HGT in aquatic environments, especially in open ocean regions. As discussed in the section on *mcr* genes, it should be noted here that PlasFlow analyses bear a small chance (∼4%) of false-positive results as described [26], which especially could be the case with chromosomally integrated plasmids or very short contig sequence sizes.

### Multiple resistance presence in environmental contigs

The presence of 2 or more ARGs in a single contig was analysed to identify possible multi-resistant organisms. For this analysis, we only removed the ARGs from category (i) (misannotated sequences) because the presence of putative ARGs in the same contig and/or plasmid can give us additional functional evidence. We identified 4,063 contigs with multiple putative ARGs in contigs classified as chromosomes (up to 11 ARGs in the same contig), and 741 in contigs classified as a plasmid (up to 5 ARGs in the same contig), suggesting the presence of multi-resistant microorganisms in these environments (Table 1). We cannot exclude the possibility of multiple ARGs in both ends of plasmidial contigs being, in fact, artefacts, such as pieces of the same ARG in a circular contig. From the 4,192 contigs with 2 ARGs, 74 showed the same annotation for both ARGs (33 classified as plasmid). In Fig. S1, we show the distribution of the ARGs in the 2 putative plasmids containing 5 ARGs each.

**Table 1:** Distribution of multiple ARGs in chromosome and plasmids (classified by PlasFlow)

No. of ARGs	In chromosome	In plasmid
2	3,503	689
3	365	37
4	116	13
5	35	2
6	22	0
7	10	0
8	6	0
9	2	0
10	2	0
11	2	0

### Taxonomic classification of environmental ARGs

We classified 97,244 ARGs (98.02%) up to ≥1 taxonomic level using Kaiju [27]. Alphaproteobacteria (37,360 sequences) was identified as the largest taxonomic unit, followed by Gammaproteobacteria (19,355 sequences). A total of 124 ARGs were classified as of viral origin. The most frequent taxonomic viral groups identified were Prymnesiovirus (21 ARGs) and *Chrysochromulina ericina* virus (19 ARGs). However, all 124 viral ARGs were classified into category (v), and further investigations should be performed to confirm these findings. The presence of ARGs in phages and their potential HGT has been described in a Mediterranean river [48], pig faecal samples [15], fresh-cut vegetables, and agricultural soil [16].

In the contig containing 11 ARGs (TARA_ANW-k99_1343221), 9 were classified as HGW-Alphaproteobacteria-3 or HGW-Alphaproteobacteria-12, and as generic Alphaproteobacteria. The 2 residual ARGs were classified as belonging to *Parvibaculum lavamentivorans*, an alphaproteobacterial species first isolated from activated sludge in Germany [49]. A previous study showed the presence of ARGs in a strain of *Parvibaculum* from marine samples by functional metagenomics [7], which might indicate a broader ARG distribution among this clade. All ARGs from the other contig containing 11 ARGs (TARA_ANE-k99_4428305) were classified as *Micavibrio sp*., an obligate predatory bacterium exhibiting “vampire-like” behavior on gram-negative pathogens [50]. First isolated from wastewater samples, this genus has been considered as a potential new therapeutic approach against multi-resistant bacteria [51], including *mcr-1* positive strains [51], because no species from the genus *Micavibrio* was found to be pathogenic for humans [50]. However, if *Micavibrio* species are confirmed to contain 1 or multiple ARGs, this would raise concerns about any clinical therapeutic approaches with these bacteria. One of the putative plasmids containing 5 ARGs (contig TARA_PSE-k99_4996023, Supplementary Fig. S1) showed a taxonomic agreement between the classification of all its ARGs, which were assigned to the species *Tistrella mobilis*. Strains of this species were isolated from Thailand wastewater [52] and the Red Sea [53]. The other contig containing 5 ARGs of plasmidial origin was classified as *Halomonas desiderata*, a denitrifying bacterium first isolated from a municipal sewage treatment plant [54]. Two of the putative 5 ARGs in this contig were classified as DfrE and DfrA3, which confer resistance to trimethoprim. Previous work showed that another bacterial species of the same genus (*Halomonas marisflavi* type strain) is resistant to trimethoprim in vitro [55]. However, in the same study, *H. desiderata* did not show resistance to any of the antibiotics tested.

### ARG abundance and statistical tests on metagenomic samples

In previous sections, we aimed to find and characterize ARGs in metagenomic contigs obtained from co-assembled samples (by oceanic regions). In this section, we aim to quantify ARGs in individual samples, to understand their geographical distribution and the environmental features driving their abundance. The AGS of samples of fractions enriched for virus and girus showed biased and aberrant results for AGS (up to 395.4 Mb). These results are because AGS values (calculated by MicrobeCensus [33]) are inversely proportional to the number of reads mapping to housekeeping gene markers, and such genes have low abundance in virus-enriched samples. On the basis of this information, we kept only the 293 non–virus enriched sample runs for downstream quantitative analyses.

**Figure 3:**
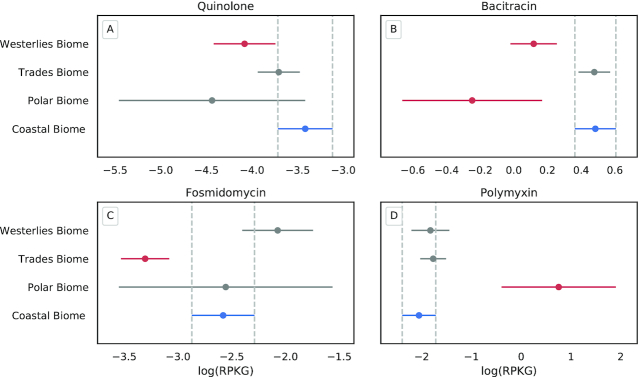
Significantly different mean abundances of ARG classes from oceanic biomes. Tukey HSD comparing the log-transformed RPKG of ARG classes for 4 biomes of TARA Oceans study. Shown are the means and 95% confidence intervals of RPKG for (A) quinolone ARGs, (B) bacitracin ARGs, (C) fosmidomycin ARGs, and (D) polymyxin ARGs. Blue indicates the reference for the test (coastal biome, chosen on the basis of its ecological relevance), and red, biome significantly different from the reference (*P*-value < 0.05).

For example, comparing biogeographical biomes, the quinolone and bacitracin ARG classes were significantly more abundant in the coastal biome than in the westerlies biome (adjusted Tukey HSD *P*-values 0.0476 and 0.0027, respectively). Furthermore, fosmidomycin ARGs were significantly (adjusted Tukey HSD *P*-value 0.0011) more abundant in the coastal biome than in the trades biome (Fig. 3, Supplementary Table 3). Quinolone ARGs were previously reported as highly abundant in Chinese coastal areas [56]. These results might indicate that quinolone, bacitracin, and fosmidomycin ARGs are under anthropogenic pressure in coastal environments, and future studies should be carried out to investigate this assumption in greater detail.

The pristine polar biome showed significantly higher RPKG values for polymyxin ARGs than any other biome. The antibiotics polymyxin B and E (also known as colistin) are the last resorts against gram-negative bacterial infections when modern antibiotics are ineffective, especially in cases of multiple drug-resistant *Pseudomonas aeruginosa* or carbapenemase-producing Enterobacteriaceae [57, 58]. We discuss mobilized colistin resistance genes (*mcr*) in greater detail below.

**Figure 4:**
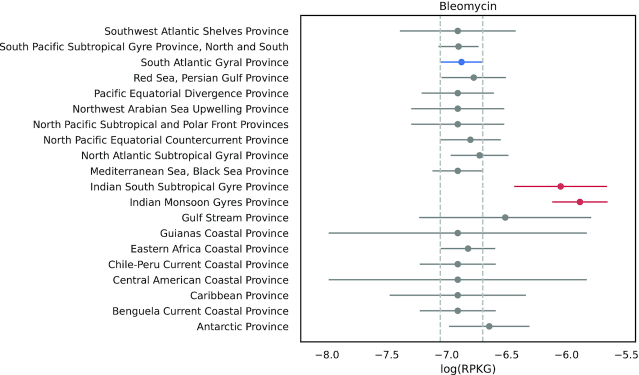
Bleomycin ARG abundance in marine provinces. Tukey HSD comparing the mean RPKG of ARGs from the class bleomycin. Blue indicates the reference for the test, and red, biome significantly different from the reference (*P*-value < 0.05). Error bars indicate 95% confidence intervals. The reference was chosen randomly.

When comparing the abundances of ARG classes in marine provinces, we found a significant difference (*P*-value < 0.05) of bleomycin class in 2 Indian provinces when compared to most of the other provinces (Fig.   4). Bleomycin resistance genes were previously reported to be in association with New Delhi metallo-β-lactamase (*ndm*-1) genes [59, 60]. In this study, *ndm*-like genes (classified by deepARG as *ndm*-17 variant) were also found in greater abundance in Indian South Subtropical Gyre province. The first variant of *ndm* was identified in a *Klebsiella pneumoniae* strain isolated from a Swedish patient who travelled to New Delhi, India [61]. Shortly after, it was spread globally in a few years and was also detected in other Enterobacteriaceae, which was a reason to classify NDMs as a potential worldwide public health problem [62].

In addition to the geographical location, we investigated the influence of other environmental parameters on the abundance of ARG classes. In our OLS models, the variables with significant *P*-values (<0.05 ANOVA test) for the largest number of antibiotic classes were “fraction” (14 classes), “sampling depth,” and “Shannon-Wiener index” (11 classes each). The fraction is a categorical variable, and the smallest size fraction (0.22–0.45 µm) was used as a reference for computing the coefficients in the model. This fraction is enriched for free-living, non-aggregating bacteria, which are smaller than other size fractions. For most classes (11 of 14), ≥1 category of fraction showed positive coefficients. For 3 of them, all fractions showed significantly more ARGs than the smallest fraction (tetracycline, aminoglycoside, and fosmidomycin). This result may indicate that free-living bacteria, in general, have a lower abundance of ARGs than particle-associated bacteria. These results corroborate a previous study, in which the antagonistic activity among pelagic marine bacteria (i.e., production of antibiotics) was more common in particle-associated bacteria than free-living bacteria [63].

For sampling depth, 5 of 11 classes were negatively correlated, indicating an increased abundance of ARGs in the deep water. For the Shannon-Wiener index, the only negative correlation was tetracycline, indicating an increased abundance of ARGs in samples with lower species richness.

The regression model for tetracycline presented the highest adjusted *R*^2^ (0.666) of all classes, with fraction, temperature, and sampling depth the most significant variables. For polymyxin, the adjusted *R*^2^ was the second highest (0.559), with temperature, Shannon index, and sampling depth the most significant variables.

In general, among the nutrients, nitrite+nitrate concentration (NO_2_NO_3_) was significant for the largest number of classes (7 classes), followed by inorganic phosphate (PO_4_)^3−^ (6 classes). Silicon (Si) was only significant for the classes fosmidomycin and tetracycline.

The role of inorganic nutrient concentration in ARG abundance is poorly understood and sometimes controversial. Some studies suggest that a high concentration of nutrients is negatively associated with ARGs because competitive interactions in nutrient-rich environments are less important [64]. However, the abundances of ARGs are increased in wastewater treatment plants [65] and agricultural soil receiving dairy manure [66], both environments rich in nutrients. Further studies should be conducted to better understand the role played by different nutrients in the abundance of ARGs of different classes in both pristine oligotrophic and impacted environments. Supplementary Table 4 reports all significant results of an ANOVA test on the coefficients of OLS for each class, and Supplementary Table 5 shows all the OLS results. A Q-Q plot of the OLS residuals is shown in Supplementary Fig. 2.

### Mobilized colistin resistance genes (mcr) and other polymyxin resistance genes

**Figure 5:**
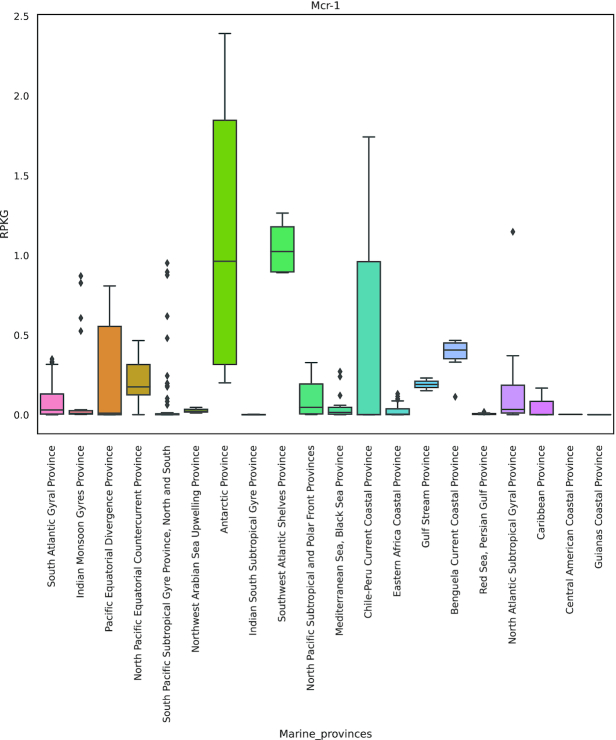
Mcr-1 distribution in TARA Oceans marine provinces. The boxplot shows the sum of RPKG values for all Mcr-1 ORFs.

**Figure 6:**
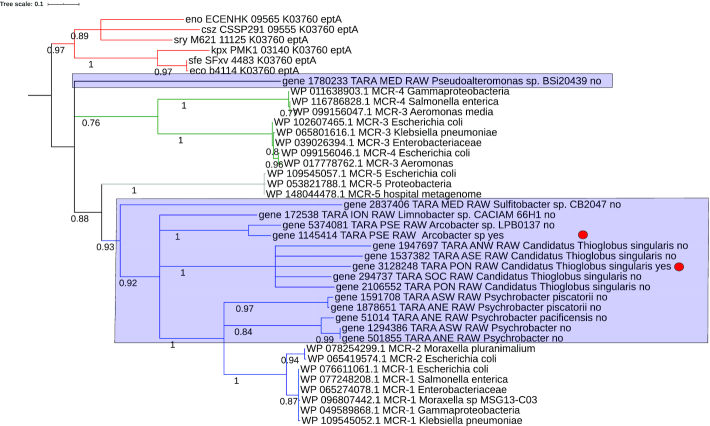
Phylogenetic tree of MCR sequences. The phylogenetic tree was inferred using the standard pipeline from phylogeny.fr (phyML with the “WAG” model and statistical test approximate likelihood-ratio [ALRT] for support values). Sequences for the outgroup *eptA* and clinical Mcr-1 to Mcr-5 were obtained from NCBI and used in addition to the sequences obtained from our results from TARA Oceans co-assemblies. The names of the TARA Oceans sequences displayed in the tree are defined with the ID of sequence, co-assembly ID, taxon name from Kaiju, and yes/no for plasmid classification from PlasFlow. The blue rectangles mark TARA sequences. The blue clade depicts the MCR-1/2 clade, the grey clade Mcr-5, the green clade MCR-3/4, and the red clade *eptA*. The red circles indicate sequences located on contigs classified as plasmids by PlasFlow. Numbers indicate ALRT support values.

Most mechanisms that confer resistance to colistin act against modifications of the lipid A moiety of lipopolysaccharide, with the addition of L-ara4N and/or phosphoethanolamine (PEA) to lipid A as the main mechanisms [67]. We found evidence for the occurrence of putative mobilized colistin resistance genes related to the recently discovered *mcr*-1 [68], which relies on the PEA addition to lipid A. The Mcr-1 enzyme was described as 41% and 40% identical to the PEA transferases LptA and EptC, respectively, and sequence comparisons suggest that the active-site residues are conserved. However, until the discovery of the plasmid-borne *mcr-1* in *Escherichia coli* from pig [68], colistin resistance has always been linked to chromosomally encoded genes with low or no possibility of horizontal transfer. Further studies showed a high prevalence of the *mcr-1* gene (e.g., 20% in animal-specific bacterial strains and 1% in human-specific bacterial strains in China), and the plasmid has been detected in several countries covering Europe, Asia, South America, North America, and Africa [69–76]. Further *mcr* variants have been described as *mcr*-1 to 9 as of December 2019 [77, 78]. In the present data, we detected 15 proteins classified as Mcr-1 by deepARG, most abundant in the Atlantic Southwest Shelves Province, followed by its adjacent region, Antarctic Province (Fig. 5). However, the version of deepARG that we used did not classify these sequences into the more recently described Mcr-2 to 9. Therefore, we performed a phylogenetic analysis (Fig. 6), which included sequences of different Mcrs (Mcr-1 to 5) and LptA (encoded by the gene *eptA*, used here as outgroup). The results suggested that 5 ORFs (from the genus *Psychrobacter*, family Moraxellaceae [79]) are close to the Mcr-1/2 clade with a support value of 1 (Fig. 6). Members of the genus *Psychrobacter* were isolated from a wide range of habitats, including food, clinical samples, skin, gills, and intestines of fish, seawater, and Antarctic sea ice [80–84]. Importantly, ≥2 isolates from this genus were already reported to be resistant to colistin (*Psychrobacter vallis sp. nov*. and *Psychrobacter aquaticus sp. nov*), both isolated from Antarctica [81]. Coincidently, the regions with greater RPKG mean values for Mcr-1 abundance in our study were Southwest Atlantic and Antarctic Province. Our results support that *Psychrobacter* might be an ecological reservoir for the transfer of PEA transferases to other pathogens, and further studies should be conducted to better elucidate the dynamics and evolution of ARGs in this genus. Also, some species of this genus were reported to cause opportunistic infections in humans, including ≥1 case reported to be associated with marine environment exposure [85]. In this context, it is therefore essential to increase monitoring by, e.g., including screenings specific for *mcr*-related genes in these genera.

The residual Mcr sequences, mostly belonging to the *Thioglobus* genus, were phylogenetically farther away from Mcr-1/2 and might constitute new, distinct Mcr variants (Fig. 6). Important to note is that the phylogenetically close relationship to Mcr sequences does not prove the function as a colistin-resistant gene, which awaits further experiments to confirm this role.

**Figure 7:**
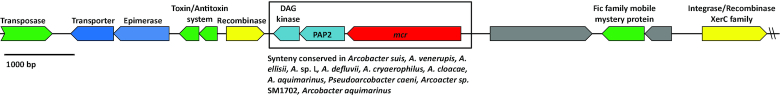
Genomic context of the *mcr* gene of contig TARA_PSE_k99_4834589. This contig was classified to be plasmidial by PlasFlow. Depicted are the first 13 ORFs from 28 of the whole contig, showing *mcr* (red) and surrounding genes and including the mobile element–related genes (green). DAG: diacylglycerol; PAP2: phosphatase PAP2 family protein; *mcr*: mobilized colistin resistance protein. Blue indicates other/metabolic genes; yellow, DNA-related genes; light blue, Mcr-accessory genes; and grey, hypothetical protein. Annotations from MetaGeneMark were manually refined using the conserved domains database and BLASTp against the SwissProt database. The taxonomy of *Arcobacter* species is stated as of December 2019 in the GenaBnk taxonomy database.

Only 2 *mcr* sequences were classified as present on plasmids via PlasFlow, which can be explained by the small size of many *mcr*-containing contigs (with 8 of them smaller than 3 kb). Additionally, a false-negative result from PlasFlow could be the result of a re-integration of plasmidial sequences into the chromosome—or that these *mcr* genes may constitute an ancestor of the plasmidial *E. coli mcr* sequences, as suggested for *mcr*-1 encoded by *Moraxella* species [79]. The 2 ARGs classified as located on a plasmid are detected in contigs with a size of 2 and 38 kb. The former, classified as belonging to a *Thioglobus* species, is challenging to validate as a plasmidial sequence owing to its small size. The latter is classified as a sequence of a *Poseidonibacter* species, a marine group of bacteria recently reclassified from the *Arcobacter* genus, the latter containing several pathogenic species [86]. A toxin-antitoxin system is encoded 2 ORFs upstream of the *mcr* gene, which might be an indication for a plasmidial location. However, no further genes that are usually located on *Arcobacter* spp. plasmids [87] were found on this contig, hampering its correct classification as a plasmidial *mcr*. That said, various mobile element genes located on this contig (Fig. 7) strengthen the assumption that this contig is related to a mobile genetic region. An unusual synteny of *mcr, pap*2, and a downstream encoded *dagK* was observed (Fig. 7), of which *dagK* only appears in *mcr-3* genetic environments [88]. Related genes (amino acid sequence identity of ∼70%) with a conserved gene synteny are found in several *Arcobacter* species (Fig. 7). A few *Arcobacter* species with a similar *mcr* gene were susceptible to colistin treatment [89], arguing against a colistin resistance conferred by this gene product. Further research is necessary to confirm or refute colistin resistance in marine *Poseidonibacter*.

The presence of *mcr*-related genes in both Antarctic and adjacent regions can also raise concerns about gene flow due to ice melting, a problem already discussed previously for other ARGs [90].

## Conclusions

This study uncovers the diversity and abundance of ARGs in the global ocean metagenome, conferring putative resistance to 26 classes of antibiotics. The extensive analysis leads to a detailed taxonomic classification and distribution of ARG abundance in different biomes. The present study also exposes the importance of monitoring coastal water for anthropogenic impact because the inflow of antibiotic-resistant strains by, e.g., wastewater might provide input of ARGs by HGT for environmental strains. This study could also affect investigations dealing with the evolutionary history of ARGs, with the herein-presented genes as ancestors of common ARGs in clinically relevant strains. Last but not least, the combination of multiple modern machine learning tools and other open source data science libraries such as Dash and Plotly produced a valuable resource for the scientific community working on further studies on ARGs in different environments.

## Availability of Source Code and Requirements

- Project name: ResistomeDB
- Project home page: https://resistomedb.com
- Operating system(s): Platform independent
- Programming language: Python
- Other requirements: None
- License: MIT
- RRID:SCR_018305

## Availability of Supporting Data and Materials

Snapshots of code and other supporting data are available in the *GigaScience* repository, GigaDB [91].

## Additional Files

**Figure S1:**ARG distribution in the 2 plasmids showing 5 ARGs each. The sizes of genes and distances are not scaled. PBP2B: methicillin-resistant PBP2; MTRA: transcriptional activator of the MtrCDE multidrug efflux pump; DFRE: dihydrofolate reductase; DFRA3: integron-encoded dihydrofolate reductase; BCR: bicyclomycin resistance protein; VANXA: variant of VANX D, D-dipeptidase; MEXH: membrane fusion protein of the efflux complex MexGHI-OpmD; VANSO: variant of VANS, required for high-level transcription of other van glycopeptide resistance genes; VANRI: regulatory transcriptional activator in the VanSR regulator within the VanI glycopeptide resistance gene cluster.

**Figure S2**: Q-Q plots for each ARG class. The figure shows the distribution of residuals from the OLS models for each ARG class.

**Table S1:** Number of contigs, ORFs, and putative ARGs for each oceanic region (metagenomic co-assembly).

**Table S2**: Manual curation of the ARGs. The table shows if the ARG was assigned for quantification studies and in each category was classified.

**Table S3**: Pairwise Tukey HSD significant results. The table shows the significant results (adjusted *P*-value > 0.05) of the paired Pairwise Tukey HSD for each pair of biogeographic biomes.

**Table S4**: ANOVA results for each ARG class. The table shows the significant results (*P*-value > 0.05) of the ANOVA for each ARG class.

**Table S5**: OLS results for each ARG class. The table shows the results of the OLS model for each ARG class, including model parameters and diagnostic

giaa046_GIGA-D-19-00446_Original_Submission

giaa046_GIGA-D-19-00446_Revision_1

giaa046_GIGA-D-19-00446_Revision_2

giaa046_Response_to_Reviewer_Comments_Original_Submission

giaa046_Response_to_Reviewer_Comments_Revision_1

giaa046_Reviewer_1_Report_Original_Submission Carlos Loucera MuÃ±ecas -- 1/28/2020 Reviewed

giaa046_Reviewer_2_Report_Original_Submission Kang Kang -- 2/13/2020 Reviewed

giaa046_Reviewer_2_Report_Revision_1 Kang Kang -- 3/27/2020 Reviewed

giaa046_Reviewer_2_Report_Revision_2 Kang Kang -- 4/3/2020 Reviewed

giaa046_Reviewer_3_Report_Original_Submission Felipe Hernandes -- 2/17/2020 Reviewed

giaa046_Reviewer_3_Report_Revision_1 Felipe Hernandes -- 3/20/2020 Reviewed

giaa046_Supplemental_Figures_and_Tables

## Abbreviations

AGS: average genome size; ALRT: approximate likelihood-ratio test; ANOVA: analysis of variance; AR: antibiotic resistance; ARDB: Antibiotic Resistance Genes Database; ARG: antibiotic resistance gene; BLAST: Basic Local Alignment Search Tool; bp: base pairs; CARD: Comprehensive Antibiotic Resistance Database; CDD: conserved domain; EBI: European Bioinformatics Institute; ENA: European Nucleotide Archive; FPKM: fragments per kilobase per million mapped reads; GE: genome equivalent; HGT: horizontal genetic transfer; HSD: honestly significant difference; kb: kilobase pairs; Mb: megabase pairs; MCR: mobilized colistin resistance; NCBI: National Center for Biotechnology Information; NDM: New Delhi metallo-β-lactamase; OLS: ordinary least squares; ORF: open reading frame; PEA: phosphoethanolamine; RPKG: reads per kilobase per genome equivalents; WAG: Whelan and Goldman.

## Competing Interests

The authors declare that they have no competing interests.

## Authors' Contributions

All authors conceived and designed the analysis; R.R.C.C., M.S., and B.G.A. performed the analysis; R.R.C.C. and M.S. conceived and designed the database; and R.R.C.C. designed the web application. All authors wrote the manuscript and revised it for significant intellectual content.
