# Supplementary material for: Global ocean resistome revealed: Exploring antibiotic resistance gene abundance and distribution in TARA Oceans samples
Source: Gigascience. 2020 May 11;9(5):giaa046. doi: 10.1093/gigascience/giaa046 (PMC7213576; doi:10.1093/gigascience/giaa046)
Supplement: giaa046_GIGA-D-19-00446_Revision_2 [file giaa046_giga-d-19-00446_revision_2.pdf]

GigaScience

Global ocean resistome revealed: exploring Antibiotic Resistance Genes (ARGs) abundance and distribution on TARA oceans samples

--Manuscript Draft--

**Manuscript Number:**	GIGA-D-19-00446R2
**Full Title:**	Global ocean resistome revealed: exploring Antibiotic Resistance Genes (ARGs) abundance and distribution on TARA oceans samples
**Article Type:**	Research
**Funding Information:**	
**Abstract:**	The rise of antibiotic resistance (AR) in clinical settings is one of the biggest modern global public health concerns. Therefore, the understanding of AR mechanisms, evolution, and global distribution is a priority due to its impact on the treatment course and patient survival. Besides all efforts in the elucidation of AR mechanisms in clinical strains, little is known about its prevalence and evolution in environmental microorganisms. In this study, 293 metagenomic samples from the TARA Oceans project were used to detect and quantify environmental antibiotic resistance genes (ARGs) using machine learning tools. After manual curation of ARGs, their abundance and distribution in the global ocean are presented, including taxonomical and phylogenetic classification. Additionally, the potential of horizontal ARG transfer by plasmids and their correlation with environmental and geographical parameters is shown. A total of 99,205 environmental open reading frames (ORFs) were classified as one of 560 different ARGs conferring resistance to 26 antibiotic classes. We found 24,567 ORFs in contigs classified as plasmid sequences, suggesting the importance of mobile genetic elements (MGEs) in the dynamics of environmental ARG transmission. Moreover, 4,804 contigs with more than two putative ARGs were found, including two plasmid-like contigs with five different ARGs, highlighting the potential presence of multi-resistant microorganisms in the natural ocean environment. Finally, we identified ARGs conferring resistance to some of the most relevant clinical antibiotics, revealing the presence of 15 ARGs similar to Mobilized Colistin Resistance genes (mcr) with high abundance on Polar Biomes. Of these, five are assigned to the genus Psychrobacter , a genus including opportunistic pathogens that can cause fatal infections in humans. Our results are available on Zenodo in MySQL database dump format, and all the code used for the analyses, including a Jupyter notebook, can be accessed on GitHub (https://github.com/rcuadrat/ocean_resistome). We also developed a dashboard web application (available at http://www.resistomedb.com) for data visualization.
**Corresponding Author:**	Alberto Davila<br><br>BRAZIL
**Corresponding Author Secondary Information:**	
**Corresponding Author's Institution:**	
**Corresponding Author's Secondary Institution:**	
**First Author:**	Rafael Ricardo de Castro Cuadrat
**First Author Secondary Information:**	
**Order of Authors:**	Rafael Ricardo de Castro Cuadrat
	Maria Sorokina
	Bruno Gabriel Andrade
	Tobias Goris
	Alberto Martin Rivera Davila
**Order of Authors Secondary Information:**	
**Response to Reviewers:**	Dear Editor,<br>we would like to thank you for the decision letter, and for the opportunity to resubmit a

revised copy of the manuscript.
We would also like to take this opportunity to express our thanks to the reviewers for the positive feedback and helpful comments for corrections and manuscript improvements. Please find below the point by point reply to reviewers comments.

Kind regards,

Related to the Previous Q1:
I can now access the server. But there seem some bugs, or things could be improved, eg.:
1) In the sequence viewer, when I want to check the sequence alignment and drag the position, it always returns to position 0;

Thanks for the comment, we noticed that in some browsers/versions this issue is happening. We are using a third-party tool for displaying the alignment and we are in touch with the developers to find a solution for this.

2) As a database, I saw a lot of data tables on the server. I wonder if users could download these tables as .csv or .tsv files;

The data tables used in the resistomedb.com app can be downloaded at the GitHub project page (https://github.com/rcuadrat/resistome_dash) under the folder data. We included the link for the data in the app. We add the link also in the manuscript "Dash web application for data exploration and visualisation section"

3) Also, it could be better if the data forms supporting the figures could be provided for users for personalized statistics or visualization;

We included the data in the GitHub of the main pipeline (https://github.com/rcuadrat/ocean_resistome).

3) The tables for genes annotated from contigs may miss some key information, such as alignment identity, alignment length, etc.

We did not include all the info due to lack of space for visualization. However, the full table can be downloaded from the data folder in the same GitHub repository mentioned in answers 2 (and now with the link in the app).

Related to the Previous Q2:
Regarding "a manual curation of each ARG to check for misannotations and inconsistencies" on alignment, P4 Line, the revised manuscript has largely improved, as standards now summarized into bullet points. However, I still feel a necessity to include clear details to dispel misunderstanding possibilities, for example: (i) "Sequences exhibiting non-ARGs as top 5 BLASTp hits", is it via blasting against NR database? Or other alignments? Is this the only criterium for point (i)?

Yes, blasting was against NR database and hits only were considered after reaching an e-value cutoff of e-5. We included these necessary details in the text.

(ii) "housekeeping genes that confer resistance only when specifically mutated", is there a clear list for these genes?
This list is compiled as supplementary table 2 (ten genes which belong tocategory ii). The information was manually compiled from the literature and CARD database.
Same issue for (iii) and (iv).
Also this information can be found in the supplementary table 2 (9 genes for category iii and 41 for category iv).

(v) please define "both similarities to ARGs and non-ARGs", by identity, e-value or what else?

We specified this to a lower extent already in the following line in the manuscript ("BLASTp and CDD analyses were used to classify ARGs into category (v) in cases where the TARA sequences show, non-ARGs and no specific CDD domain for that ARG

among the top 10 blastp hits").
We did not use specific cutoffs (of course only the most recommended e-5 used in general for blast searches). This was a manual curation with the individual decision taken non-automatically by the curator. For most of the cases, we hypothesize that using phylogenetic approaches would be more suitable for classifying those genes, so we keep them for future works.

Also, I disagree with the use of "top n blastp hits", it is not statistical. Different genes may have different numbers of homologous genes from different species, which makes this criterium unfair for all candidate genes.

We agree with the reviewer, this is why we chose a manual curation based on expert review and database searches. Each gene was analysed individually by a curator (the authors), based on best database hits, but also taking similarity, alignment coverage, e-value and literature research. For example, a potential ARG was not removed when the top 5 hits revealed 4 ARGs with good alignment stats, but with a 5th non-ARG showing a worse alignment. Since such a manual curation is limited several cutoffs were used to limit the amount of genes to analyze manually . Our ongoing work involves developing a full database paper, in which the community is invited to help curating these and more potential ARGs from further databases in the future.

Related to the Previous Q3:
I suggest the authors quote some references to say, "these data are clean data", or put some basic QC statistics.

For fastq submissions to EBI, it is mandatory to remove artificial sequences (primer, adaptors) as stated in https://ena-docs.readthedocs.io/en/latest/submit/fileprep/reads.html. This is the most crucial step on QC of Illumina reads, as the bbmap and other aligners are not too much sensitive to low-quality bases when doing mapping for quantification (biorxiv.org/content/10.1101/833962v1). In this study, we did not use the raw reads for assembly, but rather mapped reads to the previous published assembled dataset, so we found no necessity to show QC of the reads that passed the EBI check control.

Related to the Previous Q7c:
Many parameters are now available, especially for the Methods section "Environmental ARG prediction". But I am not sure regarding ARG annotation, "BLASTp with default parameter" should be accepted.
Normally ARGs should have an Identity >70, >90 or even >95, depending on the sample types and clinical relevance. To my point of view, identity (or also E-value) should be used for a basic filtration.

We added the general cutoff we used to accept a blast hit (e-5). Identity cutoffs are hard to implement due to the different evolutionary rates of the very different gene families analysed.

Parameter missing problem still partly remains in the Methods section "Phylogenetic analysis of environmental ARGs" and "ARGs quantification and statistical tests on metagenomic samples".

We added the statement ("default parameters") for the tools used because we did not modify any parameters. Together with the given version this should be consistent and make replication possible. The used phylogenetic tools are standard in the analysis pipeline Phylogenetic.fr. For the bbmap and microbecensus, we also added the "default parameters" in the text, and we reinforce the fact that the scripts used for those analyses are available on the GitHub repository, making it fully reproducible.

Related to Previous Q10:
Statistics is better than the description in the text. Also, regarding Table 1, I am curious how many of these contig-sharing ARGs are the same ARG, or from the same ARG family. If the authors could annotate the phage-like sequences or mobile elements, the

Powered by Editorial Manager® and ProduXion Manager® from Aries Systems Corporation

	story could be improved.<br><br>From the 4192 ones with 2 ARGs, only 74 shows the same annotation for both ARGs (33 classified as a plasmid). We added this information in the text.<br><br>Related to Previous Q18:<br>As I can see, gene names in the Figures remain unchanged. Eg. should be tetB, ermB, not TETB, ERMB.<br><br>Thank you for pointing this out. We fixed the figures with the right gene notations.
**Additional Information:**	Rafael Cuadrat and Alberto Davila
**Question**	**Response**
Are you submitting this manuscript to a special series or article collection?	No
**Experimental design and statistics**<br><br>Full details of the experimental design and statistical methods used should be given in the Methods section, as detailed in our Minimum Standards Reporting Checklist. Information essential to interpreting the data presented should be made available in the figure legends.<br><br>Have you included all the information requested in your manuscript?	Yes
**Resources**<br><br>A description of all resources used, including antibodies, cell lines, animals and software tools, with enough information to allow them to be uniquely identified, should be included in the Methods section. Authors are strongly encouraged to cite Research Resource Identifiers (RRIDs) for antibodies, model organisms and tools, where possible.<br><br>Have you included the information requested as detailed in our Minimum Standards Reporting Checklist?	Yes

Powered by Editorial Manager® and ProduXion Manager® from Aries Systems Corporation

Availability of data and materials<br><br>All datasets and code on which the conclusions of the paper rely must be either included in your submission or deposited in publicly available repositories (where available and ethically appropriate), referencing such data using a unique identifier in the references and in the "Availability of Data and Materials" section of your manuscript.<br><br>Have you have met the above requirement as detailed in our Minimum Standards Reporting Checklist?	Yes

Powered by Editorial Manager® and ProduXion Manager® from Aries Systems Corporation

Manuscript w. DOI
Click here to access/download;Manuscript;MS_04_02 with DOI.docx

Global ocean resistome revealed: exploring Antibiotic Resistance Genes (ARGs) abundance and distribution in TARA oceans samples

Rafael R. C. Cuadrat[1], Maria Sorokina[2], Bruno G. Andrade[3], Tobias Goris[4], Alberto M. R. Dávila[5*]

1 - Department of Molecular Epidemiology, German Institute of Human Nutrition Potsdam-Rehbruecke - DIfE, Arthur-Scheunert-Allee 114-116, 14558 Nuthetal, Germany, ORCID:0000-0001-8289-2599
2 - Friedrich-Schiller University, Lessingstrasse 8, 07743 Jena, Germany, ORCID: 0000-0001-9359-7149
3 - Animal Biotechnology Laboratory, Embrapa Southeast Livestock, EMBRAPA, Rodovia Washington Luiz, Km 234 s/nº, 13560-970 São Carlos, SP, Brazil.
4 - Department of Molecular Toxicology, Research Group Intestinal Microbiology, German Institute of Human Nutrition Potsdam-Rehbruecke - DIfE, Arthur-Scheunert-Allee 114-116, 14558 Nuthetal, Germany
5 - Computational and Systems Biology Laboratory, Oswaldo Cruz Institute, FIOCRUZ. Av Brasil 4365, 21040-900 Rio de Janeiro, RJ, Brazil.

*Corresponding author

Abstract

The rise of antibiotic resistance (AR) in clinical settings is one of the biggest modern global public health concerns. Therefore, the understanding of AR mechanisms, evolution, and global distribution is a priority due to its impact on the treatment course and patient survival. Besides all efforts in the elucidation of AR mechanisms in clinical strains, little is known about its prevalence and evolution in environmental microorganisms. In this study, 293 metagenomic samples from the TARA Oceans project were used to detect and quantify environmental antibiotic resistance genes (ARGs) using machine learning tools. After manual curation of ARGs, their abundance and distribution in the global ocean are presented, including taxonomical and phylogenetic classification.
Additionally, the potential of horizontal ARG transfer by plasmids and their correlation with environmental and geographical parameters is shown. A total of 99,205 environmental open reading frames (ORFs) were classified as one of 560 different ARGs conferring resistance to 26 antibiotic classes. We found 24,567 ORFs in contigs classified as plasmid sequences, suggesting the importance of mobile genetic

elements (MGEs) in the dynamics of environmental ARG transmission. Moreover, 4,804 contigs with more than two putative ARGs were found, including two plasmid-like contigs with five different ARGs, highlighting the potential presence of multi-resistant microorganisms in the natural ocean environment. Finally, we identified ARGs conferring resistance to some of the most relevant clinical antibiotics, revealing the presence of 15 ARGs similar to Mobilized Colistin Resistance genes (*mcr*) with high abundance on Polar Biomes. Of these, five are assigned to the genus *Psychrobacter*, a genus including opportunistic pathogens that can cause fatal infections in humans. Our results are available on Zenodo in MySQL database dump format, and all the code used for the analyses, including a Jupyter notebook, can be accessed on GitHub (https://github.com/rcuadrat/ocean_resistome). We also developed a dashboard web application (available at http://www.resistomedb.com) for data visualization.

Keywords: Beta-lactamase, machine learning, marine metagenomics, colistin, tetracycline , multidrug resistance

Introduction

Antibiotic-resistant bacteria are a global public health issue and an economic burden to the entire world, especially in developing countries. Projections showed that, if the emergence of multi-resistant bacteria continues at the same rate, they will cause 10 million deaths per year, which would outnumber cancer-related deaths [1,2]. Despite its impact on human health, antibiotic resistance (AR) is a natural phenomenon and one of the most common bacterial defense mechanisms. For example, the resistance to β-lactam antibiotics, conferred by beta-lactamase activity, is estimated to have emerged more than 1 billion years ago [3,4]. Some authors argue that beta-lactamase genes are part of inter- and intra-community communication and used in the defense repertoires of organisms sharing the same biological niche [5,6].

The collection of antibiotic resistance genes (ARGs) in a given environment or organism is known as the resistome, and such genes have been detected in different natural environments, such as oceans [7], lakes [8], rivers [9], remote pristine Antarctic soils [10] and impacted Arctic tundra wetlands [11]. Studies also showed that anthropogenic activity (*e.g.*, over-usage of antibiotics and their subsequent release via wastewater into the environment) could lead to the spread of clinically relevant ARGs across natural environments [12,13]. Therefore, the investigation of the natural context of ARGs, their geographic distribution, dynamics and, in particular, their presence on horizontally transferable mobile genetic elements (MGEs), such as plasmids, transposons, and phages, is crucial to assess their potential to emerge and spread [14–16]. Due to modern advances in DNA sequencing and bioinformatics, it is now possible to study the presence and prevalence of ARGs in different environments. However, most of the published studies targeted only one or a few classes of ARGs

and were limited to specific environments and geographic locations. The oceans cover around 70% of Earth's surface, harbouring a significant diversity of planktonic microorganisms, forming a complex ecological network that is still under-studied [17,18]. To tackle this problem, the number of ocean metagenomic projects stored in public databases has been growing. Again, the lack of related metadata made it challenging to conduct high-throughput gene screenings and correlations with environmental factors. Fortunately, the TARA oceans project [19] measured several marine environmental conditions across the globe and stored them as structured metadata. This rich and unique dataset, together with the metagenome sequences [19], will allow the use of machine and deep learning approaches to search for gene and species distribution and their correlation to environmental parameters. In this study, we applied deepARG [20], a deep learning approach for ARG identification, to screen co-assembled TARA oceans contigs [21]. After the manual curation of ARGs, we classified the results of the deepARG screening taxonomically. Furthermore, ARG abundance was quantified, and Ordinary Least Squares (OLS) regression with association analyses between the quantification of ARGs and environmental parameters was used. We also explored the presence of ARGs located on putative plasmids to investigate the potential of these oceanic environments to act as a reservoir of potentially mobile ARGs.

Methods

Metagenomic data

A total of 12 co-assembled metagenomes from different oceanic regions explored by the Tara Oceans expedition, with contigs larger than 1 kilobase were obtained from the dataset published in 2017 by Delmont et al. [22]. Raw reads of 243 samples (378 sequencing runs; accession numbers PRJEB1787, PRJEB6606, and PRJEB4419) were obtained from the EBI ENA database (https://www.ebi.ac.uk/ena).
Sample identifiers and metadata were obtained from the TARA oceans companion website tables [23]. Samples were collected at different sites and depths and successively filtered using a single, or a combination, of membranes with pore sizes of 0.1 μm, 0.2 μm, 0.45 μm, 0.8 μm, 1.6 μm, and 3 μm to retain different size fractions (i.e., viruses, giant viruses, and prokaryotes) [23]. We created a variable called fraction, where the upper and lower filtration membrane size were used together to define groups. However, due to methodological limitations (described in the results and methods section), viruses and giant viruses (giruses) enriched samples were excluded from quantitative analysis.

Environmental ARG prediction

Open reading frame (ORF) prediction was performed on the 12 co-assembled metagenomes using MetaGeneMark v3.26 [24] with default parameters (sequences larger than 60 nt). The screening for ARGs was performed with DeepARG [20] on the predicted ORFs using gene models. The deepARG tool was developed, taking into account a dissimilarity matrix using all ARG categories of three curated and merged databases (ARDB, CARD, and UNIPROT) [20]. This approach is an alternative to the "best hits" of sequence searches against existing databases, which produces a high rate of false negatives [20]. An ORF was classified as ARG if the estimated probability was equal to or greater than 0.8. Contigs containing at least one putative ARG were analyzed with the PlasFlow 1.1 [25] using a probability threshold 0.7 to check for a potential plasmidial location of ARGs. We also investigated the number and distribution of contigs with two or more putative ARGs to check for multiple resistance and/or whole ARG operons from environmental samples. Putative ARGs (and their respective contig) were submitted to Kaiju v1.6.2 [26] for taxonomic classification, with the option "run mode" set as "greedy". Later, we conducted a manual curation of each ARG to check for misannotations and inconsistencies. BLASTp searches [27] were performed against the non-redundant protein database, with default parameters. The results were considered under a e-value cutoff e^{-5}. Conserved domains (CDDs) and annotations in the source databases (ARDB [28], CARD [29], and UniProt [30]) were manually inspected. These results were used to classify misannotated/misclassified ARGs into different categories: (i) misannotated genes or gene families with low support for ARG prediction, i.e., all source database sequences exhibiting non-ARGs as top 5 BLASTp (against NR database) hits with e-value cutoff of e^{-5}. Included are especially cases with an unambiguously erroneous original annotation (examples are described in the results). All of these misannotated ARGs were removed from our database and the downstream analyses; (ii) housekeeping genes that confer resistance only when specifically mutated; (iii) housekeeping genes conferring resistance when overexpressed; (iv) regulatory sequences responsible for ARG activation or overexpression of housekeeping genes leading to a resistance phenotype. The ARG family descriptions of the source databases (mainly those of the CARD database) were used (in addition to literature information) to classify ARGs into this scenario; (v) sequences with both similarities to ARGs and non-ARGs, belonging to the same superfamily and/or sharing domains. BLASTp and CDD analysis were used to classify ARGs into this scenario in cases where the TARA sequences show, non-ARGs and no specific CDD domain for that ARG among the top 10 blastp hits.

ARGs quantification and statistical tests on metagenomic samples

Environmental ARGs identified were used as a reference for raw read mapping by BBMAP v37.90 (default parameters) [31] after manual curation. The coverage, in

terms of reads count per gene and the abundance, in terms of Fragments Per Kilobase per Million mapped reads (FPKM), of each ARG was then calculated for each sample by BBMAP. The Average Genome Size (AGS) and Genome Equivalents (GE) were estimated by the software MicrobeCensus v1.0.7 (default parameters) [32] to calculate Reads Per Kilobase per Genome equivalents (RPKG) as described [32]. The RPKG of an ARG in a metagenome was calculated by 1) counting the number of reads mapped to the ARG; 2) dividing (1) by the length of the ARG in kilobase pairs (kb); 3) dividing the result of (2) by the number of sequenced genome equivalents:

$$RPKG = \frac{Mapped\ reads/Gene\ Length\ (Kb)}{Genome\ equivalents},$$

where,

$$Genome\ equivalents = \frac{Library\ size\ (bp)}{AGS\ (bp)},$$

and library size is the total number of sequenced base pairs (bp).

RPKG values for all ORFs classified as the same ARG were summed for each sample. Environmental features, such as sample depth, biogeographic biomes, ocean and sea regions, and fractions, were used for sample grouping and statistical tests. Pairwise Tukey HSD and multivariate linear regression using OLS models were conducted in Python 3.6 using the library ‘statsmodels’. The OLS was performed considering the following formula:

ARG_{RPKG} ~ fraction + Latitude + Longitude + depth + temp_c + NO2NO3 + PO4 + SI+ Mean_Oxygen + Mean_Salinity + OG_Shannon

Where ARG_{RPKG} (the dependent variable) is the sum of RPKM of all ARGs in a given class, and all the dependent variables are the selected environmental features. A two-way ANOVA analysis was conducted on the coefficients obtained from the OLS regression to infer the significance of a feature. A Python Jupyter notebook with the code and the results for all the exploratory and statistical analyses is provided on GitHub [33].

Phylogenetic analysis of environmental ARGs

Phylogenetic analyses were performed on environmental nucleotide sequences identified as clinically relevant ARGs, such as MCR-related sequences, for which

reference sequences were retrieved from public databases (e.g., NCBI and deepARGdb). Multiple protein sequence alignments and phylogenetic trees were generated using the standard pipeline of Phylogeny.fr [34]. In short, sequences were aligned using MUSCLE (default parameters) [35], conserved blocks extracted with gblocks (default parameters) [36], and phylogenetic trees generated with phyML [37], using Whelan And Goldman (WAG) matrix substitution model and Approximate Likelihood-Ratio Test (ALRT) statistical test.

Database design and implementation

A manually curated MySQL database was created with the environmental ARGs described and all the subsequent analysis results. Data downloaded and processed as described above was parsed with Java 8 and stored in the database with Hibernate. The database model is also managed by Hibernate in Java. The code is available on GitHub (https://github.com/mSorok/ResistomeDB). The resulting database contains 5 main data tables ('orf', 'arg', 'sample', 'organism' and 'xref', containing cross-references between the different data sources) and 5 connection tables to map in a SQL engine-free way the correspondences between the items from different tables. We provide the SQL dump and the database schema at Zenodo (https://zenodo.org/record/3473960).

Dash web application for data exploration and visualisation

We developed a Python dashboard web application where the user can explore the results through interactive graphics (plotted with the plotly library). The app includes a geographical scatterplot, where it is possible to visualize the abundance of each ARG (or antibiotic class) selected by the user across all the samples in a world map; a boxplot, where environmental features can be chosen to group the samples and compare their abundances; a barplot with taxonomic classification of the selected ARG (different taxonomic levels for the visualization can be chosen); a scatterplot with marginal distribution plots and trend line (OLS), where the X-axis represents the selected ARG, and the Y-axis, the environmental variables selected by the user (e.g., oxygen concentration, salinity, temperature, depth, etc.). In addition, a table containing information for each ORF is displayed. The additional information includes ORF id, contig id, antibiotic class, deepARG probability value, plasmid classification by PlasFlow, taxonomic classification by Kaiju (on the deepest level), the abundance of additional ARG ORFs in the same contig and the total of ARG ORFs in the contig. A link to download the multi-fasta file of the selected ARG is also provided. The application can be accessed at http://resistomedb.com/. The code and data for the dash app can be accessed at https://github.com/rcuadrat/resistome_dash.

Pipeline and code availability

The code of the complete pipeline (Figure 1) is in Bash and Python and it is available at the project repository on GitHub [38].

Figure 1: Flowchart used for ARG classification
The single steps and data used in the pipeline applied for the analyses presented in this work.

Results and Discussion

Environmental ARG prediction and manual curation

A total of 41,249,791 ORFs were predicted from 15,600,278 assembled contigs by MetaGeneMark. These ORFs were used as input for ARG screening with the deepARG software [20], resulting in the classification of 116,425 TARA ORFs (0.28%) as putative ARGs, related to 594 clinically relevant ARGs that confer resistance to 28 antibiotic classes (classes defined in the deepARGdb). The number of contigs, ORFs, and putative ARGs from each oceanic region is available in Supplementary Table 1. It was necessary to conduct an extensive manual curation on the results due to misannotations and misclassifications of ARGs in the databases used by deepARG. This curated dataset represents an important resource for further studies, including evolutionary and comparative studies.
A total of 34 ARGs were identified as misannotated or with low-quality annotation in the source database, leaving 560 ARGs for further analyses. A prominent example of a misannotated ARG is the *msr*B gene: While the *msrB* classified as ARG encodes an ABC-F subfamily protein leading to erythromycin and streptogramin B resistance, the corresponding fasta sequence in CARD database [29] belongs to the *msrB* gene encoding methionine sulfoxide reductases B, not conferring antibiotic resistance. Another misannotated ARG is the *pat*A gene, an ABC transporter of *Streptococcus pneumoniae*, conferring resistance to fluoroquinolones, whose sequence is a putrescine aminotransferase (*pat*A) in the CARD database. A total of 99,205 ORFs identified as putative ARGs on the categories (ii), (iii), (iv) and (v) (see methods parts) were kept in the MySQL database for further studies, while they were not used in the quantification and statistical analyses. The category (ii) includes the identification of 10 families of housekeeping genes and the corresponding mutations that could infer resistance. Category (iii) included 9 ARGs whose overexpression can lead to resistance. For category (iv), we identified 41 regulatory sequences that have been

identified as responsible for ARG expression or over-expression of housekeeping genes, causing the resistance phenotype. For category (v) included 187 putative ARGs that cannot be distinguished from non-ARGs by similarity alone (mostly due to commonly shared domains, for example, ATPases). After the removal of these genes, a total of 13,163 ORFs (from the initial 116,425) classified as 313 ARGs were retained for quantification and further analysis (Supplementary Table 2).

The most frequent ARG (in number of ORFs) identified in the co-assembly dataset was Qac (multidrug efflux pumps named after their conferring resistance to quaternary ammonium compounds) with more than 2,500 overall occurrences, followed by TETB(60) (Figure 2). The latter is an ABC transporter that confers resistance to tetracycline and tigecycline identified in a human saliva metagenomic library [39]. The ORFs conferring resistance to tetracycline combined are the most widespread, with several Tet and TetA classes accounting for approximately 4,000 occurrences. Also, the most frequent ARG that confers resistance to beta-lactams was identified as K678_12262, with about 1,000 occurrences.

Figure 2: The 20 most frequent ARGs after manual curation (in number of ORFs on co-assembled contigs)
Number of ORFs detected in all metagenomes; the corresponding resistance to antibiotic classes is depicted in the upper right.

Environmental ARGs in chromosomes and plasmids

We found a total of 24,567 putative ARGs (24.76% of the ORFs considered for the downstream analysis) present in contigs classified as plasmids by PlasFlow, which indicates the potential of horizontal genetic transfer (HGT). The occurrence of HGT of ARGs was already described in clinical environments [40], wastewater treatment plants (activated sludge) [14,41], and in fertilized soil [42], but little is known about ARG HGT in aquatic environments, especially in open ocean regions. As discussed in the later section on *mcr* genes, it should be noted here that PlasFlow analyses bear a small chance (about 4%) to result in false positives as described [25], which especially could be the case with chromosomally integrated plasmids or very short contig sequence sizes.

Multiple resistance presence in environmental contigs

The presence of two or more ARGs in a single contig was analyzed to identify possible multi-resistant organisms. For this analysis, we only removed the ARGs from the category (i) (misannotated sequences) because the presence of putative ARGs in the same contig and/or plasmid can give us additional functional evidence. We identified 4,063 contigs with multiple putative ARGs in contigs classified as chromosomes (up to 11 ARGs in the same contig), and 741 in contigs classified as a plasmid (up to 5 ARGs in the same contig), suggesting the presence of multi-resistant microorganisms in these environments (Table 1). We cannot exclude the possibility of multiple ARGs in both ends of plasmidial contigs being, in fact, artefacts, such as pieces of the same ARG in a circular contig. From the 4192 with 2 ARGs, 74 showed the same annotation for both ARGs (33 classified as plasmid). In figure S1, we show the distribution of the ARGs in the two putative plasmids containing 5 ARGs each.

Table 1: Distribution of multiple ARGs in chromosome and plasmids (classified by PlasFlow).

Number of ARGs	in chromosome	in plasmid
2	3503	689
3	365	37
4	116	13
5	35	2
6	22	0
7	10	0
8	6	0
9	2	0
10	2	0

11	2	0

Taxonomic classification of environmental ARGs

We classified 97,244 ARGs (98.02%) up to at least one taxonomic level using Kaiju [26]. Alphaproteobacteria (37,360 sequences) was identified as the largest taxonomic unit, followed by Gammaproteobacteria (19,355 sequences). A total of 124 ARGs were classified as of viral origin. The most frequent taxonomic viral groups identified were Prymnesiovirus (21 ARGs) and *Chrysochromulina ericina* virus (CeV) (19 ARGs). However, all the 124 viral ARGs were classified into the category (v), and further investigations should be performed to confirm these findings. The presence of ARGs in phages and their potential HGT was described in a Mediterranean river [43], pig faecal samples [15], fresh-cut vegetables, and agricultural soil [16].

In the contig containing 11 ARGs (TARA_ANW-k99_1343221), nine were classified as HGW-Alphaproteobacteria-3 or HGW-Alphaproteobacteria-12, and as generic Alphaproteobacteria. The two residual ARGs were classified as belonging to *Parvibaculum lavamentivorans*, an alphaproteobacterial species first isolated from activated sludge in Germany [44]. A previous study showed the presence of ARGs in a strain of *Parvibaculum* from marine samples by functional metagenomics [7], which might indicate a broader ARG distribution among this clade. All ARGs from the other contig containing 11 ARGs (TARA_ANE-k99_4428305) were classified as *Micavibrio sp*., an obligately predatory bacterium exhibiting 'vampire-like' behavior on gram-negative pathogens [45]. First isolated from wastewater samples, this genus has been considered as a potential new therapeutic approach against multi-resistant bacteria [46], including *mcr-1* positive strains [46], because no species from the genus *Micavibrio* was found to be pathogenic for humans [45]. However, if *Micavibrio* species are confirmed to contain one or multiple ARGs, this would raise concerns about any clinical therapeutic approaches with these bacteria. One of the putative plasmids containing five ARGs (contig TARA_PSE-k99_4996023, Supplementary figure S1) showed a taxonomic agreement between the classification of all its ARGs, which were assigned to the species *Tistrella mobilis*. Strains of this species were isolated from Thailand wastewater [47] and the Red Sea [48]. The other contig containing 5 ARGs of plasmidial origin, was classified as *Halomonas desiderata*, a denitrifying bacterium first isolated from a municipal sewage treatment plant [49]. Two of the putative 5 ARGs in this contig were classified as DfrE and DfrA3, which confer resistance to trimethoprim. Previous work showed that another bacteria of the same genus (*Halomonas marisflavi* type strain) is resistant to trimethoprim in vitro [50]. However, in the same study, *Halomonas desiderata* did not show resistance to any of the antibiotics tested.

ARG abundance and statistical tests on metagenomic samples

In previous sections, we aimed to find and characterize ARGs in metagenomic contigs obtained from co-assembled samples (by oceanic regions). In this section, we aimed to quantify ARGs in individual samples, to understand their geographical distribution and the environmental features driving their abundance. The average genome size (AGS) of samples of fractions enriched for virus and girus showed biased and aberrant results for AGS (up to 395.4 megabase pairs). These results are because AGS values (calculated by MicrobeCensus [32]) are inversely proportional to the number of reads mapping to housekeeping gene markers, and such genes have low abundance in virus-enriched samples. Based on this information, we kept only the 293 non-virus-enriched sample runs for downstream quantitative analyses.

For example, comparing biogeographical biomes, quinolone, and bacitracin ARG classes were significantly more abundant in the coastal biome than in the westerlies biome (adjusted Tukey HSD p-values 0.0476 and 0.0027, respectively). Furthermore, fosmidomycin ARGs were significantly (adjusted Tukey HSD p-value 0.0011) more abundant in the coastal biome than in the trades biome (Figure 3, Supplementary Table 3). Quinolone ARGs were previously reported as highly abundant in Chinese coastal areas [51]. These results might indicate that quinolone, bacitracin, and fosmidomycin ARGs are under anthropogenic pressure in coastal environments, and future studies should be carried out to investigate this assumption in greater detail.

The pristine polar biome showed significantly higher RPKG values for polymyxin ARGs than any other biome. The antibiotics polymyxin B and E (also known as colistin) are the last-resorts against gram-negative bacteria when modern antibiotics are ineffective, especially in cases of multiple drug-resistant *Pseudomonas aeruginosa* or carbapenemase-producing Enterobacteriaceae [52,53]. We discuss mobilized colistin resistance genes (*mcr*) in greater detail in a separate section later in this manuscript.

Figure 3: Significantly different abundances of ARG classes from Oceanic Biomes. Tukey HSD comparing the log-transformed RPKG of ARG classes for four biomes of Tara Oceans study. A- Confidence interval of RPKG for Quinolone ARGs; B- Confidence interval of RPKG for Bacitracin ARGs; C- Confidence interval of RPKG for Fosmidomycin ARGs; D- Confidence interval of RPKG for Polymyxin ARGs. Reference for the test is in blue, and in red the biome significantly more different than the reference ($p < 0.05$). The reference was chosen as Coastal Biome due to its ecological relevance.

When comparing the abundances of ARG classes on marine provinces, we found a significant difference ($p < 0.05$) of bleomycin class in 2 Indian provinces when compared to most of the other provinces (Figure 4). Bleomycin resistance genes were previously reported to be in association with New Delhi Metallo-β-lactamase (*ndm*-1)

genes [54,55]. In this study, *ndm*-like genes (classified by deepARG as *ndm*-17 variant) were also found in greater abundance in Indian South Subtropical Gyre province. The first variant of *ndm* was identified in *Klebsiella pneumoniae* strain isolated from a Swedish patient who travelled to New Delhi, India [56]. Shortly after, it was spread globally in a few years and was also detected in other Enterobacteriaceae, which was a reason to classify NDMs as a potential worldwide public health problem [57].

Figure 4: Bleomycin ARG abundance in marine provinces. Tukey HSD comparing the RPKG of ARGs from the class bleomycin. Reference for the test is in blue and in red the biome significantly different from the reference (p <0.05). Confidence intervals are shown. The reference was chosen randomly.

In addition to the geographical location, we investigated the influence of other environmental parameters on the abundance of ARG classes. In our OLS models, the variables with significant p-values (< 0.05 ANOVA test) for the largest number of antibiotic classes were *fraction* (14 classes), *sampling depth*, and *Shannon-Wiener index* (11 classes each). *The fraction* is a categorical variable, and the smallest size fraction (0.22 µm - 0.45 µm) was used as a reference for computing the coefficients in the model. This fraction is enriched for free-living, non-aggregating bacteria, which are smaller than other size fractions. For most classes (11 of 14), at least one category of fraction showed positive coefficients. For three of them, all fractions showed significantly more ARGs than the smallest fraction (tetracycline, aminoglycoside, and fosmidomycin). This result may indicate that free-living bacteria, in general, have a lower abundance of ARGs than particle-associated bacteria. These results corroborate a previous study, in which the antagonistic activity among pelagic marine bacteria (i.e., production of antibiotics) was more common in particle-associated bacteria than free-living bacteria [58].

For sampling depth, 5 of 11 classes were negatively correlated, indicating an increased abundance of ARGs in the deep water. For the Shannon-Wiener index, the only negatively correlated was tetracycline, indicating an increased abundance of ARGs in samples with lower species richness.

The regression model for tetracycline presented the highest adjusted R^2 (0.666) of all classes, with *fraction*, *temperature*, and *sampling depth* the most significant variables. For polymyxin, the adjusted R^2 was the second highest (0.559), being *temperature*, *Shannon index*, and *sampling depth* the most significant variables.

In general, among the nutrients, *nitrite+nitrate* concentration (NO_2NO_3) was significant for the largest number of classes (7 classes), followed by *inorganic phosphate* $(PO_4)^{3-}$ (6 classes). Silicon (SI) was only significant for the classes' fosmidomycin and tetracycline.

The role of inorganic nutrients concentration in antibiotic resistance genes abundance is poorly understood and sometimes controversial. Some studies suggest that a high concentration of nutrients is negatively associated with ARGs since competitive interactions in nutrient-rich environments are less important [59]. However, the abundance of ARGs are increased in wastewater treatment plants [60] and agricultural soil receiving dairy manure [61], both environments rich in nutrients. Further studies should be conducted to better understand the role of different nutrients on the abundance of ARGs of different classes in both pristine oligotrophic and impacted environments. The supplementary table 4 shows all significant results of an ANOVA test on the coefficients of OLS for each class and supplementary table 5 shows all the OLS results. A QQ-plot of the OLS residuals is shown in supplementary figure 2.

Mobilized colistin resistance genes (*mcr*) and other polymyxin resistance genes

Most mechanisms that confer resistance to colistin act against modifications of the lipid A moiety of lipopolysaccharide (LPS), with the addition of l-ara4N and/or phosphoethanolamine (PEtN) to lipid A as the main mechanisms [62]. We found evidence for the occurrence of putative mobilized colistin resistance genes related to the recently discovered *mcr*-1 [63], which relies on the PEtN addition to lipid A. The Mcr-1 enzyme was described as 41% and 40% identical to the PEA transferases LptA and EptC, respectively, and sequence comparisons suggest that the active-site residues are conserved. However, until the discovery of the plasmid-borne *mcr-1* in *E. coli* from pig [63], colistin resistance has always been linked to chromosomally encoded genes with low or no possibility of horizontal transfer. Further studies showed a high prevalence of the *mcr-1* gene (e.g., 20% in animal-specific bacterial strains and 1% in human-specific bacterial strains in China) and the plasmid has been detected in several countries covering Europe, Asia, South America, North America and Africa [64–71]. Further *mcr* variants were described as *mcr*-1 to 9 until December 2019 [72,73]. In the present data, we detected 15 proteins classified as Mcr-1 by deepARG, most abundant in the Atlantic Southwest Shelves Province, followed by its adjacent region, Antarctic Province (Figure 5). However, the employed version of deepARG did not classify these sequences into the more recently described Mcr-2 to 9. Therefore, we performed a phylogenetic analysis (Figure 6), which included sequences of different Mcrs (Mcr-1 to 5) and LptA (encoded by the gene *eptA*, used here as outgroup). The results suggested that 5 ORFs (from the genus *Psychrobacter*, family *Moraxellaceae* [74]) are close to the Mcr-1/2 clade with a support value of 1 (Figure 6). Members of the genus *Psychrobacter* were isolated from a wide range of habitats, including food, clinical samples, skin, gills, and intestines of fish, seawater, and Antarctic sea ice [75–79]. Importantly, at least two isolates from this genus were already reported to be resistant to colistin (*Psychrobacter vallis sp. nov.* and *Psychrobacter aquaticus sp. nov*), both isolated from Antarctica [76]. Coincidently, the

regions with greater RPKG mean values for MCR-1 abundance in our study were Southwest Atlantic and Antarctic Province. Our results support that *Psychrobacter* might be an ecological reservoir for the transfer of PEtN transferases to other pathogens, and further studies should be conducted to better understand the dynamics and evolution of ARGs in this genus. Also, some species of this genus were reported to cause opportunistic infections in humans, including at least one case reported to be associated with marine environment exposure [80]. In this context, it is therefore essential to increase monitoring by e.g., including screenings specific for *mcr*-related genes in these genera.

The residual Mcr sequences, mostly belonging to the *Thioglobus* genus, were phylogenetically farther away from Mcr-1/2 and might constitute new, distinct Mcr variants (Figure 6). Important to note is that the phylogenetically close relationship to Mcr sequences does not prove the function as a colistin-resistant gene, which awaits further experiments to confirm this role.

Figure 5: MCR-1 distribution on Tara Oceans marine provinces. The boxplot shows the sum of RPKG values for all MCR-1 ORFs**.**

Figure 6: Phylogenetic tree of MCR sequences
The phylogenetic tree was inferred using the standard pipeline from phylogeny.fr (phyML with the "WAG" model and statistical test approximate likelihood-ratio - Alrt for support values). Sequences for the outgroup *eptA* and clinical MCR-1 to MCR-5 were obtained from NCBI and used in addition to the sequences obtained from our results from Tara Oceans co-assemblies. The name of the Tara Oceans sequences displayed in the tree are defined with the id of sequence, co-assembly id, taxon name from Kaiju, and yes/no for plasmid classification from PlasFlow. The blue rectangles mark TARA sequences. The blue clade depicts the MCR-1/2 clade, the grey clade MCR-5, the green clade MCR-3/4, and the red clade *eptA*. The red circles mark sequences located on contigs classified as plasmids by PlasFlow. Numbers indicate alrt support values.

Only two *mcr* sequences were classified as present on plasmids via PlasFlow, which can be explained by the small size of many *mcr*-containing contigs (with eight of them smaller than 3 kb). Additionally, a false-negative result from PlasFlow could be a result of a re-integration of plasmidial sequences into the chromosome - or that these *mcr* genes may constitute an ancestor of the plasmidial *E. coli mcr* sequences, as suggested for *mcr*-1 encoded by *Moraxella* species [74]. The two ARGs classified as located on a plasmid are detected in contigs with a size of 2 kb and 38 kb. The former, classified as belonging to a *Thioglobus* species, is challenging to be validated as a plasmidial sequence due to its small size. The latter, classified as a sequence of a *Poseidonibacter* species, a marine group of bacteria recently reclassified from the *Arcobacter* genus, the latter containing several pathogenic species [81]. A toxin-antitoxin system is encoded two ORFs upstream of the *mcr* gene, which might be an indication for a plasmidial location. However, no further genes that are usually located on *Arcobacter* spp. plasmids [82] were found on this contig, hampering its correct

classification as a plasmidial *mcr*. That said, various mobile element genes located on this contig (Figure 7) strengthen the assumption that this contig is related to a mobile genetic region. An unusual synteny of *mcr*, *pap*2, and a downstream encoded *dagK* was observed (Figure 7), of which *dagK* only appears in *mcr-3* genetic environments [83]. Related genes (amino acid sequence identity of about 70%) with a conserved gene synteny are found in several *Arcobacter* species (Fig. 7). A few *Arcobacter* species with a similar *mcr* gene were susceptible to colistin treatment [84], arguing against a colistin resistance conferred by this gene product. Further research is necessary to confirm or refute colistin resistance in marine Poseidonibacter.

Fig 7: Genomic context of the *mcr* gene of contig TARA_PSE_k99_4834589

This contig was classified to be plasmidial by PlasFlow. Depicted are the first 13 ORFs from 28 of the whole contig, showing *mcr* and surrounding genes and including the mobile element related genes. DAG - Diacylglycerol, PAP2 - phosphatase PAP2 family protein, *mcr*- mobilized colistin resistance protein. Colour code: green - mobile element related gene, blue - Other/metabolic genes, yellow - DNA-related gene, light blue - Mcr-accessory genes, red - *mcr*, grey - hypothetical protein. Annotations from MetaGeneMark were manually refined using the conserved domains database and blastp against the SwissProt database. The taxonomy of *Arcobacter* species is stated as currently (December 2019) present in the Genbank taxonomy database.

The presence of *mcr*-related genes in both Antarctic and adjacent regions can also raise concerns about gene flow due to ice melting, a problem already discussed previously for other ARGs [85].

Conclusions

This study uncovers the diversity and abundance of ARGs in the global ocean metagenome, conferring putative resistance to 26 classes of antibiotics. The extensive analysis leads to a detailed taxonomic classification and distribution of ARGs abundance in different biomes. Our study also exposes the importance of monitoring coastal water for anthropogenic impact, since the inflow of antibiotic-resistant strains by, e.g., wastewater might provide input of ARGs by HGT for environmental strains. This study could also bear an impact on investigations dealing with the evolutionary history of ARGs, with the herein presented genes as ancestors of common ARGs in clinically relevant strains. Last but not least, the combination of multiple modern machine learning tools and other open-source data science libraries such as Dash and Plotly produced a valuable resource for the scientific community working on further studies on antibiotic resistance in different environments.

Availability of source code and requirements

- Project name: ResistomeDB
- Project home page: https://resistomedb.com
- Operating system(s): Platform independent
- Programming language: Python
- Other requirements: none
- License: MIT
- RRID: SCR_018305

Availability of supporting data

Snapshots of our code and other supporting data are available in the *GigaScience* repository, GigaDB [86].

Acknowledgements

We thank Jorge Boucas and the Bioinformatics Core facility of Max Planck Institute of Biology of Ageing, for the use of the computational resources (HPC cluster) and the fruitful discussions in the initial analysis of this work.

Competing Interests

The authors declare no competing interests.

Author Contributions

RRCC, MS, BGA, TG and AMRD: Conceived and designed the analysis; **RC, MS and BGA**: Performed the analysis; **RRCC and MS**: Conceived and designed the database; **RRCC**: designed the web-application; All authors wrote the manuscript and revised it for significant intellectual content.

References

1. Aslam B, Wang W, Arshad MI, Khurshid M, Muzammil S, Rasool MH, et al. Antibiotic resistance: a rundown of a global crisis. Infect Drug Resist. 2018;11: 1645–1658. doi:10.2147/IDR.S173867
2. Tagliabue A, Rappuoli R. Changing Priorities in Vaccinology: Antibiotic Resistance Moving to the Top. Front Immunol. 2018;9. doi:10.3389/fimmu.2018.01068

3. Risso VA, Gavira JA, Mejia-Carmona DF, Gaucher EA, Sanchez-Ruiz JM. Hyperstability and Substrate Promiscuity in Laboratory Resurrections of Precambrian β-Lactamases. J Am Chem Soc. 2013;135: 2899–2902. doi:10.1021/ja311630a
4. Hall BG, Barlow M. Evolution of the serine β-lactamases: past, present and future. Drug Resist Updat. 2004;7: 111–123. doi:10.1016/j.drup.2004.02.003
5. Wright GD. The antibiotic resistome: the nexus of chemical and genetic diversity. Nat Rev Microbiol. 2007;5: 175–186. doi:10.1038/nrmicro1614
6. Aminov RI. The role of antibiotics and antibiotic resistance in nature. Environ Microbiol. 2009;11: 2970–2988. doi:10.1111/j.1462-2920.2009.01972.x
7. Hatosy SM, Martiny AC. The Ocean as a Global Reservoir of Antibiotic Resistance Genes. Appl Environ Microbiol. 2015;81: 7593–7599. doi:10.1128/AEM.00736-15
8. Yang Y, Li Z, Song W, Du L, Ye C, Zhao B, et al. Metagenomic insights into the abundance and composition of resistance genes in aquatic environments: Influence of stratification and geography. Environ Int. 2019;127: 371–380. doi:10.1016/j.envint.2019.03.062
9. McConnell MM, Hansen LT, Neudorf KD, Hayward JL, Jamieson RC, Yost CK, et al. Sources of Antibiotic Resistance Genes in a Rural River System. J Environ Qual. 2018;47: 997–1005. doi:10.2134/jeq2017.12.0477
10. Van Goethem MW, Pierneef R, Bezuidt OKI, Van De Peer Y, Cowan DA, Makhalanyane TP. A reservoir of 'historical' antibiotic resistance genes in remote pristine Antarctic soils. Microbiome. 2018;6. doi:10.1186/s40168-018-0424-5
11. Hayward JL, Jackson AJ, Yost CK, Truelstrup Hansen L, Jamieson RC. Fate of antibiotic resistance genes in two Arctic tundra wetlands impacted by municipal wastewater. Sci Total Environ. 2018;642: 1415–1428. doi:10.1016/j.scitotenv.2018.06.083
12. Carney RL, Labbate M, Siboni N, Tagg KA, Mitrovic SM, Seymour JR. Urban beaches are environmental hotspots for antibiotic resistance following rainfall. Water Res. 2019;167: 115081. doi:10.1016/j.watres.2019.115081
13. Fresia P, Antelo V, Salazar C, Giménez M, D'Alessandro B, Afshinnekoo E, et al. Urban metagenomics uncover antibiotic resistance reservoirs in coastal beach and sewage waters. Microbiome. 2019;7. doi:10.1186/s40168-019-0648-z
14. Zhang T, Zhang X-X, Ye L. Plasmid metagenome reveals high levels of antibiotic resistance genes and mobile genetic elements in activated sludge. PloS One. 2011;6: e26041. doi:10.1371/journal.pone.0026041
15. Wang M, Liu P, Zhou Q, Tao W, Sun Y, Zeng Z. Estimating the contribution of bacteriophage to the dissemination of antibiotic resistance genes in pig feces. Environ Pollut Barking Essex 1987. 2018;238: 291–298. doi:10.1016/j.envpol.2018.03.024
16. Larrañaga O, Brown-Jaque M, Quirós P, Gómez-Gómez C, Blanch AR, Rodríguez-Rubio L, et al. Phage particles harboring antibiotic resistance genes in fresh-cut vegetables and agricultural soil. Environ Int. 2018;115: 133–141. doi:10.1016/j.envint.2018.03.019
17. Ibarbalz FM, Henry N, Brandão MC, Martini S, Busseni G, Byrne H, et al. Global Trends in Marine Plankton Diversity across Kingdoms of Life. Cell. 2019;179: 1084-1097.e21. doi:10.1016/j.cell.2019.10.008
18. Lima-Mendez G, Faust K, Henry N, Decelle J, Colin S, Carcillo F, et al. Determinants of community structure in the global plankton interactome. Science. 2015;348. doi:10.1126/science.1262073
19. Pesant S, Not F, Picheral M, Kandels-Lewis S, Le Bescot N, Gorsky G, et al. Open science resources for the discovery and analysis of *Tara* Oceans data. Sci Data. 2015;2: 150023. doi:10.1038/sdata.2015.23
20. Arango-Argoty G, Garner E, Pruden A, Heath LS, Vikesland P, Zhang L. DeepARG: a deep learning approach for predicting antibiotic resistance genes from metagenomic data. Microbiome. 2018;6: 23. doi:10.1186/s40168-018-0401-z
21. Tully BJ, Graham ED, Heidelberg JF. The reconstruction of 2,631 draft metagenome-assembled genomes from the global oceans. Sci Data. 2018;5: 170203.

doi:10.1038/sdata.2017.203
22. Delmont TO, Quince C, Shaiber A, Esen ÖC, Lee ST, Rappé MS, et al. Nitrogen-fixing populations of Planctomycetes and Proteobacteria are abundant in surface ocean metagenomes. Nat Microbiol. 2018;3: 804. doi:10.1038/s41564-018-0176-9
23. Companion Tables Ocean Microbiome EMBL. Available: http://ocean-microbiome.embl.de/data/OM.CompanionTables.xlsx
24. Zhu W, Lomsadze A, Borodovsky M. Ab initio gene identification in metagenomic sequences. Nucleic Acids Res. 2010;38: e132–e132. doi:10.1093/nar/gkq275
25. Krawczyk PS, Lipinski L, Dziembowski A. PlasFlow: predicting plasmid sequences in metagenomic data using genome signatures. Nucleic Acids Res. 2018;46: e35–e35. doi:10.1093/nar/gkx1321
26. Menzel P, Ng KL, Krogh A. Fast and sensitive taxonomic classification for metagenomics with Kaiju. Nat Commun. 2016;7: 11257. doi:10.1038/ncomms11257
27. BLAST. Available: https://blast.ncbi.nlm.nih.gov/
28. Liu B, Pop M. ARDB--Antibiotic Resistance Genes Database. Nucleic Acids Res. 2009;37: D443-447. doi:10.1093/nar/gkn656
29. Jia B, Raphenya AR, Alcock B, Waglechner N, Guo P, Tsang KK, et al. CARD 2017: expansion and model-centric curation of the comprehensive antibiotic resistance database. Nucleic Acids Res. 2017;45: D566–D573. doi:10.1093/nar/gkw1004
30. UniProt Consortium T. UniProt: the universal protein knowledgebase. Nucleic Acids Res. 2018;46: 2699. doi:10.1093/nar/gky092
31. Bushnell B. BBMap: A Fast, Accurate, Splice-Aware Aligner. Lawrence Berkeley National Lab. (LBNL), Berkeley, CA (United States); 2014 Mar. Report No.: LBNL-7065E. Available: https://www.osti.gov/biblio/1241166
32. Nayfach S, Pollard KS. Average genome size estimation improves comparative metagenomics and sheds light on the functional ecology of the human microbiome. Genome Biol. 2015;16: 51. doi:10.1186/s13059-015-0611-7
33. Cuadrat RRC. Resistome Statistical Analysis notebook. 2019. Available: https://github.com/rcuadrat/ocean_resistome/blob/master/exploring.ipynb
34. Dereeper A, Guignon V, Blanc G, Audic S, Buffet S, Chevenet F, et al. Phylogeny.fr: robust phylogenetic analysis for the non-specialist. Nucleic Acids Res. 2008;36: W465–W469. doi:10.1093/nar/gkn180
35. Edgar RC. MUSCLE: multiple sequence alignment with high accuracy and high throughput. Nucleic Acids Res. 2004;32: 1792–1797. doi:10.1093/nar/gkh340
36. Castresana J. Selection of Conserved Blocks from Multiple Alignments for Their Use in Phylogenetic Analysis. Mol Biol Evol. 2000;17: 540–552. doi:10.1093/oxfordjournals.molbev.a026334
37. Guindon S, Dufayard J-F, Lefort V, Anisimova M, Hordijk W, Gascuel O. New Algorithms and Methods to Estimate Maximum-Likelihood Phylogenies: Assessing the Performance of PhyML 3.0. Syst Biol. 2010;59: 307–321. doi:10.1093/sysbio/syq010
38. Cuadrat RRC. Resistome analysis project repository. 2019. Available: https://github.com/rcuadrat/ocean_resistome
39. Reynolds LJ, Roberts AP, Anjum MF. Efflux in the Oral Metagenome: The Discovery of a Novel Tetracycline and Tigecycline ABC Transporter. Front Microbiol. 2016;7. doi:10.3389/fmicb.2016.01923
40. Lerminiaux NA, Cameron ADS. Horizontal transfer of antibiotic resistance genes in clinical environments. Can J Microbiol. 2019;65: 34–44. doi:10.1139/cjm-2018-0275
41. Qiu Y, Zhang J, Li B, Wen X, Liang P, Huang X. A novel microfluidic system enables visualization and analysis of antibiotic resistance gene transfer to activated sludge bacteria in biofilm. Sci Total Environ. 2018;642: 582–590. doi:10.1016/j.scitotenv.2018.06.012
42. Peng S, Dolfing J, Feng Y, Wang Y, Lin X. Enrichment of the Antibiotic Resistance Gene tet(L) in an Alkaline Soil Fertilized With Plant Derived Organic Manure. Front Microbiol. 2018;9: 1140. doi:10.3389/fmicb.2018.01140
43. Calero-Cáceres W, Méndez J, Martín-Díaz J, Muniesa M. The occurrence of antibiotic

resistance genes in a Mediterranean river and their persistence in the riverbed sediment. Environ Pollut Barking Essex 1987. 2017;223: 384–394. doi:10.1016/j.envpol.2017.01.035

44. Schleheck D, Dong W, Denger K, Heinzle E, Cook AM. An α-Proteobacterium Converts Linear Alkylbenzenesulfonate Surfactants into Sulfophenylcarboxylates and Linear Alkyldiphenyletherdisulfonate Surfactants into Sulfodiphenylethercarboxylates. Appl Environ Microbiol. 2000;66: 1911–1916. doi:10.1128/AEM.66.5.1911-1916.2000
45. Dashiff A, Junka RA, Libera M, Kadouri DE. Predation of human pathogens by the predatory bacteria Micavibrio aeruginosavorus and Bdellovibrio bacteriovorus. J Appl Microbiol. 2011;110: 431–444. doi:10.1111/j.1365-2672.2010.04900.x
46. Dharani S, Kim DH, Shanks RMQ, Doi Y, Kadouri DE. Susceptibility of colistin-resistant pathogens to predatory bacteria. Res Microbiol. 2018;169: 52–55. doi:10.1016/j.resmic.2017.09.001
47. Shi B-H, Arunpairojana V, Palakawong S, Yokota A. Tistrella mobilis gen nov, sp nov, a novel polyhydroxyalkanoate-producing bacterium belonging to alpha-Proteobacteria. J Gen Appl Microbiol. 2002;48: 335–343. doi:10.2323/jgam.48.335
48. Xu Y, Kersten RD, Nam S-J, Lu L, Al-Suwailem AM, Zheng H, et al. Bacterial biosynthesis and maturation of the didemnin anticancer agents. J Am Chem Soc. 2012;134: 8625–8632. doi:10.1021/ja301735a
49. Berendes F, Gottschalk G, Heine-Dobbernack E, Moore ERB, Tindall BJ. Halomonas desiderata sp. nov, a new alkaliphilic, halotolerant and denitrifying bacterium isolated from a municipal sewage works. Syst Appl Microbiol. 1996;19: 158–167. doi:10.1016/S0723-2020(96)80041-5
50. Mata JA, Martínez-Cánovas J, Quesada E, Béjar V. A Detailed Phenotypic Characterisation of the Type Strains of Halomonas Species. Syst Appl Microbiol. 2002;25: 360–375. doi:10.1078/0723-2020-00122
51. Lu J, Zhang Y, Wu J, Wang J, Zhang C, Lin Y. Occurrence and spatial distribution of antibiotic resistance genes in the Bohai Sea and Yellow Sea areas, China. Environ Pollut. 2019;252: 450–460. doi:10.1016/j.envpol.2019.05.143
52. Velkov T, Roberts KD, Nation RL, Thompson PE, Li J. Pharmacology of polymyxins: new insights into an ‘old’ class of antibiotics. Future Microbiol. 2013;8: 711–724. doi:10.2217/fmb.13.39
53. Falagas ME, Kasiakou SK. Toxicity of polymyxins: a systematic review of the evidence from old and recent studies. Crit Care. 2006;10: R27. doi:10.1186/cc3995
54. Zhang L, Calvo-Bado L, Murray AK, Amos GCA, Hawkey PM, Wellington EM, et al. Novel clinically relevant antibiotic resistance genes associated with sewage sludge and industrial waste streams revealed by functional metagenomic screening. Environ Int. 2019;132: 105120. doi:10.1016/j.envint.2019.105120
55. Dortet L, Nordmann P, Poirel L. Association of the Emerging Carbapenemase NDM-1 with a Bleomycin Resistance Protein in Enterobacteriaceae and Acinetobacter baumannii. Antimicrob Agents Chemother. 2012;56: 1693–1697. doi:10.1128/AAC.05583-11
56. Yong D, Toleman MA, Giske CG, Cho HS, Sundman K, Lee K, et al. Characterization of a New Metallo-β-Lactamase Gene, blaNDM-1, and a Novel Erythromycin Esterase Gene Carried on a Unique Genetic Structure in Klebsiella pneumoniae Sequence Type 14 from India. Antimicrob Agents Chemother. 2009;53: 5046–5054. doi:10.1128/AAC.00774-09
57. Kumarasamy KK, Toleman MA, Walsh TR, Bagaria J, Butt F, Balakrishnan R, et al. Emergence of a new antibiotic resistance mechanism in India, Pakistan, and the UK: a molecular, biological, and epidemiological study. Lancet Infect Dis. 2010;10: 597–602. doi:10.1016/S1473-3099(10)70143-2
58. Long RA, Azam F. Antagonistic Interactions among Marine Pelagic Bacteria. Appl Environ Microbiol. 2001;67: 4975–4983. doi:10.1128/AEM.67.11.4975-4983.2001
59. Ponce-Soto GY, Aguirre-von-Wobeser E, Eguiarte LE, Elser JJ, Lee ZM-P, Souza V. Enrichment experiment changes microbial interactions in an ultra-oligotrophic

environment. Front Microbiol. 2015;6. doi:10.3389/fmicb.2015.00246
60. Ju F, Beck K, Yin X, Maccagnan A, McArdell CS, Singer HP, et al. Wastewater treatment plant resistomes are shaped by bacterial composition, genetic exchange, and upregulated expression in the effluent microbiomes. ISME J. 2019;13: 346–360. doi:10.1038/s41396-018-0277-8
61. McKinney CW, Dungan RS, Moore A, Leytem AB. Occurrence and abundance of antibiotic resistance genes in agricultural soil receiving dairy manure. FEMS Microbiol Ecol. 2018;94. doi:10.1093/femsec/fiy010
62. Baron S, Hadjadj L, Rolain J-M, Olaitan AO. Molecular mechanisms of polymyxin resistance: knowns and unknowns. Int J Antimicrob Agents. 2016;48: 583–591. doi:10.1016/j.ijantimicag.2016.06.023
63. Liu Y-Y, Wang Y, Walsh TR, Yi L-X, Zhang R, Spencer J, et al. Emergence of plasmid-mediated colistin resistance mechanism MCR-1 in animals and human beings in China: a microbiological and molecular biological study. Lancet Infect Dis. 2016;16: 161–168. doi:10.1016/S1473-3099(15)00424-7
64. Hasman H, Hammerum AM, Hansen F, Hendriksen RS, Olesen B, Agersø Y, et al. Detection of mcr-1 encoding plasmid-mediated colistin-resistant Escherichia coli isolates from human bloodstream infection and imported chicken meat, Denmark 2015. Eurosurveillance Online Ed. 2015;20: 1–5. doi:10.2807/1560-7917.es.2015.20.49.30085
65. Falgenhauer L, Waezsada S-E, Yao Y, Imirzalioglu C, Käsbohrer A, Roesler U, et al. Colistin resistance gene mcr-1 in extended-spectrum β-lactamase-producing and carbapenemase-producing Gram-negative bacteria in Germany. Lancet Infect Dis. 2016;16: 282–283. doi:10.1016/S1473-3099(16)00009-8
66. Webb HE, Granier SA, Marault M, Millemann Y, Bakker HC den, Nightingale KK, et al. Dissemination of the mcr-1 colistin resistance gene. Lancet Infect Dis. 2016;16: 144–145. doi:10.1016/S1473-3099(15)00538-1
67. Tse H, Yuen K-Y. Dissemination of the mcr-1 colistin resistance gene. Lancet Infect Dis. 2016;16: 145–146. doi:10.1016/S1473-3099(15)00532-0
68. Zhang R, Huang Y, Chan EW, Zhou H, Chen S. Dissemination of the mcr-1 colistin resistance gene. Lancet Infect Dis. 2016;16: 291–292. doi:10.1016/S1473-3099(16)00062-1
69. Mulvey MR, Mataseje LF, Robertson J, Nash JHE, Boerlin P, Toye B, et al. Dissemination of the mcr-1 colistin resistance gene. Lancet Infect Dis. 2016;16: 289–290. doi:10.1016/S1473-3099(16)00067-0
70. Arcilla MS, Hattem JM van, Matamoros S, Melles DC, Penders J, Jong MD de, et al. Dissemination of the mcr-1 colistin resistance gene. Lancet Infect Dis. 2016;16: 147–149. doi:10.1016/S1473-3099(15)00541-1
71. Malhotra-Kumar S, Xavier BB, Das AJ, Lammens C, Butaye P, Goossens H. Colistin resistance gene mcr-1 harboured on a multidrug resistant plasmid. Lancet Infect Dis. 2016;16: 283–284. doi:10.1016/S1473-3099(16)00012-8
72. Kieffer N, Royer G, Decousser J-W, Bourrel A-S, Palmieri M, Rosa J-MODL, et al. mcr-9, an Inducible Gene Encoding an Acquired Phosphoethanolamine Transferase in Escherichia coli, and Its Origin. Antimicrob Agents Chemother. 2019;63. doi:10.1128/AAC.00965-19
73. Hadjadj L, Baron SA, Olaitan AO, Morand S, Rolain J-M. Co-occurrence of Variants of mcr-3 and mcr-8 Genes in a Klebsiella pneumoniae Isolate From Laos. Front Microbiol. 2019;10. doi:10.3389/fmicb.2019.02720
74. Wei W, Srinivas S, Lin J, Tang Z, Wang S, Ullah S, et al. Defining ICR-Mo, an intrinsic colistin resistance determinant from Moraxella osloensis. PLOS Genet. 2018;14: e1007389. doi:10.1371/journal.pgen.1007389
75. Maruyama A, Honda D, Yamamoto H, Kitamura K, Higashihara T. Phylogenetic analysis of psychrophilic bacteria isolated from the Japan Trench, including a description of the deep-sea species Psychrobacter pacificensis sp. nov. Int J Syst Evol Microbiol. 2000;50: 835–846.

76. Bowman JP, Nichols DS, McMeekin TA. Psychrobacter glacincola sp. nov., a halotolerant, psychrophilic bacterium isolated from Antarctic sea ice. Syst Appl Microbiol. 1997;20: 209–215.
77. BOWMAN JP, CAVANAGH J, AUSTIN JJ, SANDERSON K. Novel Psychrobacter Species from Antarctic Ornithogenic Soils. Int J Syst Evol Microbiol. 1996;46: 841–848. doi:10.1099/00207713-46-4-841
78. JUNI E, HEYM GA. Psychrobacter immobilis gen. nov., sp. nov.: Genospecies Composed of Gram-Negative, Aerobic, Oxidase-Positive Coccobacilli. Int J Syst Evol Microbiol. 1986;36: 388–391. doi:10.1099/00207713-36-3-388
79. Yumoto I, Hirota K, Sogabe Y, Nodasaka Y, Yokota Y, Hoshino T. Psychrobacter okhotskensis sp. nov., a lipase-producing facultative psychrophile isolated from the coast of the Okhotsk Sea. Int J Syst Evol Microbiol. 2003;53: 1985–1989. doi:10.1099/ijs.0.02686-0
80. Bonwitt J, Tran M, Droz A, Gonzalez A, Glover WA. Psychrobacter sanguinis Wound Infection Associated with Marine Environment Exposure, Washington, USA - Volume 24, Number 10—October 2018 - Emerging Infectious Diseases journal - CDC. [cited 9 Sep 2019]. doi:10.3201/eid2410.171821
81. Pérez-Cataluña A, Salas-Massó N, Diéguez AL, Balboa S, Lema A, Romalde JL, et al. Revisiting the Taxonomy of the Genus Arcobacter: Getting Order From the Chaos. Front Microbiol. 2018;9. doi:10.3389/fmicb.2018.02077
82. Douidah L, Zutter LD, Nieuwerburgh FV, Deforce D, Ingmer H, Vandenberg O, et al. Presence and Analysis of Plasmids in Human and Animal Associated Arcobacter Species. PLOS ONE. 2014;9: e85487. doi:10.1371/journal.pone.0085487
83. Eichhorn I, Feudi C, Wang Y, Kaspar H, Feßler AT, Lübke-Becker A, et al. Identification of novel variants of the colistin resistance gene mcr-3 in Aeromonas spp. from the national resistance monitoring programme GERM-Vet and from diagnostic submissions. J Antimicrob Chemother. 2018;73: 1217–1221. doi:10.1093/jac/dkx538
84. Houf K, Devriese LA, Zutter LD, Hoof JV, Vandamme P. Susceptibility of Arcobacter butzleri, Arcobacter cryaerophilus, and Arcobacter skirrowii to Antimicrobial Agents Used in Selective Media. J Clin Microbiol. 2001;39: 1654–1656. doi:10.1128/JCM.39.4.1654-1656.2001
85. Edwards A. Coming in from the cold: potential microbial threats from the terrestrial cryosphere. Front Earth Sci. 2015;3. doi:10.3389/feart.2015.00012
86. Cuadrat RRC; Sorokina M; Andrade BG; Goris T; Dávila AMR: Supporting data for "Global ocean resistome revealed: exploring Antibiotic Resistance Genes (ARGs) abundance and distribution in TARA oceans samples " GigaScience Database. 2020. http://dx.doi.org/10.5524/100739

Figure 1

Click here to access/download;Figure;Figure_1.pdf

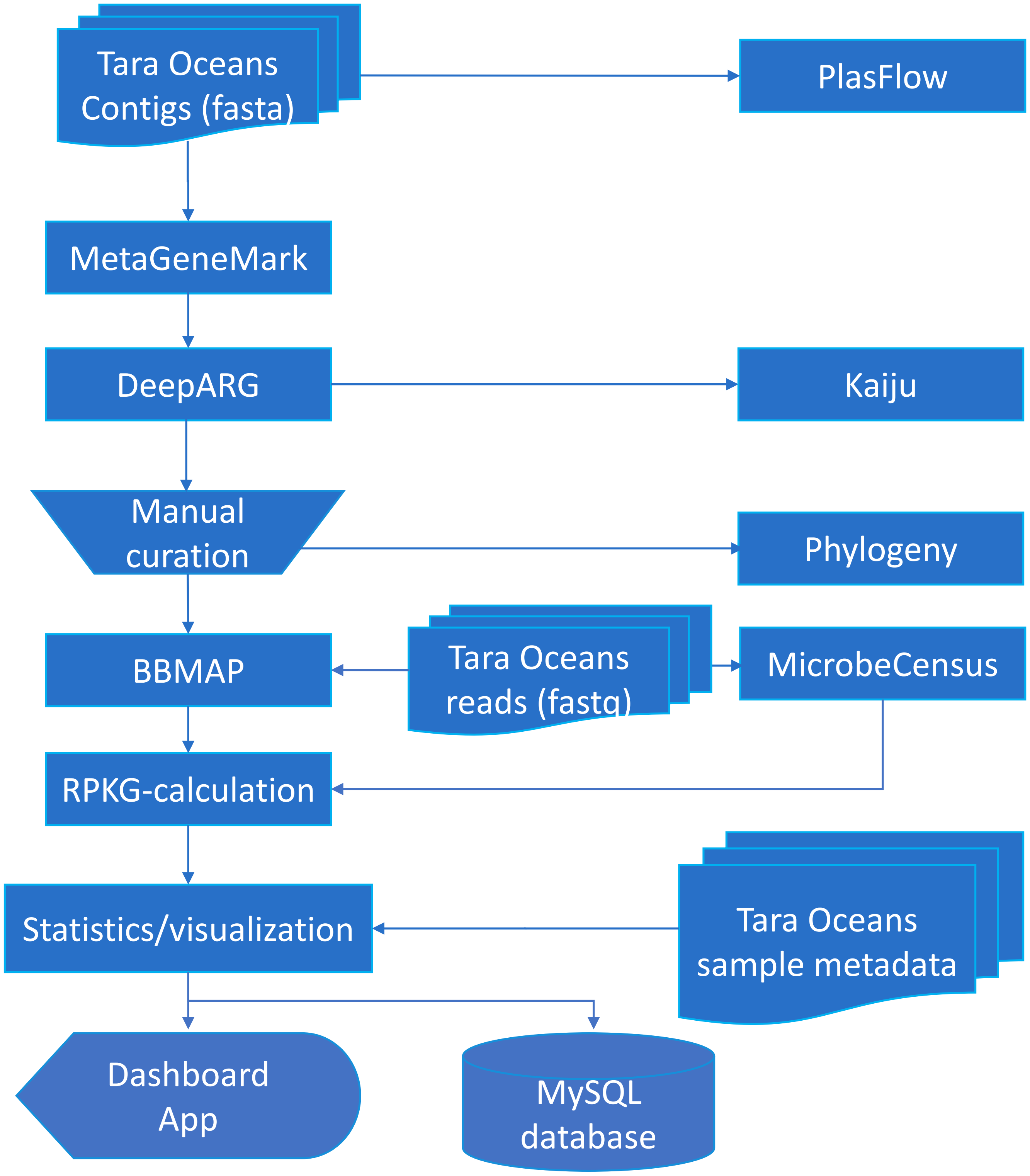

Figure 3

Click here to access/download;Figure;Figure_3.eps

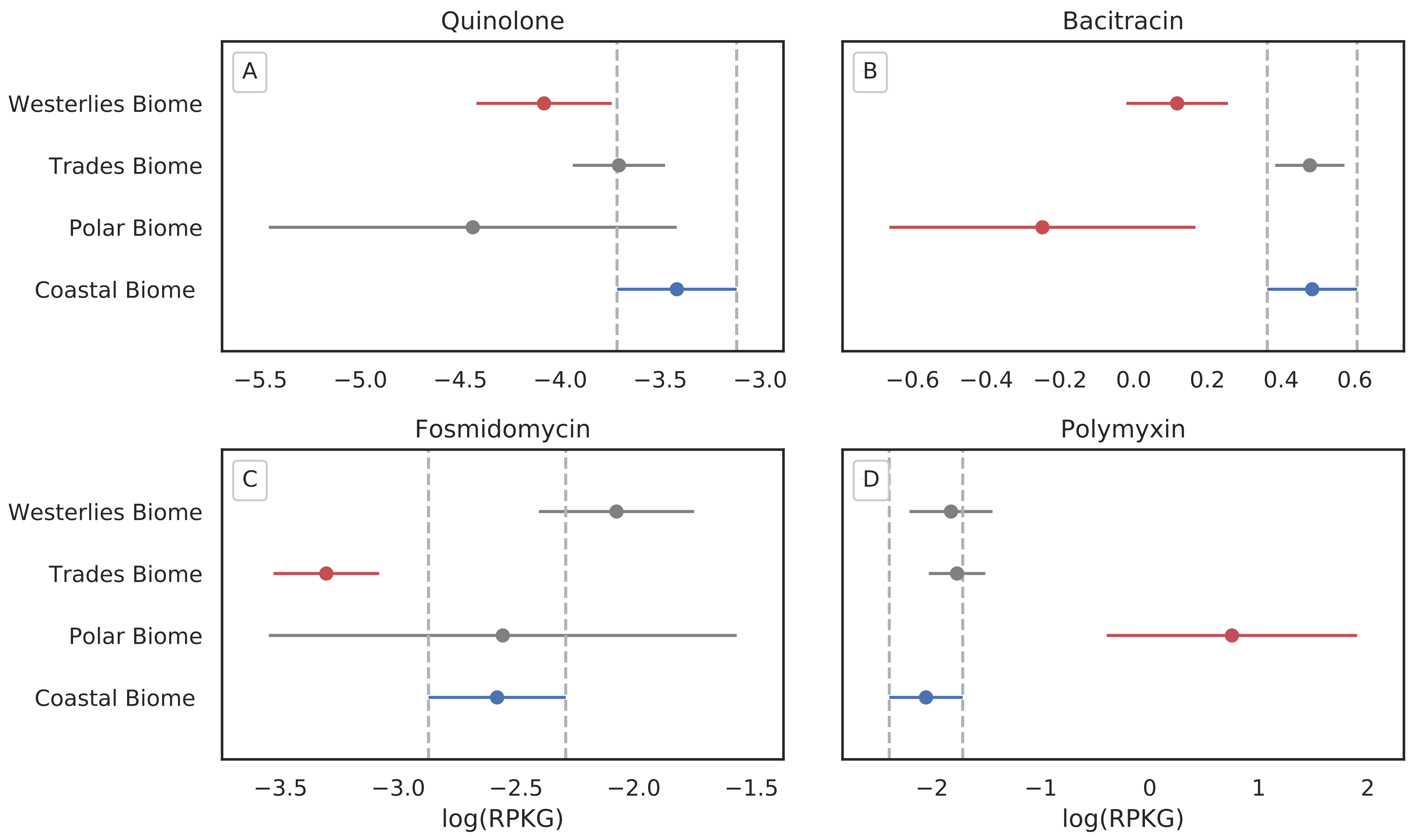

Click here to access/download;Figure;Figure_6.pdf

Figure 6

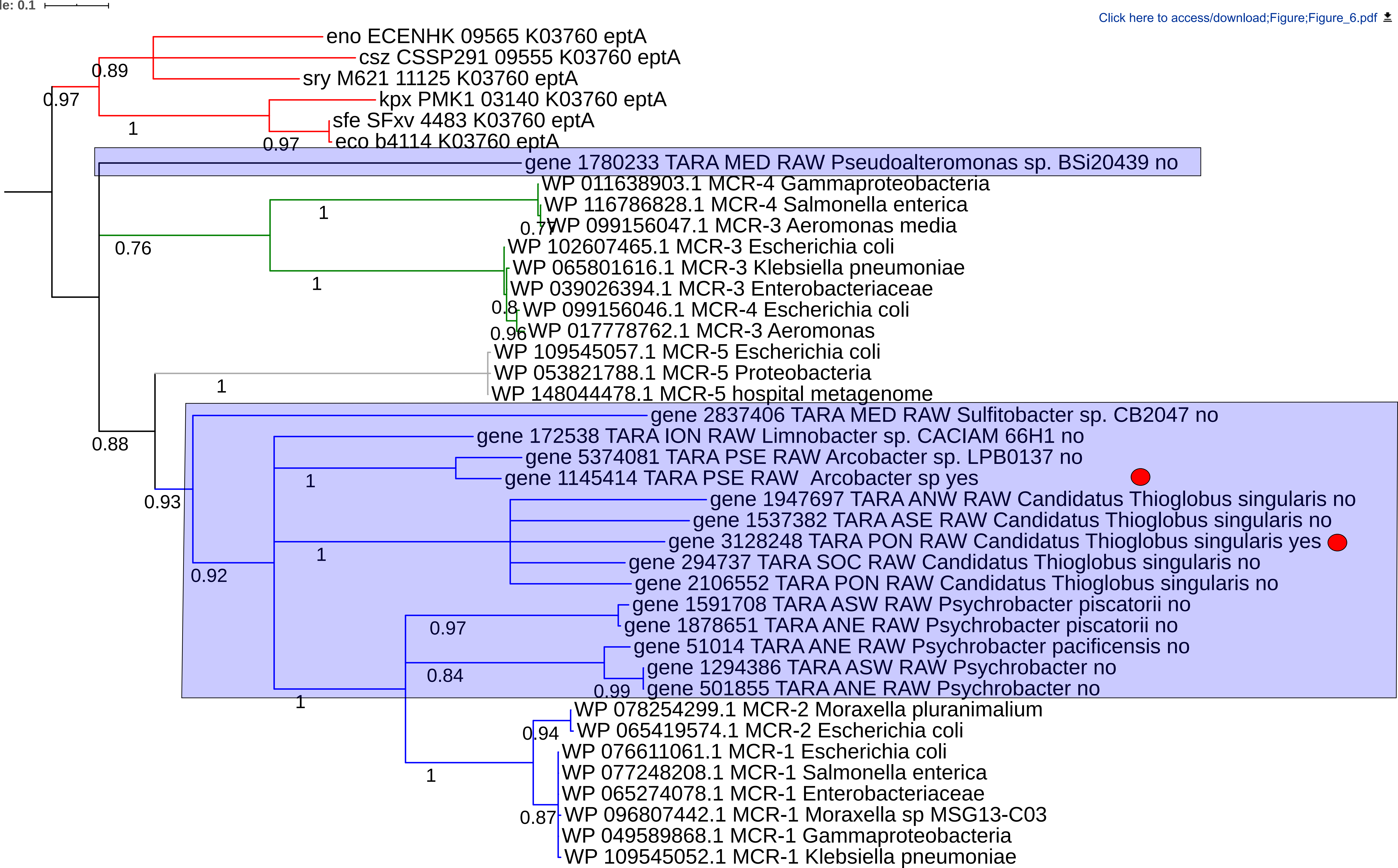

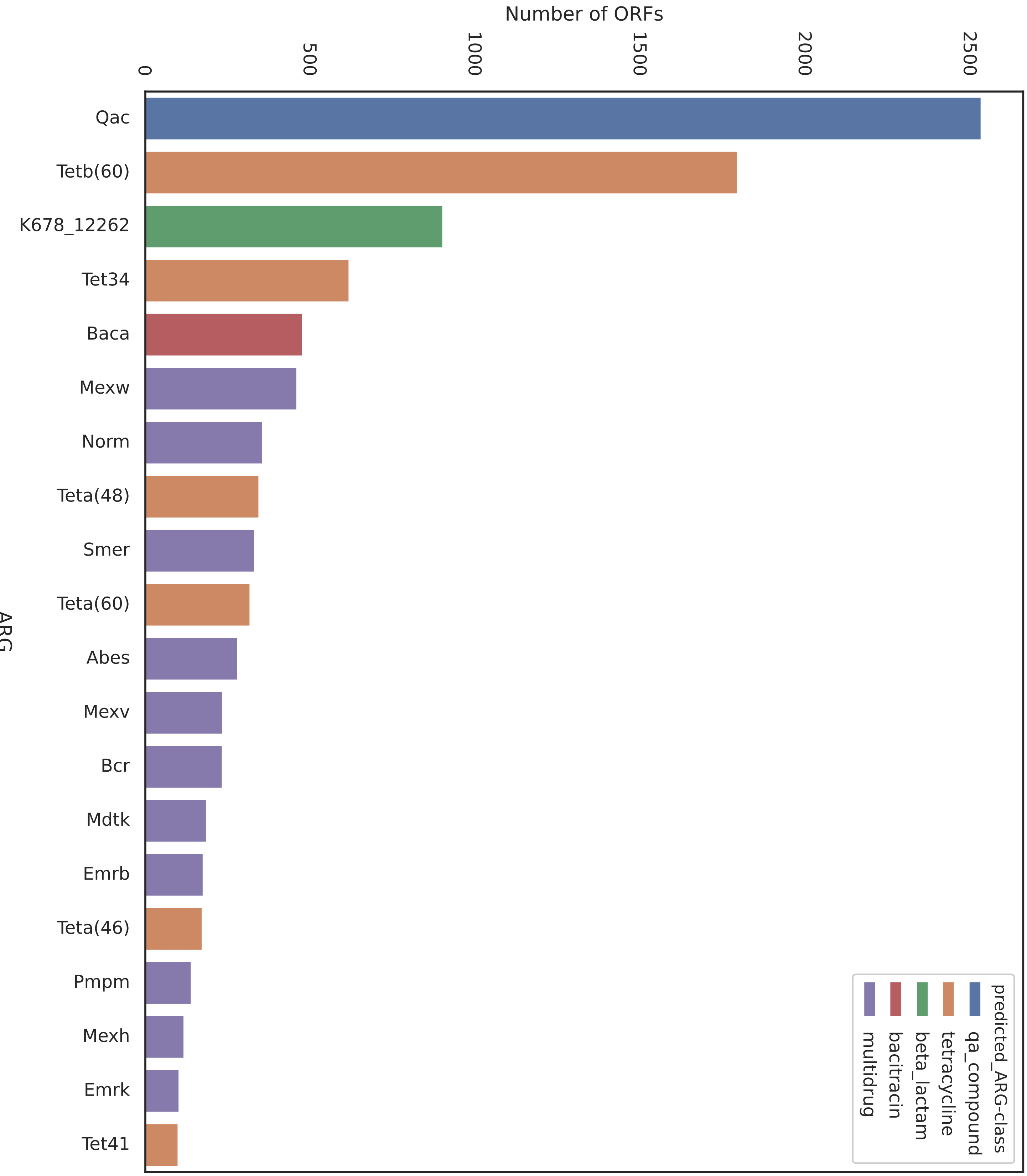

Figure 2
Click here to access/download;Figure;Figure_2_v2.eps

Figure 4

Click here to access/download;Figure;new_Figure_4_v2.eps

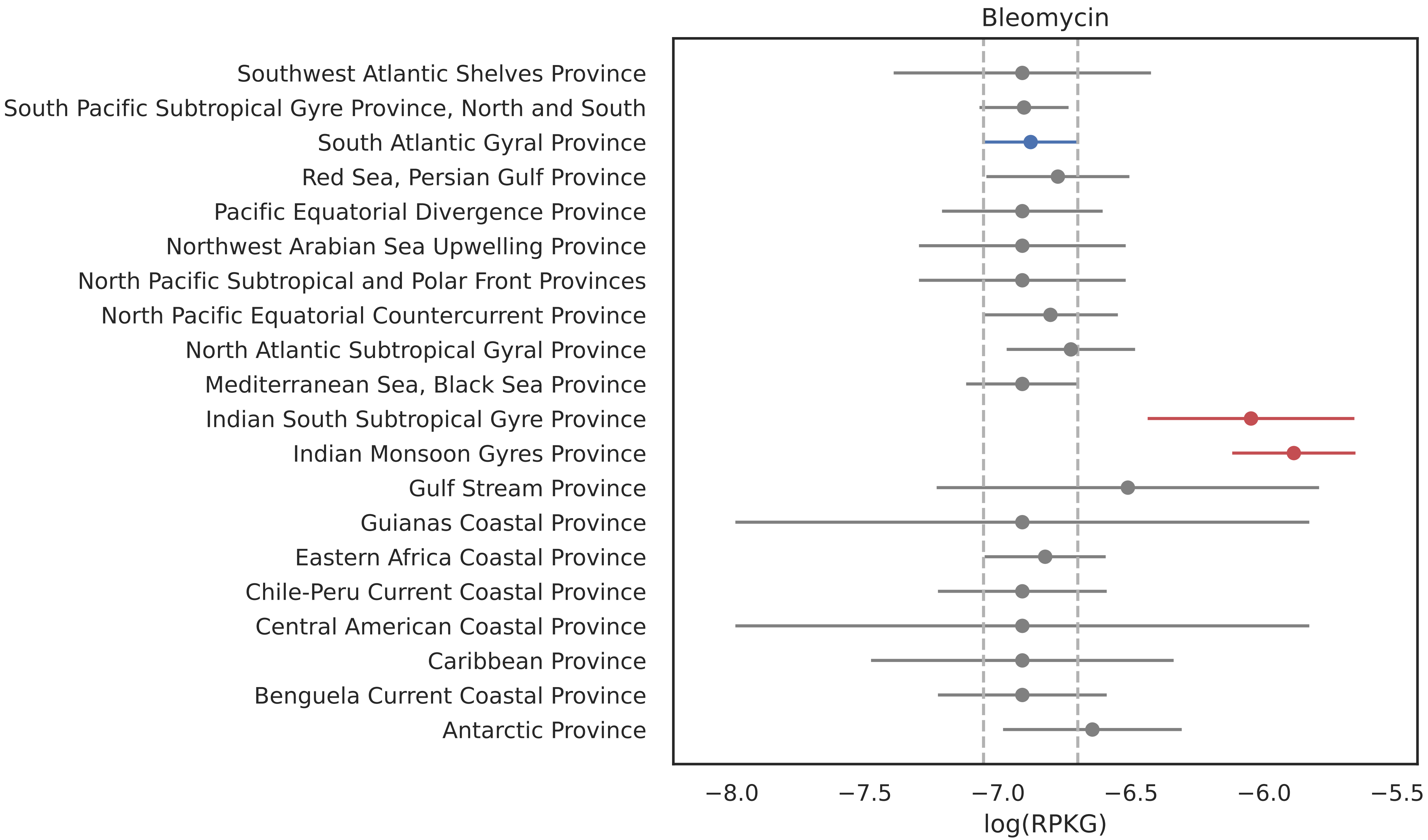

Figure 5

Click here to access/download;Figure;Figure_5_v2.eps

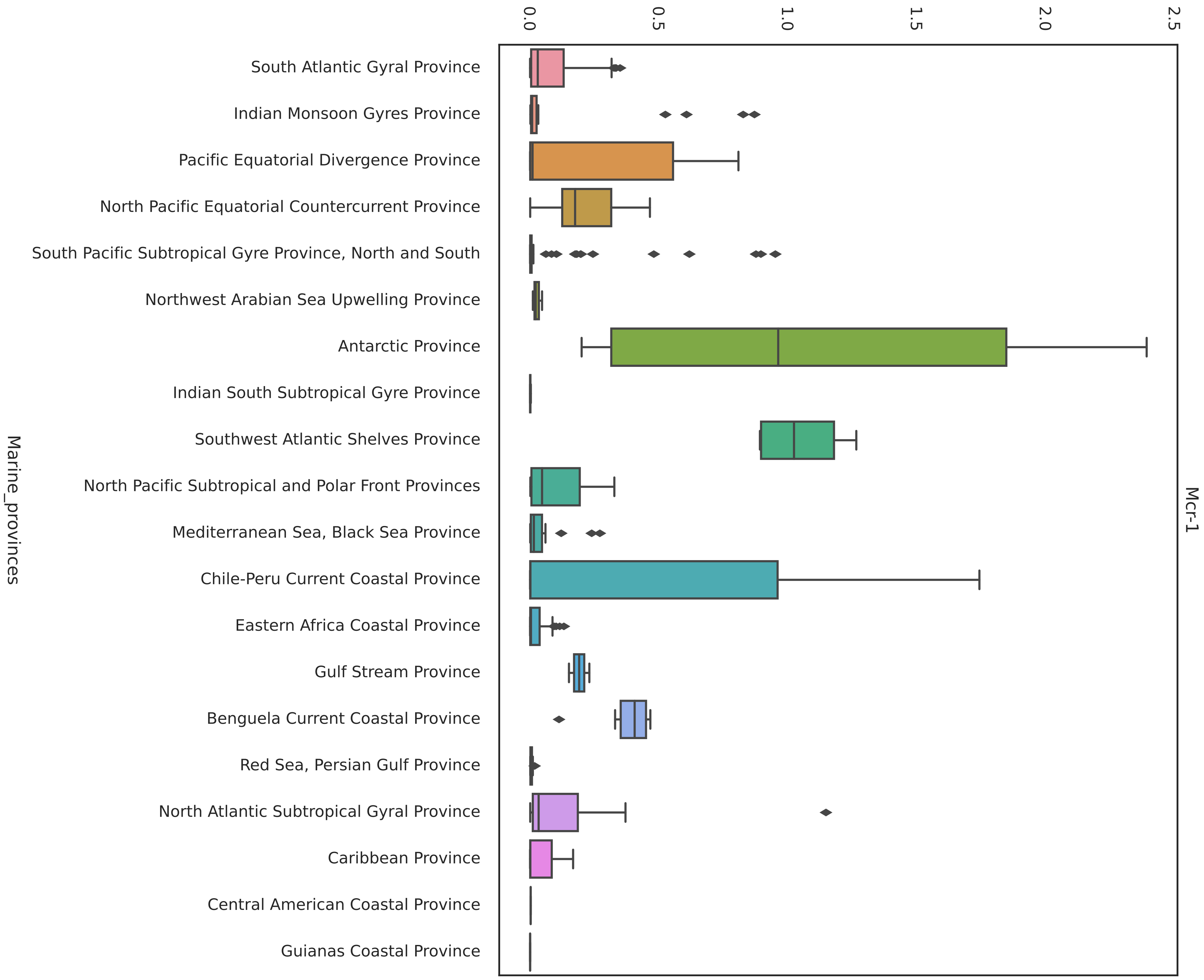

Figure S1

Click here to access/download
Supplementary Material
FigureS1.docx

Figure S2

Click here to access/download
Supplementary Material
figureS2.pdf

Table S1

Click here to access/download
Supplementary Material
stable1.docx

Table S2

Click here to access/download
Supplementary Material
stable2.xlsx

Table S3

Click here to access/download
Supplementary Material
stable3.xlsx

Table S4

Click here to access/download
Supplementary Material
stable4.xlsx

Table S5

Click here to access/download
Supplementary Material
stable5.xlsx

Figure 7

Click here to access/download
Supplementary Material
Figure_7.svg
