# Supplementary material for: Global ocean resistome revealed: Exploring antibiotic resistance gene abundance and distribution in TARA Oceans samples
Source: Gigascience. 2020 May 11;9(5):giaa046. doi: 10.1093/gigascience/giaa046 (PMC7213576; doi:10.1093/gigascience/giaa046)
Supplement: giaa046_Supplemental_Figures_and_Tables [file giaa046_supplemental_figures_and_tables.zip › FigureS1.docx]

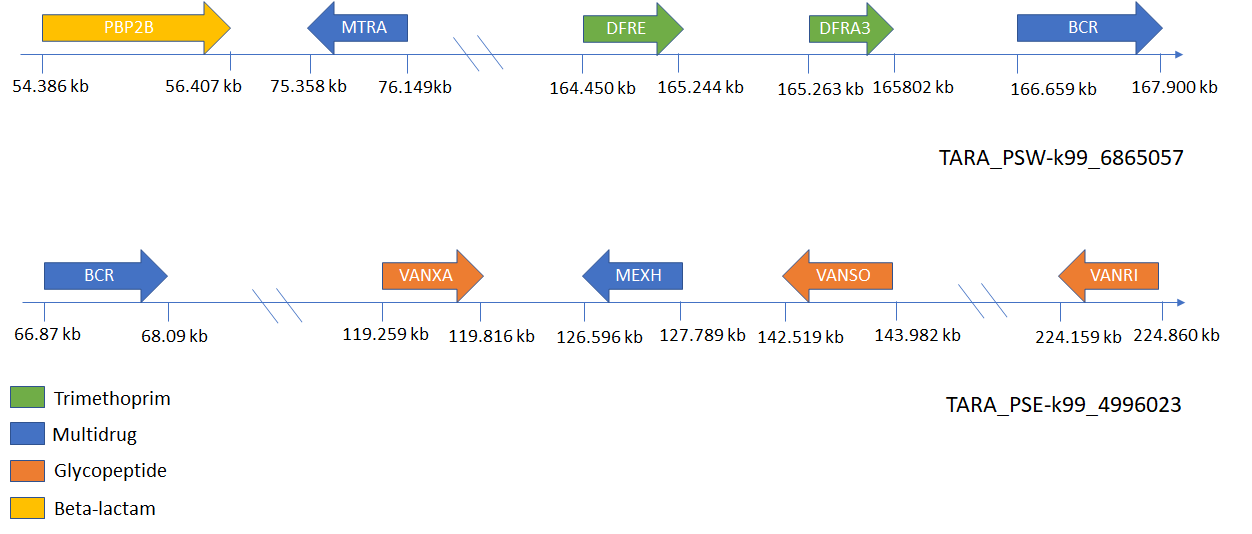


**Figure S1: ARGs distribution in the 2 plasmids showing 5 ARGs each.** The size of genes and distances are not scaled. **PBP2B**: methicillin-resistant PBP2; **MTRA**: transcriptional activator of the MtrCDE multidrug efflux pump; **DFRE**: dihydrofolate reductase; **DFRA3**: integron-encoded dihydrofolate reductase; **BCR**: Bicyclomycin resistance protein; **VANXA**: variant of VANX D,D-dipeptidase; **MEXH**: membrane fusion protein of the efflux complex MexGHI-OpmD; **VANSO**: variant of VANS, required for high-level transcription of other van glycopeptide resistance genes; **VANRI**: regulatory transcriptional activator in the VanSR regulator within the VanI glycopeptide resistance gene cluster.
