# Supplementary material for: Global ocean resistome revealed: Exploring antibiotic resistance gene abundance and distribution in TARA Oceans samples
Source: Gigascience. 2020 May 11;9(5):giaa046. doi: 10.1093/gigascience/giaa046 (PMC7213576; doi:10.1093/gigascience/giaa046)
Supplement: giaa046_Supplemental_Figures_and_Tables [file giaa046_supplemental_figures_and_tables.zip › figureS2.pdf]

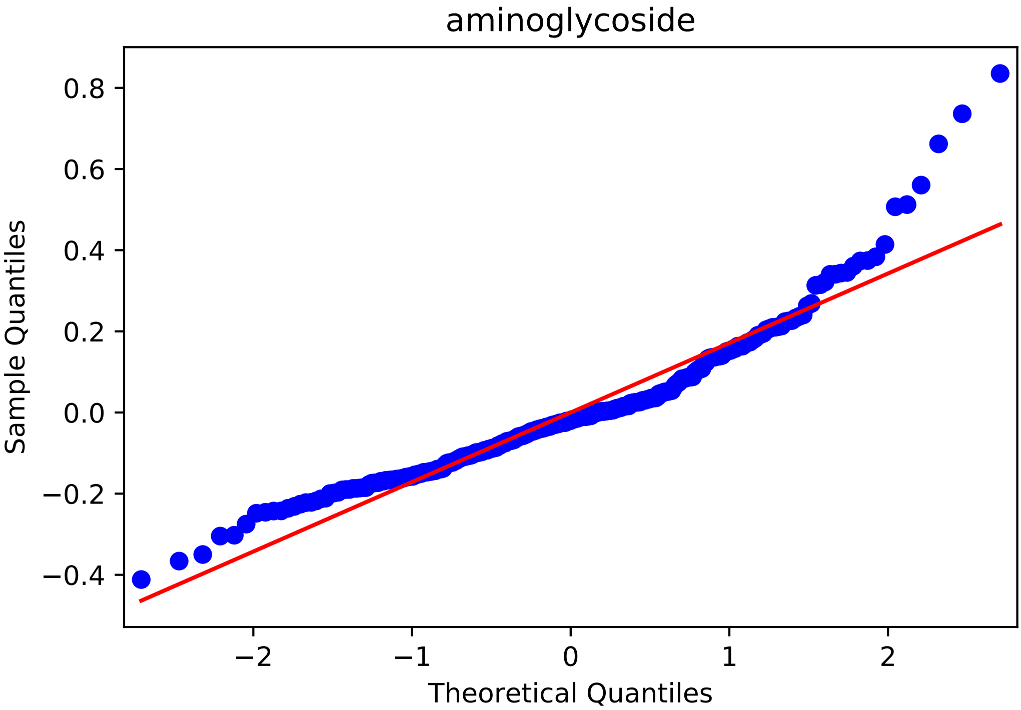

aminoglycoside
Sample Quantiles
Theoretical Quantiles
0.8
0.6
0.4
0.2
0.0
−0.2
−0.4
−2
−1
0
1
2

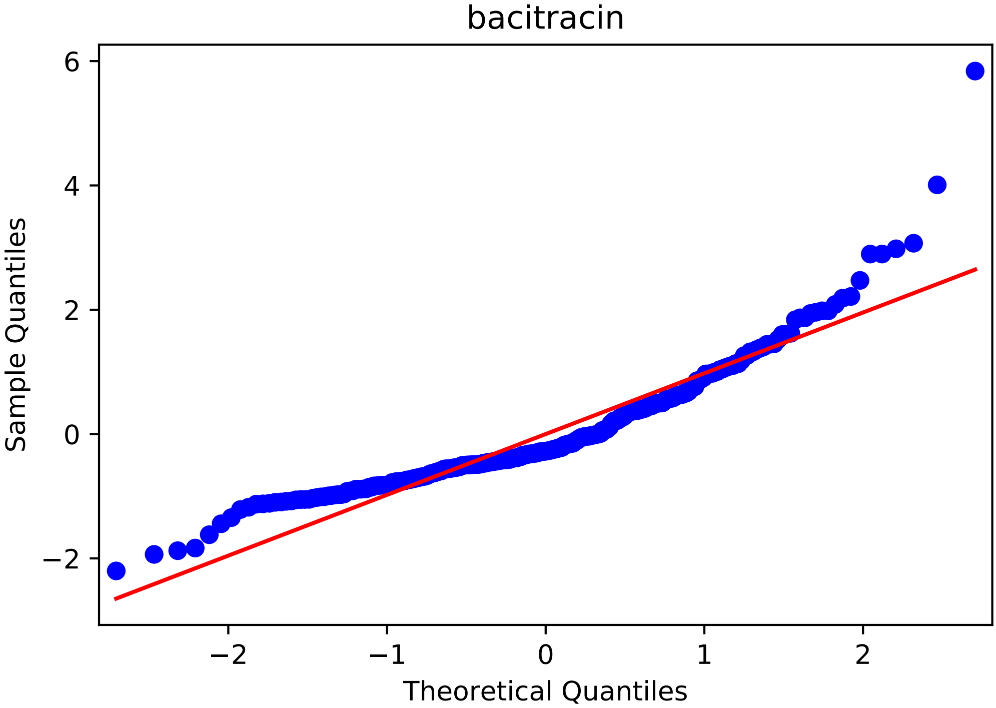

bacitracin
Sample Quantiles
Theoretical Quantiles
6
4
2
0
−2
−2
−1
0
1
2

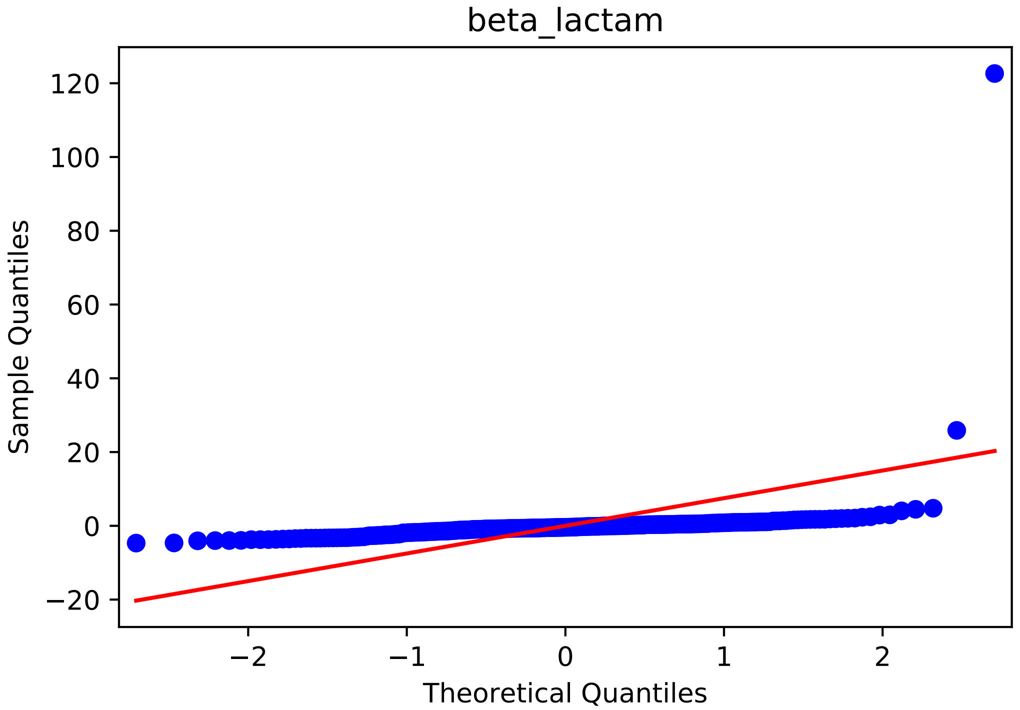
beta_lactam
Sample Quantiles
Theoretical Quantiles
120
100
80
60
40
20
0
−20
−2
−1
0
1
2

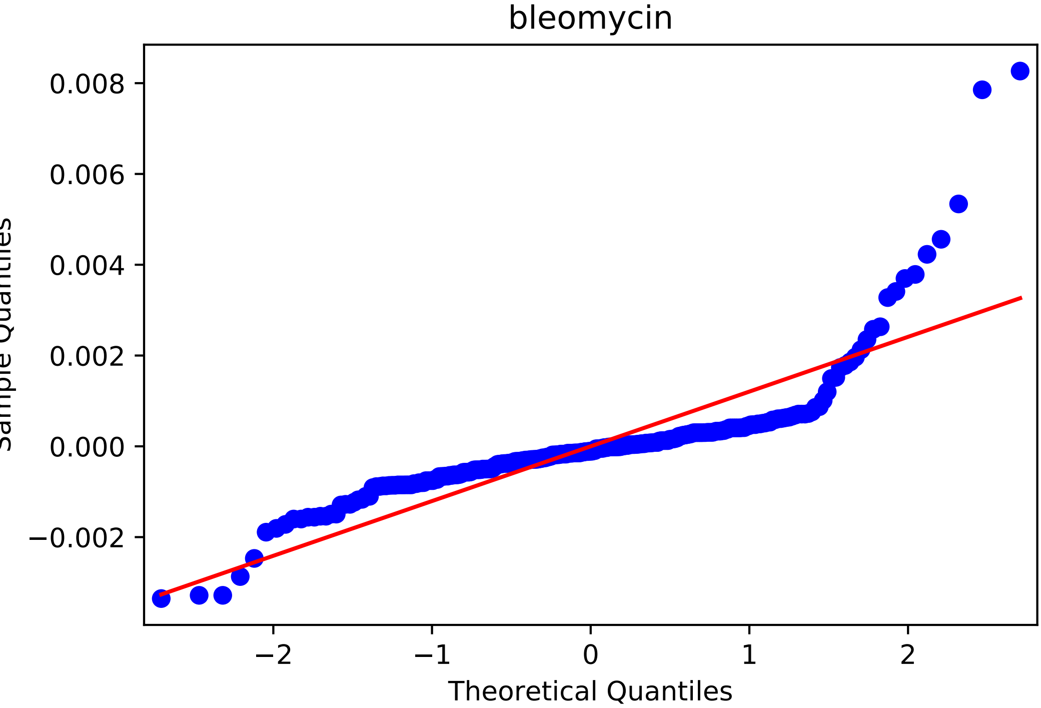
bleomycin
Sample Quantiles
0.008
0.006
0.004
0.002
0.000
−0.002
−2
−1
0
1
2
Theoretical Quantiles

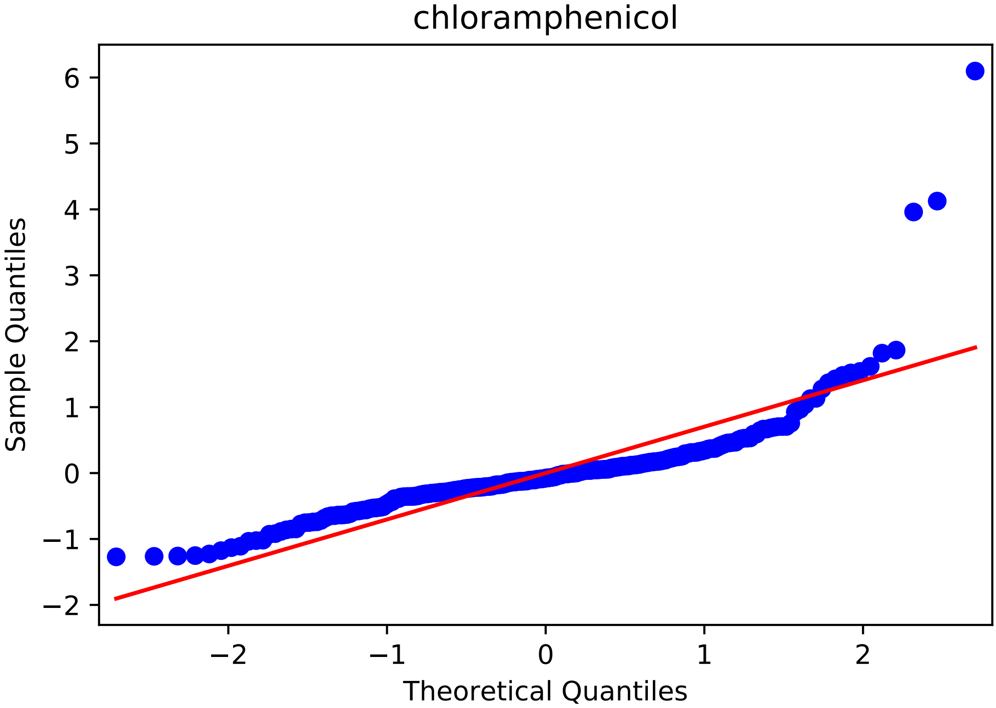
chloramphenicol
Sample Quantiles
Theoretical Quantiles
6
5
4
3
2
1
0
−1
−2
−2
−1
0
1
2

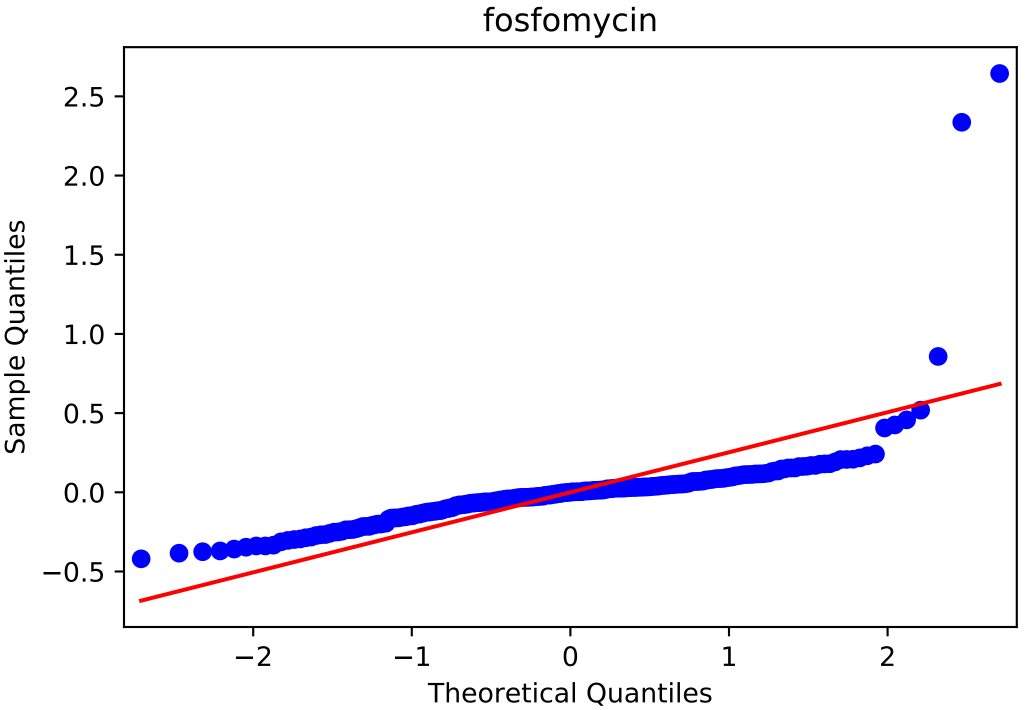
fosfomycin
Sample Quantiles
Theoretical Quantiles
2.5
2.0
1.5
1.0
0.5
0.0
−0.5
−2
−1
0
1
2

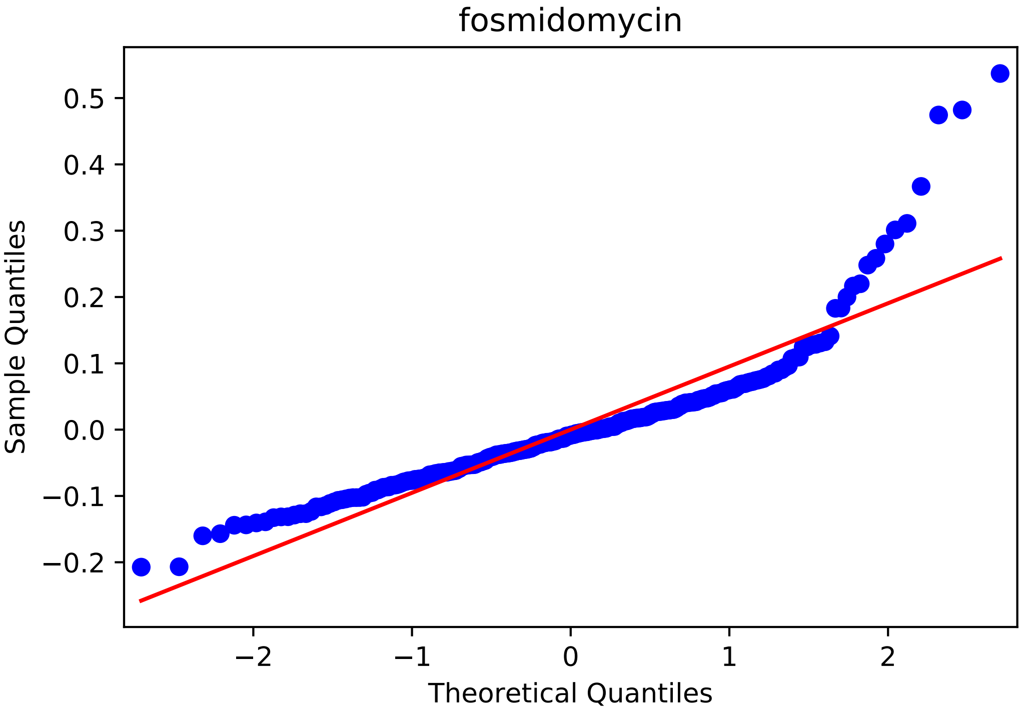
fosmidomycin
Sample Quantiles
Theoretical Quantiles
0.5
0.4
0.3
0.2
0.1
0.0
−0.1
−0.2
−2
−1
0
1
2

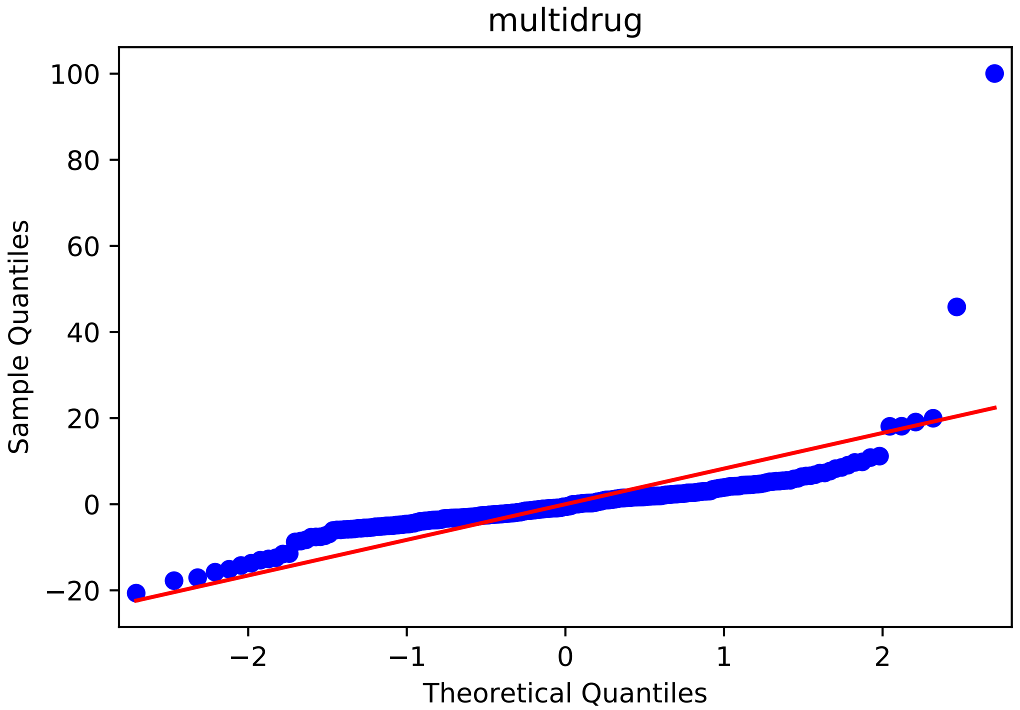
multidrug
Sample Quantiles
Theoretical Quantiles
100
80
60
40
20
0
−20
−2
−1
0
1
2

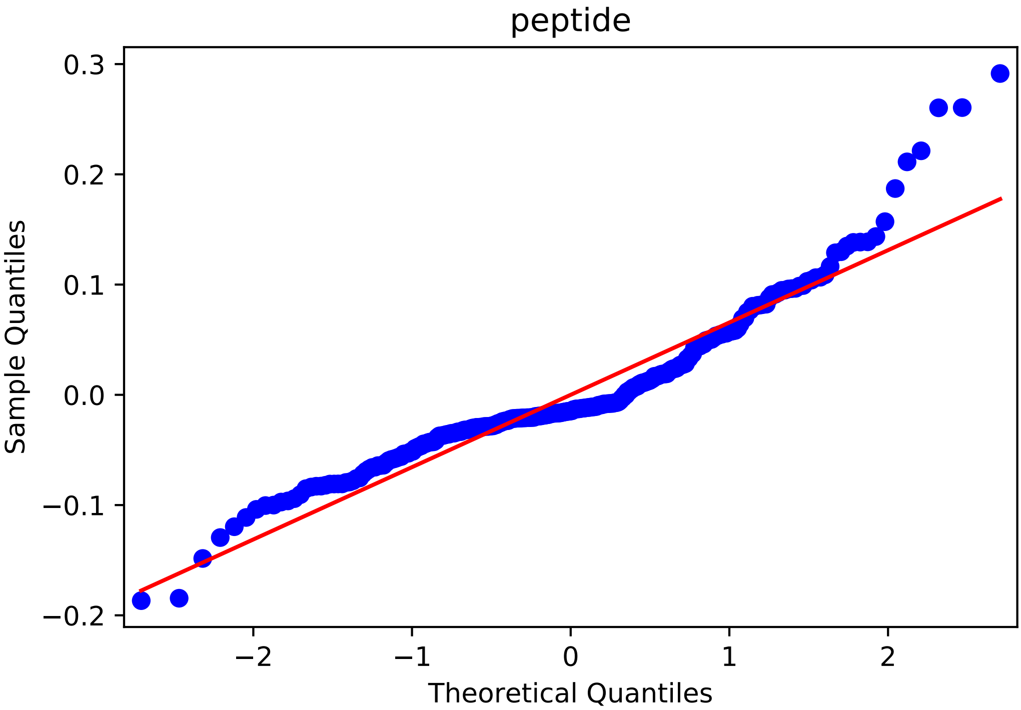
peptide
Sample Quantiles
Theoretical Quantiles
0.3
0.2
0.1
0.0
−0.1
−0.2
−2
−1
0
1
2

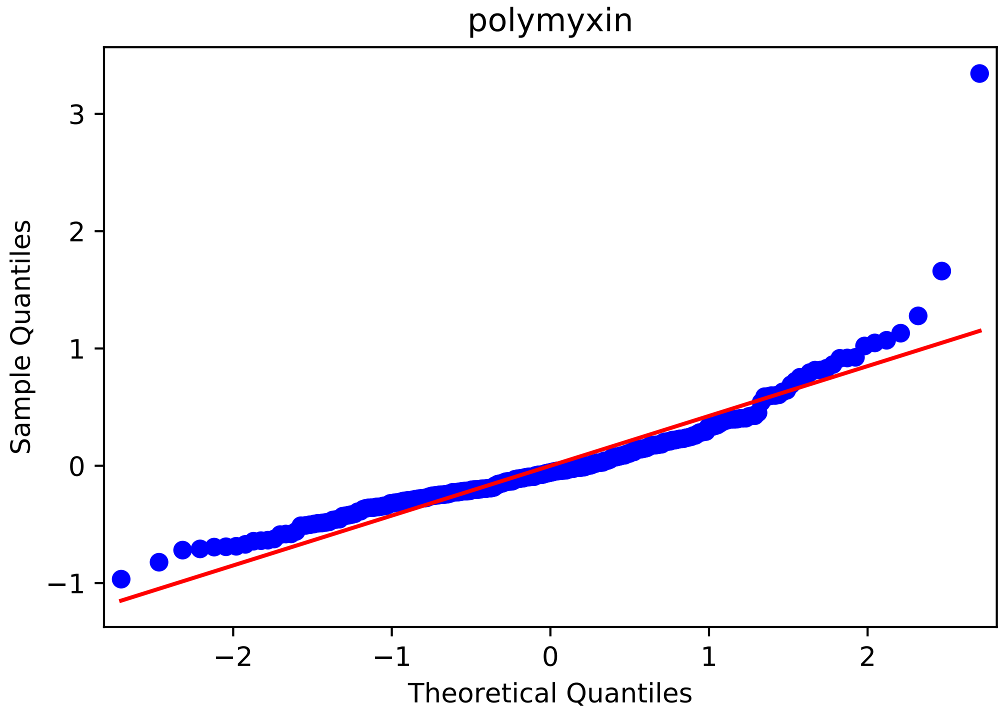
polymyxin
Sample Quantiles
Theoretical Quantiles
3
2
1
0
−1
−2
−1
0
1
2

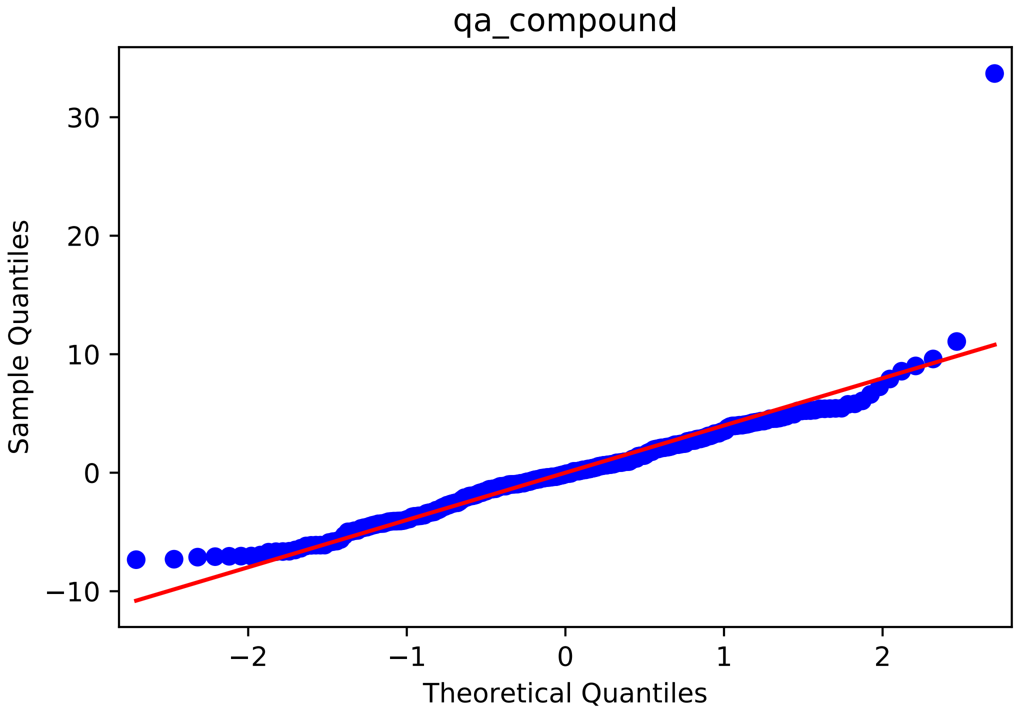

qa_compound
Sample Quantiles
Theoretical Quantiles
30
20
10
0
−10
−2
−1
0
1
2

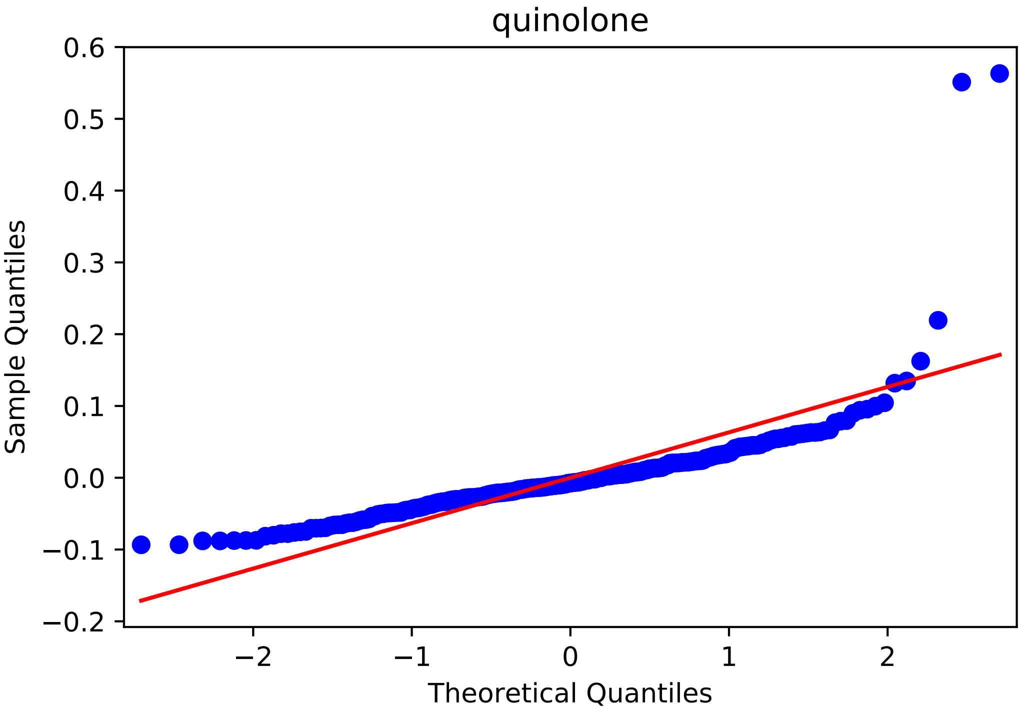

quinolone
Sample Quantiles
Theoretical Quantiles
0.6
0.5
0.4
0.3
0.2
0.1
0.0
−0.1
−0.2
−2
−1
0
1
2

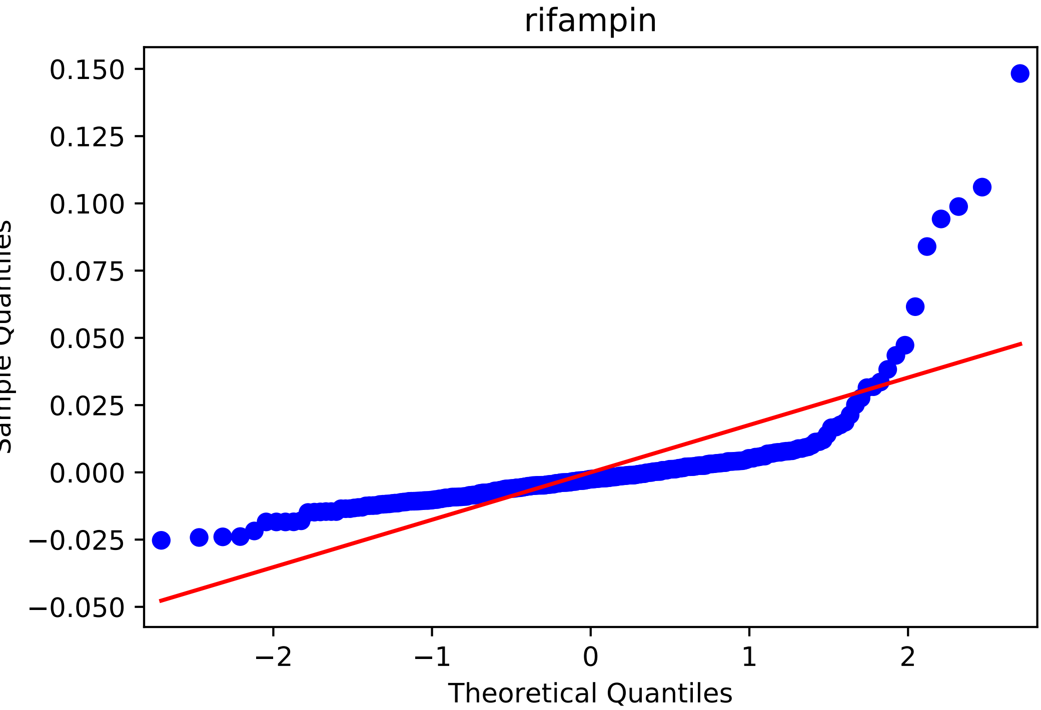
rifampin
0.150
0.125
0.100
0.075
0.050
0.025
0.000
−0.025
−0.050
Sample Quantiles
−2
−1
0
1
2
Theoretical Quantiles

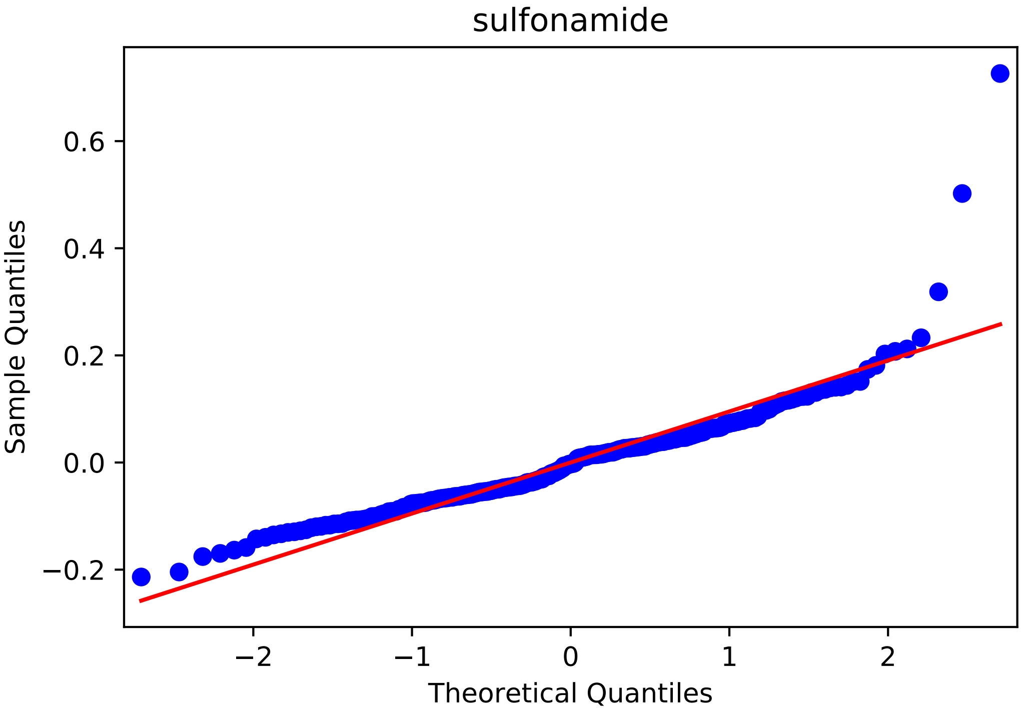
sulfonamide
Sample Quantiles
Theoretical Quantiles
0.6
0.4
0.2
0.0
−0.2
−2
−1
0
1
2

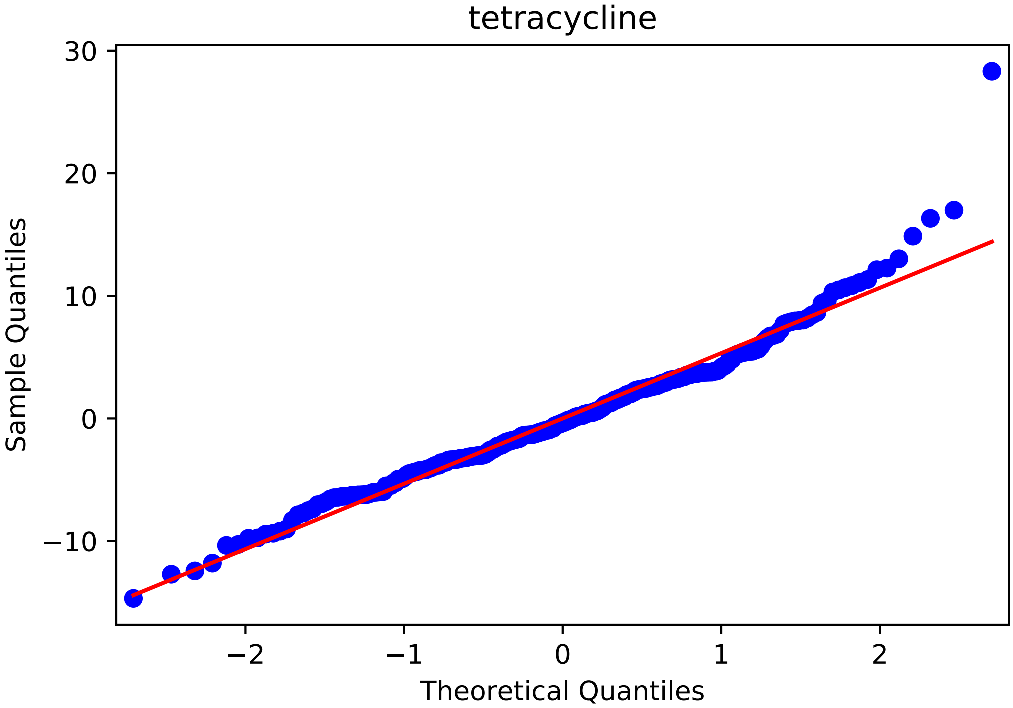

tetracycline
Sample Quantiles
Theoretical Quantiles
30
20
10
0
−10
−2
−1
0
1
2

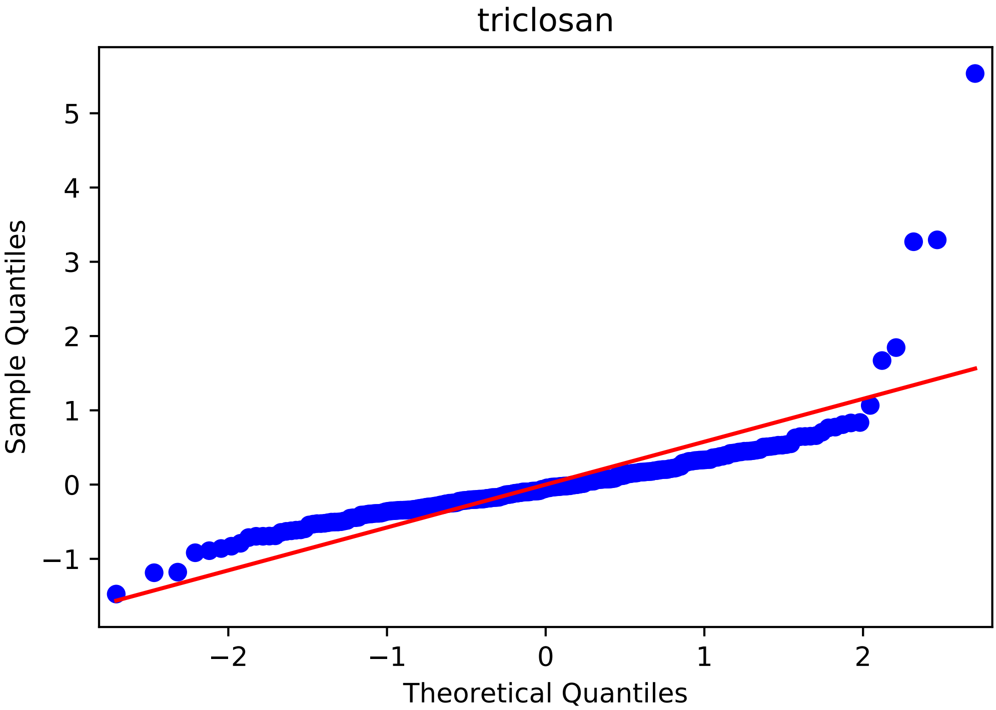
triclosan
Sample Quantiles
Theoretical Quantiles
5
4
3
2
1
0
−1
−2
−1
0
1
2

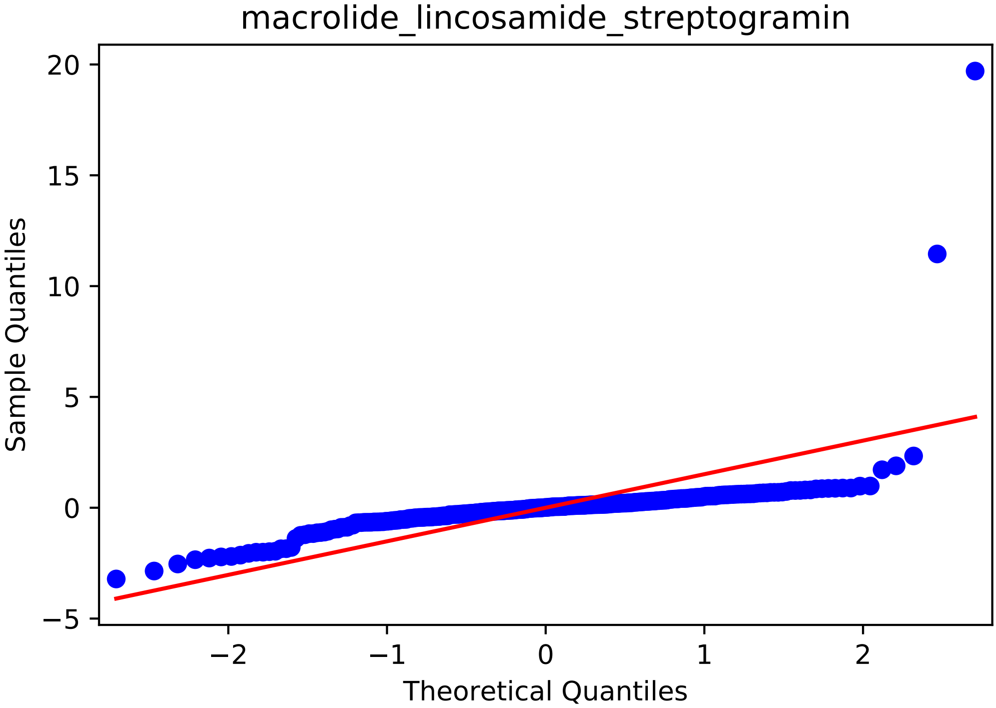
macrolide_lincosamide_streptogramin
Sample Quantiles
Theoretical Quantiles
20
15
10
5
0
−5
−2
−1
0
1
2
