# Supplementary material for: Global ocean resistome revealed: Exploring antibiotic resistance gene abundance and distribution in TARA Oceans samples
Source: Gigascience. 2020 May 11;9(5):giaa046. doi: 10.1093/gigascience/giaa046 (PMC7213576; doi:10.1093/gigascience/giaa046)
Supplement: giaa046_Supplemental_Figures_and_Tables [file giaa046_supplemental_figures_and_tables.zip › stable1.docx]

Supplementary Table 1: Number of contigs, ORFs and putative ARGs for each oceanic region (metagenomic co-assembly)

| **Sample** | **Region** | **Contigs** | **ORFs** | **#ARG** |
| --- | --- | --- | --- | --- |
| TARA_ANE_RAW | Atlantic North East | 1382239 | 3686619 | 11283 |
| TARA_ANW_RAW | Atlantic North West | 1267057 | 3308183 | 9994 |
| TARA_ASE_RAW | Atlantic South East | 766472 | 1842937 | 4955 |
| TARA_ASW_RAW | Atlantic South West | 989154 | 2502625 | 6902 |
| TARA_ION_RAW | Indian Ocean North | 1608737 | 4257000 | 9830 |
| TARA_IOS_RAW | Indian Ocean South | 1475271 | 3736229 | 9909 |
| TARA_MED_RAW | Mediterranean | 1146485 | 3344341 | 10160 |
| TARA_PON_RAW | Pacific Ocean North | 1663221 | 4368436 | 11814 |
| TARA_PSE_RAW | Pacific South East | 2722083 | 7155344 | 20587 |
| TARA_PSW_RAW | Pacific South West | 1124813 | 3080817 | 9224 |
| TARA_RED_RAW | Red Sea | 1039053 | 2937951 | 8924 |
| TARA_SOC_RAW | Southern Ocean | 415693 | 1029309 | 2843 |
| Total |  | 15,600,278 | 41,249,791 | 116,425 |
