# Supplementary material for: Global ocean resistome revealed: Exploring antibiotic resistance gene abundance and distribution in TARA Oceans samples
Source: Gigascience. 2020 May 11;9(5):giaa046. doi: 10.1093/gigascience/giaa046 (PMC7213576; doi:10.1093/gigascience/giaa046)
Supplement: giaa046_Response_to_Reviewer_Comments_Original_Submission [file giaa046_response_to_reviewer_comments_original_submission.pdf]

Author's Response To Reviewer Comments

Close

rev1

Minor

- [Abstract] ORFs abbreviation used before being defined

- Thanks for the correction. We added “open reading frame” for its first occurrence in the abstract.

- [Introduction] The statement "Antibiotic-resistant bacteria cause over 700,000 deaths per year, public health issue and an economic burden to the entire world" needs a reference.

The phrase was removed.

- [General] Given that deepArg is such a novel method it should be briefly explained. This would lead to a more readable paper for the non ML-specialist reader, furthermore it would make the title more consistent with the contents.

We added one paragraph explaining briefly the approach used by deepARG

- [Figure 6] Barely readable, the figure needs fixing by using a vectorized version or a higher DPI version.

Thanks for the note. Now we uploaded the high-resolution figures along with the new version of the text.

- [Material and Methods] To perform OLS regression by adding the RPKM of all ARGs (ARG_{RPKG}) sound a bit strange to me. A multi-input multi-output abalysis shloud be possible using some sort of PLS-regression technique.

We used the sum of all ARGs from each class to define the total ARGs RPKG in that class. As we are not doing a comparison between classes, this approach seems statistically correct.

- [Results and Discussion] "For plasmid classification, we relied on the results of PlasFlow, which only classified two of the sequences classified located on a plasmid. This can be explained by the". Sounds award to me due to word repetition,

Thanks for the revision, we agree that the phrase was confusing. We changed to better reading.

- [Conclusions] " conferring probably resistance to 26 classes of antibiotics. " Sounds award to me.

We rephrased for better reading.

Major: (Very personal)

- In my humble opinion the title seems to suggest that Machine Learning plays a key role in the discussion of the paper. However, the paper uses ml tools as black boxes without any kind of explanation or comparison with other methods. This can be solved by briefly explaining what the deepArg NN does and comparing the results with other ARG prediction models.

Attending the suggestion of the other reviewer, we changed the title of our paper excluding the machine learning, because the intention of the paper is not to compare and neither explain machine learning in

details, but rather use the tools for comprehensive data analyses

rev2

Here are some major concerns:

1. The website, http://resistomedb.com, seems not accessible (I just saw "Loading..." all the time).

We are sorry that it was not possible to access our tool. It was hosted by the free service herokuapp which turns the virtual machine off when not accessed for a while. After this, the machine has to be started again, which could result in a very long loading time. We changed the host service for more stable and fast access.

2. In the flowchart, "manual curation" only happens before the phylogeny analysis, I wonder if this is correct.

Indeed the position of “manual” curation was wrong in the first version of our MS. Thanks for pointing that. We fixed it in the new version.

3. In the flowchart, Tara raw reads (fastq) were used. However, I did not see a Data QC process.

The obtained fastq were checked with FASTQC and no problem was detected, so we assume the QC was done by the original authors before submission.

4. Many, many sections in the "Results" are describing methods (eg. manual curation), which should be relocated to "Methods". This also makes the readers quite difficult to follow the manuscript.

We did several modifications in both methods and results sections in order to improve the quality of the text.

5. I hope the authors could show a clearer storyline in the Result sections. For what purposes those analyses were done

We rewrote making the story more clear.

6. Figures and their legends should be self-explanatory. Eg., the ranges for each data point in Figure 3 were not explained (CI? SD?). How the "reference" is chosen is also not explained, and why sometimes the lower one was selected as the reference and in some other cases, the one with higher relative abundance was the reference.

We clarified the legend adding the information that the plot shows CI. We also added that the reference in the plot was chosen due to its ecological relevance

7. The method sections are not detailed enough for reproduction. For example:

a. I did not capture the detailed filtration criteria for the ARG ORF prediction. Short ORF fragments may influence the downstream analysis (including the multi-ARG presence on one contig analysis).

The MetaGeneMark tool classify as ORF only sequences larger than 60 nt. The original paper describing the tool discuss the performance of the tool for short sequences comparing to similar tools (https://academic.oup.com/nar/article/38/12/e132/2409881)

b. "a manual curation of each ARG" was not sufficiently described. It is also not clear if "Online BLASTp searches" and "Conserved domains (CDDs) and annotations in the source databases (ARDB, CARD and UniProt)" are associated with the manual curation. Also, the authors did not describe how these alignment results were used.

We re-wrote this part of methods in order to make it more clear

c. Some software parameters are. Eg., parameters, especially "threshold", for PlasFlow were not mentioned.

We included the parameters in the text for clarification

d. There should be a section for the summary for all statistical tests. Also, p-values were adjusted but the method was not mentioned.

The Tukey HSD is a post hoc test and it gives already the adjusted p-values corrected for multiple testing between the pairs of groups.

8. FPKM stands for "Fragments Per Kilobase per Million mapped reads". The authors made it wrong as "Fragments Per Kilobase Million". Thus, I worried if the basic data processing was credible.

We thank the reviewer for the correction. We fixed the text, and the calculation was done automatically by BBMAP, so there was no mistake in the calculation. In addition, we highlight that all the statistical analyses were done on RPKG instead of FPKM, which we provide only as additional results.

9. In the ARGRPKG model, statistical tests were mentioned but no significance cutoff was given. Variable selection needs to be addressed. I am not sure if all variables are independent. If not, the model could be problematic. Also, I am curious if the authors have tried to reduce the numbers of variables and evaluate the over-fitting.

Thank you for the suggestion. We checked the model for multiple collinearity and removed variables with high collinearity. The cutoff was always $p < 0.05$ and we added this information on the manuscript. Our idea with the model is rather find correlations than to make predictions so we did not did cross-validation to estimate overfit (also because the number of samples is not that high). On the other hand we provide now a number of diagnostic variables from the model, helping to evaluate better the performance.

10. Regarding Table 1, the authors declared that some contigs with 2 or more ARGs were annotated. However, this might because two ARG fragments from the same ARG were annotated on both edges of a circular contig.

We agree with this possibility, even if we think the chances are low, due to the limited size of the assembled contigs. However we include a sentence on the discussion about this possibility.

11. The authors should be very careful when describing "prevalence", "abundance" and "relative abundance". "abundance" was highly used in the manuscript; however, they should be relative abundance. And in Figure 2, the highest number of ORFs does not support "most abundant".

The RPKG metric is not considered a relative abundance metric, as stated in the original paper of MicrobeCensus, where they compare RPKG with relative abundance metrics. However, we agree with the comment on figure 2 and we rephrased it.

12. Tukey HSD tests were widely used in this study, and this is a test for parametric data to compare mean values. From Figure 5, I guess the RPKG relative abundance data should be quite nonparametric. I doubt this test could be applied for such data, as no data normalization was mentioned.

Thanks for pointing us for that. Indeed the distribution of the RPKG is not normal but rather log-normal. We addressed the problem by doing a natural log transformation of the values and the results are quite similar. We updated the figures and text.

13. Some conclusion seems like over interpretation. For example, "this class of ARGs is under

anthropogenic pressure" requires more evidence.
We rephrased to “These results could indicate that quinolone, bacitracin and quinolone ARGs are under anthropogenic pressure in coastal environments, and future studies should be carried out to investigate this assumption in greater detail.”

14. Two figures were named after "Figure 6".

Thanks, we fixed the figure numbers on text and figure legends

15. Languages could be largely improved. Some sentences are broken. I suggest the authors read the text again before submission.

We rewrote the text and asked native speaking colleagues to revise this new version of our manuscript.

Here are some minor issues:

16. I suggest the authors include line numbers. Otherwise, it is quite difficult for reviewers to describe the locations of a problem.

We added the line numbers in the new version.

17. Many statements, such as "Antibiotic-resistant bacteria cause over 700,000 deaths per year", should has a reference

We rephrase this sentence and added references where it was pertinent.

18. Gene names should be in lower case and italic. Please pay attention to the uniform layout.

Thanks, we revise the text for that.

19. Some statements are subjectively described and lack of key numbers or statistics.

We revise the manuscript and removed subjective sentences.

20. Colloquial expressions should be avoided. Eg, "very high", "Surprisingly".

We went through the text and deleted or replaced colloquial expressions

21. Texts and legends in figures should be curated to publication level. Eg., pay attention to the italic text, explain all abbreviations, avoid using unclear underlines, etc.

We revised the legends of all figures over the manuscript.

22. Some language revisions are required. Eg., in the abstract, "293 metagenomic" -> "293 metagenomic samples". In "the number of ocean metagenomic projects stored in public databases HAS been growing". However, since the authors did not include line numbering, it is quite difficult to point out the bugs I have located.

We did a language review and fixed the mistakes

23. The abstract is a little bit too long and has presented too much data which is not so critical, which makes the abstract reads like a "Result" section.

We re-wrote the abstract in order to attend the suggestion

24. In the last paragraph of Introduction, "to the 12 oceanic regions co-assembled TARA oceans contigs" sounds unclear to me.

We rephrase this part of the text

25."A total of 124 ARGs were classified as a virus", seriously?

Thanks for pointing this phrasing mistake out - we rephrase this to “classified as potentially located on a viral sequence"

26.FPKM is acceptable for the gene relative abundance, but I suggest the authors to consider using TPM in the later projects.

- We based all our analysis in RPKG instead FPKM (those are just reported as calculated by BBMAP).

rev3

Major comments

The English must me revised. I have made some corrections through the text but I recommend the use of a manuscript editing service.

Thanks, we did an extensive revision on the manuscript. We asked for native speaking colleagues to review the language as well.

One fundamental question that could be addressed with the results is: What is the major factor driving the diversity and abundance of ARGs among the TARA samples? This question remained unanswered despite being one the most relevant questions regarding ARGs. I dont expect the authors to provide a definitive answer for this, but the OLS results should at the very least allow them to speculate on which environmental parameters are mostly related to the diversity and abundance of ARGs in the marine environment,

Of course we can only speculate on this and some hints we already gave in the manuscript but rather short and distributed among the discussion parts (e.g. we discussed the potential effect of nutrient composition at the end of the ARGs quantification on metagenomes chapter. Behind it, very complicated dynamics lie, with 1) abundance of naturally occurring and 2) influence of new ARGs via, e.g., wastewater influx at the beginning and afterwards evolutionary pressure/ecological advantage. We added some parts showing that besides geographical location (tested by tukey HSD tests, the sampling depth and filtration played a big role in many ARG classes.

The authors state that "ORFs of putative ARGs (and its respective contig) were submitted to Kaiju v1.6.2 [26] for taxonomic classification, with the option "run mode" set as "greedy"." There are some issues with this approach. First, classifying a single ORF is different from classifying the whole contig. It is necessary to specify which of the two results was used to classify the ORFs, specially considering that ARGs can be subjected to HGT. Discrepancies between the taxonomic classification of ORF and contig might in fact be suggestive of HGT events. Second, Kaiju is designed for metagenomic reads, when run with contigs it will simply assign taxonomy based on the best match. This is less than ideal. For a robust taxonomic classification of the contigs I recommend the use of an algorithm that establishes a consensus based on the best hits of all proteins in the contigs, such as LCA or CAT/BAT (1)☐.

We agree with the reviewer. Thank you for pointing this out. We decided to remove the contig classification (that was under discussed anyways in the manuscript). For the next version of resistomeDB we will keep the suggestion of using those other algorithms for contig classification.

The manual curation steps need to be described in more detail, specifically, how did you define which CDS needed curation? It sounds unfeasible that that was done for all CDS

We improved the text describing in greater details how the curation was done

The regression analysis is a great approach to identify associations between ARG abundances and enviromental parameters but this analysis has some issues. Were categorical variables (e.g. Marine provinces, Environmental Feature, Ocean sea regions, Fraction and Biogeographic biomes) transformed into numeric variable to be incorporated into the OLS models?

Yes, the categorical variables were converted for dummy variables, this is done automatically by the python package used. The variables were also scaled. (add in the text)

Your data did suggest that Alphaproteobacteria is the major source of marine ARGs. I believe it would be useful to analyse the associations between taxa and ARG classes, this would determine if all taxa harbour the same classes of ARGs or if there are differences between them. This would allow readers to assess which marine taxa are sources of which ARG, and thus determine which represent threats to public health. For simplicity this could be shown in a figure at the level of phylum/class but it would also be useful to have a supplementary table describing each ORF identified as an ARG regarding, assembly source, ARG class and taxonomic affiliation of the ORF and of the full contig.

Testing tax vs. ARGs with the current data would be difficult to execute (many taxas vs. many ARGs) and would lead to very hard interpretation. For example, to find a correlation in this case could indicate only that certain taxa shows certain ARG (what is already shown by the taxonomic classification of the contigs/ARGs), and/or certain taxa produce antibiotic increasing selective pressure. And another possibility would be the that we would see correlation from taxa with high co-occurrence with ARG/antibiotic containing taxa. For proper address the question, methods should be developed using network adjustment approach. We agree that explore those hypothesis would be interesting but this would go beyond the scope of the current manuscript.

The authors rely heavily on the results obtained from PlasFlow. I have concerns about the accuracy of this tool for environmental sequences, specially when dealing with many short contigs analysed by the authors. At the very least I encourage the authors to briefly discuss the error rates of this tool for short sequences and how these potential errors could impact the obtained results.

PlasFlow is a tool developed for screening metagenomic contigs, using a deep learning algorithm, and its accuracy on such data was claimed to be up to 96%, discussed in the original paper published on NAR (https://academic.oup.com/nar/article/46/6/e35/4807335) . However, we are aware of the residual 4 % and we discuss possible limitations as for MCR-1 . We now also added the following part to the plasmid results/discussion chapter: "As in the later chapter on MCR genes, it should be noted here, that PlasFlow analysis bears a small chance (about 4%) to result in false positives as described previously, which especially could be the case with chromosomally integrated plasmids or very short contig sequence sizes."

Minor comments
Title:
Mentioning that you used machine learning tools here is inadequate. Only DeepARG relied on this type of method, and overall your analysis did not rely heavily on machine learning tools besides that. Thus I recommend that the title is changed to: Global ocean resistome revealed: exploring Antibiotic Resistance Genes (ARGs)
abundance and distribution on TARA oceans samples

We changed the title according

In the abstract:

change "In this study, 293 metagenomic from the TARA Oceans" to "In this study, 293 metagenomes from the TARA Oceans"

We changed this for "metagenomic samples"

In the introduction:
change "could lead to the spread of clinical important ARGs across" to "could lead to the spread of clinically important ARGs across"
changed

change "the number of ocean metagenomic projects stored in public databases have been growing," to "the number of ocean metagenomic projects stored in public databases has been growing,"

done

The statement "We also explored the presence of ARGs in mobile genetic elements (MGEs) in TARA samples to investigate the potential of these oceanic environments to act as a reservoir of ARGs." is inadequate as the only MGEs analysed were plasmids and others such as transposons were not. The sentence must be rephrased to reflect that.

We replaced MGEs for plasmids in the text.

In the methods

In the section "A total of 12 co-assembled metagenomes (from different oceanic regions explored by the Tara Oceans expedition), with contigs larger than 1 kilobase were obtained from the dataset published in 2017 by Delmont et al. [22]. Raw reads of 378 shotgun sequencing runs of 243 samples were obtained from the EBI ENA database" there is a discrepancy to what was said in the abstract that mentions 293 metagenomes. The reason for this difference must be clarified.

We explain the difference in the initial number of samples and the number used for quantification/statistic methods when we found that the method for AGS does not perform in virus enriched samples and then we decided to drop those samples from the study.

Change "Note that the abundance was calculated as" to "Note that the abundances were calculated as"

Done

The following section is a bit confusing and needs to be rewritten: "The coverage, in terms of read counts and the abundance of each ARG was then calculated for each sample. Note that the abundance was calculated as Fragments Per Kilobase Million (FPKM). The Average Genome Size (AGS) and Genome Equivalents (GE) were estimated by the software MicrobeCensus v1.0.7 [29] in order to calculate Reads Per Kilo Genome equivalents (RPKG) as described by MicrobeCensus authors [29]. RPKG values for ORFs in each ARG family were summed for each sample."

We re-wrote this part of the text for clarification

Also, some clarifications are necessary: First, the concept of "genome equivalents" must be clarified for those not familiar with MicrobeCensus. Second, It would be useful to have the specific formula used to calculate ARG abundance in the samples. Third, the classification of ARGs into ARG families needs to be clarified as this is a fundamental step of the analysis. Finally, it is unclear how the average genome size - was integrated into the formula to calculate RPKG values.

We added text and formula explaining Genome equivalents and RPKG calculations.
About the ARG family, it was a misuse of the term, as we wanted to refer to the group of sequences classified as ARG by deepARG (for example, MCR-1). We fixed this along the text replacing ARG family by ARG.

In "ARGRPKG ~ Marine provinces + Environmental Feature + Ocean sea regions + Fraction + Biogeographic biomes + Latitude + Longitude + NO2 + PO4 + NO2NO3 + SI + miTAGSILVATaxo Richness + miTAG_SILVA_Phylo_Diversity + miTAG_SILVA_Chao + miTAG_SILVA_ace + miTAG_SILVA_Shannon + OG_Shannon + OG_Richness + OG_Evenness + FC_heterotrophs_cells_mL + FC_autotrophs_cells_mL + FC_bacteria_cells_mL + FC_picoeukaryotes_cells_mL where ARGRPKG (the dependent variable) is the sum of RPKM of all ARGs in a given class and all the dependent variables are the selected environmental features. ANOVA was then conducted on the coefficients obtained from the OLS regression in order to infer the significance of a feature." It appears you used every available feature to train the OLS models. This is problematic because having so many input variables might lead to overfitting. I recommend that before fitting the OLS models the authors use some method for filtering features before including them

Thank you for the suggestion. We checked the model for multiple collinearity and removed variables with high collinearity. Our idea with the model is rather find correlations than to make predictions so we did not did cross-validation to estimate overfit (also because the number of samples is not that high). On the other hand we provide now a number of diagnostic variables from the model, helping to evaluate better the performance.

In the results:
change "families in the databases with low supportive evidence for ARG prediction" to "families in the databases with low support for ARG prediction"

changed

change "A total of 34 ARG families was identified as" to "A total of 34 ARG families were identified as"

done

In "Furthermore, the fosmidomycin and quinolone ARGs were significantly (adjusted p-value 0.0014 and 0.0438, respectively) more abundant in the coastal biome than in the trades biome (Figure 3, Supplementary Table 2)." I could not identify exactly which row of this table refers to this specific test.

This part of the text was rephrased with results from the new model that was slightly different. We also made the tables with the results more clear.

In "These results could indicate that specifically, this class of ARGs is under anthropogenic pressure, and future studies should be carried to investigate it in greater detail." Is this not true for all classes of ARGs?

We rephrase to make the sentence more clear: These results could indicate that specifically, those classes of ARGs are under anthropogenic pressure in the coastal environments, and future studies should be carried to investigate it in greater detail.

Figure 3 and 4: What is the meaning of the negative RPKG values?

The figure shows confidence intervals (CI). The 95% confidence interval is providing a range that you are 95% confident the true difference in means falls in. Thus, the CI can include negative numbers, because the difference in means may be negative. However, now we log-transformed the RPKG values, and then the CI will be the difference of means on the log scale.

Change "For example, for quinolone, the model shows inorganic phosphate $(PO4)3-$ inverted correlated (beta -0.13, p-value 0.0002), NO2NO3 (Nitrite+Nitrate concentration) direct correlated (beta 0.007, p-value 0.002)." to "For example, for quinolone, the model shows inorganic phosphate $(PO4)3-$ negatively correlated (beta -0.13, p-value 0.0002), NO2NO3 (Nitrite+Nitrate concentration) positively correlated (beta 0.007, p-value 0.002)."

We changed over the text inverted for negatively and direct to positively.

I recommend that the authors include more data on the overall performance of the OLS models so that readers can assess how good they are in predicting the abundance of ARGs across samples. Ideally, the results of the OLS analysis should be made available as a supplementary table (My version of the manuscript had no link to table S3).

We uploaded again all the supplementary data, including the table with the model performance.

"This result raises concerns about the impact of antibiotics from the production of pharmaceuticals and wastewater in marine environments." Its unclear to me how you reached this conclusion based on this result alone

We removed the sentence

In "The results suggested that 5 ORFs (from genus Psychrobacter, family Moraxellaceae [65]) are close to the MCR-1/2 clade with support value 1 (Figure 7)." I believe authors meant to reference figure 6 and not 7

Thanks. Indeed it was referring to figure 6 and this was fixed in the text.

Table S2: What is the meaning of the "quantification" column?

The quantification column is a dummy variable showing if the ARG gene was selected for downstream quantification analysis or not based on the manual curation (those in scenario i-v were not used)

Captions for the supplementary tables must be provided to help with their interpretation.

In the conclusions:
"For a limited number of ARGs, gene transcription could be shown." The results did not report on the results of ARG abundances in the TARA metatranscriptomes, hence this statement is inadequate.

Thanks for pointing that out. We removed that phrase.

I disagree with this statement: "Our study also exposes the importance of monitoring coastal water for anthropogenic impact, since the inflow of antibiotics by e.g. wastewater might strengthen (by selective pressure) the antibiotic resistance development by microorganisms.". Although coastal waters can be contaminated with antibiotics, it is unlikely that they reach inhibitory concentrations at these ecosystems. Instead, the real issue is the contamination of these ecosystems with antibiotic resistant bacteria and their ARGs

We agree and now we rewrite the sentences accordingly, highlighting the possibility of HGT from anthropogenic sources to environmental strains.

Your data does not report on ARG among Antartic soils/ice hence the statement "Antarctic soil and ice might be a huge potential reservoir for ARGs, and the discussion about ARG distribution should not neglect the future impact of this reservoir under the influence of climate change." is inadequate for the conclusions

We remove the sentence from our conclusion

In resistomedb.com:

Congratulations on putting this together, it is an excellent resource!

On the "Taxonomic level" section Class should come before Order

Thank you for noting this mistake. We fixed the order of taxonomic level accordingly.

The watercolour map displayed when using the "Explore by antibiotic class" feature is inadequate, the colours of the water and the features are too similar and therefore it is difficult to visualize

Thank you for pointing that. Indeed it was a mistake in the version pushed for production and now it is fixed.

provide a caption for the displayed results of the OLS analysis

We added the caption

References

1. von Meijenfeldt FAB, Arkhipova K, Cambuy DD, Coutinho FH, Dutilh BE. Robust taxonomic classification of uncharted microbial sequences and bins with CAT and BAT. Genome Biol [Internet]. 2019;20(217):530188. Available from: http://biorxiv.org/content/early/2019/01/24/530188.abstract

Close
