# Supplementary material for: Global ocean resistome revealed: Exploring antibiotic resistance gene abundance and distribution in TARA Oceans samples
Source: Gigascience. 2020 May 11;9(5):giaa046. doi: 10.1093/gigascience/giaa046 (PMC7213576; doi:10.1093/gigascience/giaa046)
Supplement: giaa046_Response_to_Reviewer_Comments_Revision_1 [file giaa046_response_to_reviewer_comments_revision_1.pdf]

Author's Response To Reviewer Comments

Close

Dear Editor,
we would like to thank you for the decision letter, and for the opportunity to resubmit a revised copy of the manuscript.
We would also like to take this opportunity to express our thanks to the reviewers for the positive feedback and helpful comments for corrections and manuscript improvements.
Please find below the point by point reply to reviewers comments.

Kind regards,

Related to the Previous Q1:
I can now access the server. But there seem some bugs, or things could be improved, eg.:
1) In the sequence viewer, when I want to check the sequence alignment and drag the position, it always returns to position 0;

Thanks for the comment, we noticed that in some browsers/versions this issue is happening. We are using a third-party tool for displaying the alignment and we are in touch with the developers to find a solution for this.

2) As a database, I saw a lot of data tables on the server. I wonder if users could download these tables as .csv or .tsv files;

The data tables used in the resistomedb.com app can be downloaded at the GitHub project page (https://github.com/rcuadrat/resistome_dash) under the folder data. We included the link for the data in the app. We add the link also in the manuscript "Dash web application for data exploration and visualisation section"

3) Also, it could be better if the data forms supporting the figures could be provided for users for personalized statistics or visualization;

We included the data in the GitHub of the main pipeline (https://github.com/rcuadrat/ocean_resistome).

3) The tables for genes annotated from contigs may miss some key information, such as alignment identity, alignment length, etc.

We did not include all the info due to lack of space for visualization. However, the full table can be downloaded from the data folder in the same GitHub repository mentioned in answers 2 (and now with the link in the app).

Related to the Previous Q2:
Regarding "a manual curation of each ARG to check for misannotations and inconsistencies" on alignment, P4 Line, the revised manuscript has largely improved, as standards now summarized into bullet points. However, I still feel a necessity to include clear details to dispel misunderstanding possibilities, for example: (i) "Sequences exhibiting non-ARGs as top 5 BLASTp hits", is it via blasting against NR database? Or other alignments? Is this the only criterium for point (i)?

Yes, blasting was against NR database and hits only were considered after reaching an e-value cutoff of e-5. We included these necessary details in the text.

(ii) "housekeeping genes that confer resistance only when

specifically mutated", is there a clear list for these genes?
This list is compiled as supplementary table 2 (ten genes which belong tocategory ii). The information was manually compiled from the literature and CARD database.
Same issue for (iii) and (iv).
Also this information can be found in the supplementary table 2 (9 genes for category iii and 41 for category iv).

(v) please define "both similarities to ARGs and non-ARGs", by identity, e-value or what else?

We specified this to a lower extent already in the following line in the manuscript ("BLASTp and CDD analyses were used to classify ARGs into category (v) in cases where the TARA sequences show, non-ARGs and no specific CDD domain for that ARG among the top 10 blastp hits").
We did not use specific cutoffs (of course only the most recommended e-5 used in general for blast searches). This was a manual curation with the individual decision taken non-automatically by the curator. For most of the cases, we hypothesize that using phylogenetic approaches would be more suitable for classifying those genes, so we keep them for future works.

Also, I disagree with the use of "top n blastp hits", it is not statistical. Different genes may have different numbers of homologous genes from different species, which makes this criterium unfair for all candidate genes.

We agree with the reviewer, this is why we chose a manual curation based on expert review and database searches. Each gene was analysed individually by a curator (the authors), based on best database hits, but also taking similarity, alignment coverage, e-value and literature research. For example, a potential ARG was not removed when the top 5 hits revealed 4 ARGs with good alignment stats, but with a 5th non-ARG showing a worse alignment. Since such a manual curation is limited several cutoffs were used to limit the amount of genes to analyze manually . Our ongoing work involves developing a full database paper, in which the community is invited to help curating these and more potential ARGs from further databases in the future.

Related to the Previous Q3:
I suggest the authors quote some references to say, "these data are clean data", or put some basic QC statistics.

For fastq submissions to EBI, it is mandatory to remove artificial sequences (primer, adaptors) as stated in https://ena-docs.readthedocs.io/en/latest/submit/fileprep/reads.html. This is the most crucial step on QC of Illumina reads, as the bbmap and other aligners are not too much sensitive to low-quality bases when doing mapping for quantification (biorxiv.org/content/10.1101/833962v1). In this study, we did not use the raw reads for assembly, but rather mapped reads to the previous published assembled dataset, so we found no necessity to show QC of the reads that passed the EBI check control.

Related to the Previous Q7c:
Many parameters are now available, especially for the Methods section "Environmental ARG prediction". But I am not sure regarding ARG annotation, "BLASTp with default parameter" should be accepted. Normally ARGs should have an Identity >70, >90 or even >95, depending on the sample types and clinical relevance. To my point of view, identity (or also E-value) should be used for a basic filtration.

We added the general cutoff we used to accept a blast hit (e-5). Identity cutoffs are hard to implement due to the different evolutionary rates of the very different gene families analysed.

Parameter missing problem still partly remains in the Methods section "Phylogenetic analysis of environmental ARGs" and "ARGs quantification and statistical tests on metagenomic samples".

We added the statement ("default parameters") for the tools used because we did not modify any parameters. Together with the given version this should be consistent and make replication possible. The used phylogenetic tools are standard in the analysis pipeline Phylogenetic.fr. For the bbmap and microbecensus, we also added the "default parameters" in the text, and we reinforce the fact that the scripts used for those analyses are available on the GitHub repository, making it fully reproducible.

Related to Previous Q10:
Statistics is better than the description in the text. Also, regarding Table 1, I am curious how many of these contig-sharing ARGs are the same ARG, or from the same ARG family. If the authors could annotate the phage-like sequences or mobile elements, the story could be improved.

From the 4192 ones with 2 ARGs, only 74 shows the same annotation for both ARGs (33 classified as a plasmid). We added this information in the text.

Related to Previous Q18:
As I can see, gene names in the Figures remain unchanged. Eg. should be tetB, ermB, not TETB, ERMB.

Thank you for pointing this out. We fixed the figures with the right gene notations.

Rafael Cuadrat and Alberto Davila

Close
