# Supplementary material for: Global ocean resistome revealed: Exploring antibiotic resistance gene abundance and distribution in TARA Oceans samples
Source: Gigascience. 2020 May 11;9(5):giaa046. doi: 10.1093/gigascience/giaa046 (PMC7213576; doi:10.1093/gigascience/giaa046)
Supplement: giaa046_Reviewer_1_Report_Original_Submission — Carlos Loucera MuÃ±ecas -- 1/28/2020 Reviewed [file giaa046_reviewer_1_report_original_submission.pdf]

Reviewer Report

Title: Global ocean resistome revealed: exploring Antibiotic Resistance Genes (ARGs) abundance and distribution on TARA oceans samples

Version: Original Submission **Date:** 1/28/2020

Reviewer name: Carlos Loucera MuÃ±ecas

Reviewer Comments to Author:

Summary
The paper proposes an interesting web application for exploring the antibiotic resistance profiles across the oceans by means of a combination of different tools and open resources. The lack of metagenomics resources compared to other biology fields makes the interpretation of research results a very difficult task. The addition of curated databases, more so if it is supported with a well researched and reproducible pipeline (as the one being reviewed), are key to accelerate the progress in the field.
I appreciate the concise writing and the code, resources availability, and pipeline reproducibility.
However, I have some concerns:
Minor
- [Abstract] ORFs abbreviation used before being defined
- [Introduction] The statement "Antibiotic-resistant bacteria cause over 700,000 deaths per year,public health issue and an economic burden to the entire world" needs a reference.
- [General] Given that deepArg is such a novel method it should be briefly explained. This would lead to a more readable paper for the non ML-specialist reader, furthermore it would make the title more consistent with the contents.
- [Figure 6] Barely readable, the figure needs fixing by using a vectorized version or a higher DPI version.
- [Material and Methods] To perform OLS regression by adding the RPKM of all ARGs (ARG_{RPKG}) sound a bit strange to me. A multi-input multi-output abalysis shloud be possible using some sort of PLS-regression technique.
- [Results and Discussion] "For plasmid classification, we relied on the results of PlasFlow, which only classified two of the sequences classified located on a plasmid. This can be explained by the". Sounds award to me due to word repetition,
- [Conclusions] " conferring probably resistance to 26 classes of antibiotics. " Sounds award to me.
Major: (Very personal)
- In my humble opinion the title seems to suggest that Machine Learning plays a key role in the discussion of the paper. However, the paper uses ml tools as black boxes without any kind of explanation or comparison with other methods. This can be solved by briefly explaining what the deepArg NN does and comparing the results with other ARG prediction models.

Methods

Are the methods appropriate to the aims of the study, are they well described, and are necessary controls included? Choose an item.

Conclusions

Are the conclusions adequately supported by the data shown? Choose an item.

Reporting Standards

Does the manuscript adhere to the journal's guidelines on minimum standards of reporting? Choose an item.

Choose an item.

Statistics

Are you able to assess all statistics in the manuscript, including the appropriateness of statistical tests used? Choose an item.

Quality of Written English

Please indicate the quality of language in the manuscript: Choose an item.

Declaration of Competing Interests

Please complete a declaration of competing interests, considering the following questions:

- Have you in the past five years received reimbursements, fees, funding, or salary from an organisation that may in any way gain or lose financially from the publication of this manuscript, either now or in the future?
- Do you hold any stocks or shares in an organisation that may in any way gain or lose financially from the publication of this manuscript, either now or in the future?
- Do you hold or are you currently applying for any patents relating to the content of the manuscript?
- Have you received reimbursements, fees, funding, or salary from an organization that holds or has applied for patents relating to the content of the manuscript?
- Do you have any other financial competing interests?
- Do you have any non-financial competing interests in relation to this paper?

If you can answer no to all of the above, write 'I declare that I have no competing interests' below. If your reply is yes to any, please give details below.

I declare that I have no competing interests.

I agree to the open peer review policy of the journal. I understand that my name will be included on my report to the authors and, if the manuscript is accepted for publication, my named report including any attachments I upload will be posted on the website along with the authors' responses. I agree for my

report to be made available under an Open Access Creative Commons CC-BY license (http://creativecommons.org/licenses/by/4.0/). I understand that any comments which I do not wish to be included in my named report can be included as confidential comments to the editors, which will not be published.

Choose an item.

To further support our reviewers, we have joined with Publons, where you can gain additional credit to further highlight your hard work (see: https://publons.com/journal/530/gigascience). On publication of this paper, your review will be automatically added to Publons, you can then choose whether or not to claim your Publons credit. I understand this statement.

Yes Choose an item.
