# Supplementary material for: Global ocean resistome revealed: Exploring antibiotic resistance gene abundance and distribution in TARA Oceans samples
Source: Gigascience. 2020 May 11;9(5):giaa046. doi: 10.1093/gigascience/giaa046 (PMC7213576; doi:10.1093/gigascience/giaa046)
Supplement: giaa046_Reviewer_2_Report_Original_Submission — Kang Kang -- 2/13/2020 Reviewed [file giaa046_reviewer_2_report_original_submission.pdf]

Reviewer Report

Title: Global ocean resistome revealed: exploring Antibiotic Resistance Genes (ARGs) abundance and distribution on TARA oceans samples

Version: Original Submission **Date:** 2/13/2020

Reviewer name: Kang Kang

Reviewer Comments to Author:

The authors applied a deep learning method to screen ARGs in the TARA ocean dataset, performed phylogeny analysis, and ARG abundance analysis. A regression model was constructed to associate the ARG profiles and ocean metagenomic sample metadata. Also, a database was constructed. I highly appreciated the comprehensive tasks finished by the authors, which could serve as a valuable toolkit and fundamental statistics for the future ocean microbiome and resistome studies.

However, in my opinion, the current manuscript is still unmatured, in particular in the method description and the logic flow in the result section. Several method sections were not described in sufficient detail, and some details are also arguable. The purposes of many analyses are not clearly described, which makes the manuscript also a bit difficult to follow.

Here are some major concerns:

1. The website, http://resistomedb.com, seems not accessible (I just saw "Loading..." all the time).
2. In the flowchart, "manual curation" only happens before the phylogeny analysis, I wonder if this is correct.
3. In the flowchart, Tara raw reads (fastq) were used. However, I did not see a Data QC process.
4. Many, many sections in the "Results" are describing methods (eg. manual curation), which should be relocated to "Methods". This also makes the readers quite difficult to follow the manuscript.
5. I hope the authors could show a clearer storyline in the Result sections. For what purposes those analyses were done
6. Figures and their legends should be self-explanatory. Eg., the ranges for each data point in Figure 3 were not explained (CI? SD?). How the "reference" is chosen is also not explained, and why sometimes the lower one was selected as the reference and in some other cases, the one with higher relative abundance was the reference.
7. The method sections are not detailed enough for reproduction. For example:

a. I did not capture the detailed filtration criteria for the ARG ORF prediction. Short ORF fragments may influence the downstream analysis (including the multi-ARG presence on one contig analysis).

b. "a manual curation of each ARG" was not sufficiently described. It is also not clear if "Online BLASTp searches" and "Conserved domains (CDDs) and annotations in the source databases (ARDB, CARD and UniProt)" are associated with the manual curation. Also, the authors did not describe how these alignment results were used.

c. Some software parameters are. Eg., parameters, especially "threshold", for PlasFlow were not mentioned.

d. There should be a section for the summary for all statistical tests. Also, p-values were adjusted but

the method was not mentioned.
8. FPKM stands for "Fragments Per Kilobase per Million mapped reads". The authors made it wrong as "Fragments Per Kilobase Million". Thus, I worried if the basic data processing was credible.
9. In the ARGRPKG model, statistical tests were mentioned but no significance cutoff was given. Variable selection needs to be addressed. I am not sure if all variables are independent. If not, the model could be problematic. Also, I am curious if the authors have tried to reduce the numbers of variables and evaluate the over-fitting.
10. Regarding Table 1, the authors declared that some contigs with 2 or more ARGs were annotated. However, this might because two ARG fragments from the same ARG were annotated on both edges of a circular contig.
11. The authors should be very careful when describing "prevalence", "abundance" and "relative abundance". "abundance" was highly used in the manuscript; however, they should be relative abundance. And in Figure 2, the highest number of ORFs does not support "most abundant".
12. Tukey HSD tests were widely used in this study, and this is a test for parametric data to compare mean values. From Figure 5, I guess the RPKG relative abundance data should be quite nonparametric. I doubt this test could be applied for such data, as no data normalization was mentioned.
13. Some conclusion seems like over interpretation. For example, "this class of ARGs is under anthropogenic pressure" requires more evidence.
14. Two figures were named after "Figure 6".
15. Languages could be largely improved. Some sentences are broken. I suggest the authors read the text again before submission.
Here are some minor issues:
16. I suggest the authors include line numbers. Otherwise, it is quite difficult for reviewers to describe the locations of a problem.
17. Many statements, such as "Antibiotic-resistant bacteria cause over 700,000 deaths per year", should has a reference
18. Gene names should be in lower case and italic. Please pay attention to the uniform layout.
19. Some statements are subjectively described and lack of key numbers or statistics.
20. Colloquial expressions should be avoided. Eg, "very high", "Surprisingly".
21. Texts and legends in figures should be curated to publication level. Eg., pay attention to the italic text, explain all abbreviations, avoid using unclear underlines, etc.
22. Some language revisions are required. Eg., in the abstract, "293 metagenomic" -> "293 metagenomic samples". In "the number of ocean metagenomic projects stored in public databases HAS been growing". However, since the authors did not include line numbering, it is quite difficult to point out the bugs I have located.
23. The abstract is a little bit too long and has presented too much data which is not so critical, which makes the abstract reads like a "Result" section.
24. In the last paragraph of Introduction, "to the 12 oceanic regions co-assembled TARA oceans contigs" sounds unclear to me.
25."A total of 124 ARGs were classified as a virus", seriously?
26.FPKM is acceptable for the gene relative abundance, but I suggest the authors to consider using TPM

in the later projects.

Methods

Are the methods appropriate to the aims of the study, are they well described, and are necessary controls included? Choose an item.

Conclusions

Are the conclusions adequately supported by the data shown? Choose an item.

Reporting Standards

Does the manuscript adhere to the journal's guidelines on minimum standards of reporting? Choose an item.

Choose an item.

Statistics

Are you able to assess all statistics in the manuscript, including the appropriateness of statistical tests used? Choose an item.

Quality of Written English

Please indicate the quality of language in the manuscript: Choose an item.

Declaration of Competing Interests

Please complete a declaration of competing interests, considering the following questions:

- Have you in the past five years received reimbursements, fees, funding, or salary from an organisation that may in any way gain or lose financially from the publication of this manuscript, either now or in the future?
- Do you hold any stocks or shares in an organisation that may in any way gain or lose financially from the publication of this manuscript, either now or in the future?
- Do you hold or are you currently applying for any patents relating to the content of the manuscript?
- Have you received reimbursements, fees, funding, or salary from an organization that holds or has applied for patents relating to the content of the manuscript?
- Do you have any other financial competing interests?
- Do you have any non-financial competing interests in relation to this paper?

If you can answer no to all of the above, write 'I declare that I have no competing interests' below. If your reply is yes to any, please give details below.

I declare that I have no competing interests.

I agree to the open peer review policy of the journal. I understand that my name will be included on my report to the authors and, if the manuscript is accepted for publication, my named report including any attachments I upload will be posted on the website along with the authors' responses. I agree for my report to be made available under an Open Access Creative Commons CC-BY license (http://creativecommons.org/licenses/by/4.0/). I understand that any comments which I do not wish to be included in my named report can be included as confidential comments to the editors, which will not be published.

Choose an item.

To further support our reviewers, we have joined with Publons, where you can gain additional credit to further highlight your hard work (see: https://publons.com/journal/530/gigascience). On publication of this paper, your review will be automatically added to Publons, you can then choose whether or not to claim your Publons credit. I understand this statement.

Yes Choose an item.
