# Supplementary material for: Global ocean resistome revealed: Exploring antibiotic resistance gene abundance and distribution in TARA Oceans samples
Source: Gigascience. 2020 May 11;9(5):giaa046. doi: 10.1093/gigascience/giaa046 (PMC7213576; doi:10.1093/gigascience/giaa046)
Supplement: giaa046_Reviewer_2_Report_Revision_1 — Kang Kang -- 3/27/2020 Reviewed [file giaa046_reviewer_2_report_revision_1.pdf]

Reviewer Report

Title: Global ocean resistome revealed: exploring Antibiotic Resistance Genes (ARGs) abundance and distribution on TARA oceans samples

Version: Revision 1 **Date:** 3/27/2020

Reviewer name: Kang Kang

Reviewer Comments to Author:

Thanks for the hard work from the authors. The manuscript has been improved regarding the storyline, language, and details. Here are some comments on the authors' responses, or some issues remaining unclear to me. I hope the authors could address these questions and furtherly improve the manuscript.
Related to the Previous Q1:
I can now access the server. But there seem some bugs, or things could be improved, eg.:
1) In the sequence viewer, when I want to check the sequence alignment and drag the position, it always returns to position 0;
2) As a database, I saw a lot of data tables on the server. I wonder if users could download these tables as .csv or .tsv files;
3) Also, it could be better if the data forms supporting the figures could be provided for users for personalized statistics or visualization;
3) The tables for genes annotated from contigs may miss some key information, such as alignment identity, alignment length, etc.
Related to the Previous Q2:
Regarding "a manual curation of each ARG to check for misannotations and inconsistencies" on alignment, P4 Line, the revised manuscript has largely improved, as standards now summarized into bullet points. However, I still feel a necessity to include clear details to dispel misunderstanding possibilities, for example: (i) "Sequences exhibiting non-ARGs as top 5 BLASTp hits", is it via blasting against NR database? Or other alignments? Is this the only criterium for point (i)? (ii) "housekeeping genes that confer resistance only when
specifically mutated", is there a clear list for these genes? Same issue for (iii) and (iv). (v) please define "both similarities to ARGs and non-ARGs", by identity, e-value or what else? Also, I disagree with the use of "top n blastp hits", it is not statistical. Different genes may have different numbers of homologous genes from different species, which makes this criterium unfair for all candidate genes.
Related to the Previous Q3:
I suggest the authors quote some references to say, "these data are clean data", or put some basic QC statistics.
Related to the Previous Q7c:
Many parameters are now available, especially for the Methods section "Environmental ARG prediction". But I am not sure regarding ARG annotation, "BLASTp with default parameter" should be accepted. Normally ARGs should have an Identity >70, >90 or even >95, depending on the sample types and clinical relevance. To my point of view, identity (or also E-value) should be used for a basic filtration.

Parameter missing problem still partly remains in the Methods section "Phylogenetic analysis of environmental ARGs" and "ARGs quantification and statistical tests on metagenomic samples".
Related to Previous Q10:
Statistics is better than the description in the text. Also, regarding Table 1, I am curious how many of these contig-sharing ARGs are the same ARG, or from the same ARG family. If the authors could annotate the phage-like sequences or mobile elements, the story could be improved.
Related to Previous Q18:
As I can see, gene names in the Figures remain unchanged. Eg. should be tetB, ermB, not TETB, ERMB.

Methods

Are the methods appropriate to the aims of the study, are they well described, and are necessary controls included? Choose an item.

Conclusions

Are the conclusions adequately supported by the data shown? Choose an item.

Reporting Standards

Does the manuscript adhere to the journal's guidelines on minimum standards of reporting? Choose an item.

Choose an item.

Statistics

Are you able to assess all statistics in the manuscript, including the appropriateness of statistical tests used? Choose an item.

Quality of Written English

Please indicate the quality of language in the manuscript: Choose an item.

Declaration of Competing Interests

Please complete a declaration of competing interests, considering the following questions:

- Have you in the past five years received reimbursements, fees, funding, or salary from an organisation that may in any way gain or lose financially from the publication of this manuscript, either now or in the future?
- Do you hold any stocks or shares in an organisation that may in any way gain or lose financially from the publication of this manuscript, either now or in the future?

- Do you hold or are you currently applying for any patents relating to the content of the manuscript?
- Have you received reimbursements, fees, funding, or salary from an organization that holds or has applied for patents relating to the content of the manuscript?
- Do you have any other financial competing interests?
- Do you have any non-financial competing interests in relation to this paper?

If you can answer no to all of the above, write 'I declare that I have no competing interests' below. If your reply is yes to any, please give details below.

I declare that I have no competing interests

I agree to the open peer review policy of the journal. I understand that my name will be included on my report to the authors and, if the manuscript is accepted for publication, my named report including any attachments I upload will be posted on the website along with the authors' responses. I agree for my report to be made available under an Open Access Creative Commons CC-BY license (http://creativecommons.org/licenses/by/4.0/). I understand that any comments which I do not wish to be included in my named report can be included as confidential comments to the editors, which will not be published.

Choose an item.

To further support our reviewers, we have joined with Publons, where you can gain additional credit to further highlight your hard work (see: https://publons.com/journal/530/gigascience). On publication of this paper, your review will be automatically added to Publons, you can then choose whether or not to claim your Publons credit. I understand this statement.

Yes Choose an item.
