# Supplementary material for: Global ocean resistome revealed: Exploring antibiotic resistance gene abundance and distribution in TARA Oceans samples
Source: Gigascience. 2020 May 11;9(5):giaa046. doi: 10.1093/gigascience/giaa046 (PMC7213576; doi:10.1093/gigascience/giaa046)
Supplement: giaa046_Reviewer_3_Report_Original_Submission — Felipe Hernandes -- 2/17/2020 Reviewed [file giaa046_reviewer_3_report_original_submission.pdf]

Reviewer Report

Title: Global ocean resistome revealed: exploring Antibiotic Resistance Genes (ARGs) abundance and distribution on TARA oceans samples

Version: Original Submission **Date:** 2/17/2020

Reviewer name: Felipe Hernandes

Reviewer Comments to Author:

In this manuscript the authors make use of the TARA Oceans dataset to determine global marine resistome. The topic of this manuscript if of utmost relevance as bacterial resistance to antibiotics represents a major public health issue. The results provided by the authors and the companion website are rich resources for understanding the emergence and spread of antibiotic resistance genes in natural habitats. The paper is well written, the conclusions are supported by the data and the statistical analysis is adequate, albeit needing some improvements and clarifications. Hence, this is a manuscript that deserves to be published. Nevertheless, there are some major issues that must be addressed to make it suitable for publication.
Major comments
The English must me revised. I have made some corrections through the text but I recommend the use of a manuscript editing service.
One fundamental question that could be addressed with the results is: What is the major factor driving the diversity and abundance of ARGs among the TARA samples? This question remained unanswered despite being one the most relevant questions regarding ARGs. I dont expect the authors to provide a definitive answer for this, but the OLS results should at the very least allow them to speculate on which environmental parameters are mostly related to the diversity and abundance of ARGs in the marine environment,
The authors state that "ORFs of putative ARGs (and its respective contig) were
submitted to Kaiju v1.6.2 [26] for taxonomic classification, with the option "run mode"
set as "greedy"." There are some issues with this approach. First, classifying a single ORF is different from classifying the whole contig. It is necessary to specify which of the two results was used to classify the ORFs, specially considering that ARGs can be subjected to HGT. Discrepancies between the taxonomic classification of ORF and contig might in fact be suggestive of HGT events. Second, Kaiju is designed for metagenomic reads, when run with contigs it will simply assign taxonomy based on the best match. This is less than ideal. For a robust taxonomic classification of the contigs I recommend the use of an algorithm that establishes a consensus based on the best hits of all proteins in the contigs, such as LCA or CAT/BAT (1)â .
The manual curation steps need to be described in more detail, specifically, how did you define which CDS needed curation? It sounds unfeasible that that was done for all CDS
The regression analysis is a great approach to identify associations between ARG abundances and enviromental parameters but this analysis has some issues. Were categorical variables (e.g. Marine provinces, Environmental Feature, Ocean sea regions, Fraction and Biogeographic biomes) transformed

into numeric variable to be incorporated into the OLS models?
Your data did suggest that Alphaproteobacteria is the major source of marine ARGs. I believe it would be useful to analyse the associations between taxa and ARG classes, this would determine if all taxa harbour the same classes of ARGs or if there are differences between them. This would allow readers to assess which marine taxa are sources of which ARG, and thus determine which represent threats to public health. For simplicity this could be shown in a figure at the level of phylum/class but it would also be useful to have a supplementary table describing each ORF identified as an ARG regarding, assembly source, ARG class and taxonomic affiliation of the ORF and of the full contig.
The authors rely heavily on the results obtained from PlasFlow. I have concerns about the accuracy of this tool for environmental sequences, specially when dealing with many short contigs analysed by the authors. At the very least I encourage the authors to briefly discuss the error rates of this tool for short sequences and how these potential errors could impact the obtained results.
Minor comments
Title:
Mentioning that you used machine learning tools here is inadequate. Only DeepARG relied on this type of method, and overall your analysis did not rely heavily on machine learning tools besides that. Thus I recommend that the title is changed to: Global ocean resistome revealed: exploring Antibiotic Resistance Genes (ARGs)
abundance and distribution on TARA oceans samples
In the abstract:
change "In this study, 293 metagenomic from the TARA Oceans" to "In this study, 293 metagenomes from the TARA Oceans"
In the introduction:
change "could lead to the spread of clinical important ARGs across" to "could lead to the spread of clinically important ARGs across"
change "the number of ocean metagenomic projects stored in public databases have
been growing," to "the number of ocean metagenomic projects stored in public databases has
been growing,"
The statement "We also explored the presence of ARGs in mobile genetic elements (MGEs) in TARA samples to investigate the potential of these oceanic environments to act as a reservoir of ARGs." is inadequate as the only MGEs analysed were plasmids and others such as transposons were not. The sentence must be rephrased to reflect that.
In the methods
In the section "A total of 12 co-assembled metagenomes (from different oceanic regions explored by the Tara Oceans expedition), with contigs larger than 1 kilobase were obtained from
the dataset published in 2017 by Delmont et al. [22]. Raw reads of 378 shotgun
sequencing runs of 243 samples were obtained from the EBI ENA database" there is a discrepancy to what was said in the abstract that mentions 293 metagenomes. The reason for this difference must be clarified.
Change "Note that the abundance was calculated as" to "Note that the abundances were calculated as"
The following section is a bit confusing and needs to be rewritten: "The coverage,
in terms of read counts and the abundance of each ARG was then calculated for each

sample. Note that the abundance was calculated as Fragments Per Kilobase Million
(FPKM). The Average Genome Size (AGS) and Genome Equivalents (GE) were
estimated by the software MicrobeCensus v1.0.7 [29] in order to calculate Reads Per
Kilo Genome equivalents (RPKG) as described by MicrobeCensus authors [29].
RPKG values for ORFs in each ARG family were summed for each sample."
Also, some clarifications are necessary: First, the concept of "genome equivalents" must be clarified for those not familiar with MicrobeCensus. Second, It would be useful to have the specific formula used to calculate ARG abundance in the samples. Third, the classification of ARGs into ARG families needs to be clarified as this is a fundamental step of the analysis. Finally, it is unclear how the average genome size was integrated into the formula to calculate RPKG values.
In "ARGRPKG ~ Marine provinces + Environmental Feature + Ocean sea regions + Fraction
+ Biogeographic biomes + Latitude + Longitude + NO2 + PO4 + NO2NO3 + SI +
miTAGSILVATaxo Richness + miTAG_SILVA_Phylo_Diversity +
miTAG_SILVA_Chao + miTAG_SILVA_ace + miTAG_SILVA_Shannon +
OG_Shannon + OG_Richness + OG_Evenness + FC_heterotrophs_cells_mL +
FC_autotrophs_cells_mL + FC_bacteria_cells_mL + FC_picoeukaryotes_cells_mL
where ARGRPKG (the dependent variable) is the sum of RPKM of all ARGs in a given
class and all the dependent variables are the selected environmental features. ANOVA
was then conducted on the coefficients obtained from the OLS regression in order to
infer the significance of a feature." It appears you used every available feature to train the OLS models. This is problematic because having so many input variables might lead to overfitting. I recommend that before fitting the OLS models the authors use some method for filtering features before including them
In the results:
change "families in the databases with low supportive evidence for ARG prediction" to "families in the databases with low support for ARG prediction"
change "A total of 34 ARG families was identified as" to "A total of 34 ARG families were identified as"
In "Furthermore, the fosmidomycin and quinolone ARGs were significantly
(adjusted p-value 0.0014 and 0.0438, respectively) more abundant in the coastal
biome than in the trades biome (Figure 3, Supplementary Table 2)." I could not identify exactly which row of this table refers to this specific test.
In "These results could indicate that
specifically, this class of ARGs is under anthropogenic pressure, and future studies
should be carried to investigate it in greater detail." Is this not true for all classes of ARGs?
Figure 3 and 4: What is the meaning of the negative RPKG values?
Change "For
example, for quinolone, the model shows inorganic phosphate (PO4)3âˆ’ inverted
correlated (beta -0.13, p-value 0.0002), NO2NO3 (Nitrite+Nitrate concentration) direct
correlated (beta 0.007, p-value 0.002)." to "For
example, for quinolone, the model shows inorganic phosphate (PO4)3âˆ’ negatively
correlated (beta -0.13, p-value 0.0002), NO2NO3 (Nitrite+Nitrate concentration) positively
correlated (beta 0.007, p-value 0.002)."
I recommend that the authors include more data on the overall performance of the OLS models so that

readers can assess how good they are in predicting the abundance of ARGs across samples. Ideally, the results of the OLS analysis should be made available as a supplementary table (My version of the manuscript had no link to table S3).
"This result raises concerns about the impact of antibiotics from the production of pharmaceuticals and wastewater in marine environments." Its unclear to me how you reached this conclusion based on this result alone
In "The results suggested that 5 ORFs (from genus
Psychrobacter, family Moraxellaceae [65]) are close to the MCR-1/2 clade with support
value 1 (Figure 7)." I believe authors meant to reference figure 6 and not 7
Table S2: What is the meaning of the "quantification" column?
Captions for the supplementary tables must be provided to help with their interpretation.
In the conclusions:
"For a limited
number of ARGs, gene transcription could be shown." The results did not report on the results of ARG abundances in the TARA metatranscriptomes, hence this statement is inadequate.
I disagree with this statement: "Our study also exposes the
importance of monitoring coastal water for anthropogenic impact, since the inflow of
antibiotics by e.g. wastewater might strengthen (by selective pressure) the antibiotic
resistance development by microorganisms.". Although coastal waters can be contaminated with antibiotics, it is unlikely that they reach inhibitory concentrations at these ecosystems. Instead, the real issue is the contamination of these ecosystems with antibiotic resistant bacteria and their ARGs
Your data does not report on ARG among Antartic soils/ice hence the statement "Antarctic soil and ice might be a huge
potential reservoir for ARGs, and the discussion about ARG distribution should not
neglect the future impact of this reservoir under the influence of climate change." is inadequate for the conclusions
In resistomedb.com:
Congratulations on putting this together, it is an excellent resource!
On the "Taxonomic level" section Class should come before Order
The watercolour map displayed when using the "Explore by antibiotic class" feature is inadequate, the colours of the water and the features are too similar and therefore it is difficult to visualize
provide a caption for the displayed results of the OLS analysis
References
1. von Meijenfeldt FAB, Arkhipova K, Cambuy DD, Coutinho FH, Dutilh BE. Robust taxonomic classification of uncharted microbial sequences and bins with CAT and BAT. Genome Biol [Internet]. 2019;20(217):530188. Available from: http://biorxiv.org/content/early/2019/01/24/530188.abstract

Methods

Are the methods appropriate to the aims of the study, are they well described, and are necessary controls included? Choose an item.

Conclusions

Are the conclusions adequately supported by the data shown? Choose an item.

Reporting Standards

Does the manuscript adhere to the journal's guidelines on minimum standards of reporting? Choose an item.

Choose an item.

Statistics

Are you able to assess all statistics in the manuscript, including the appropriateness of statistical tests used? Choose an item.

Quality of Written English

Please indicate the quality of language in the manuscript: Choose an item.

Declaration of Competing Interests

Please complete a declaration of competing interests, considering the following questions:

- Have you in the past five years received reimbursements, fees, funding, or salary from an organisation that may in any way gain or lose financially from the publication of this manuscript, either now or in the future?
- Do you hold any stocks or shares in an organisation that may in any way gain or lose financially from the publication of this manuscript, either now or in the future?
- Do you hold or are you currently applying for any patents relating to the content of the manuscript?
- Have you received reimbursements, fees, funding, or salary from an organization that holds or has applied for patents relating to the content of the manuscript?
- Do you have any other financial competing interests?
- Do you have any non-financial competing interests in relation to this paper?

If you can answer no to all of the above, write 'I declare that I have no competing interests' below. If your reply is yes to any, please give details below.

I declare that I have no competing interests

I agree to the open peer review policy of the journal. I understand that my name will be included on my report to the authors and, if the manuscript is accepted for publication, my named report including any attachments I upload will be posted on the website along with the authors' responses. I agree for my

report to be made available under an Open Access Creative Commons CC-BY license (http://creativecommons.org/licenses/by/4.0/). I understand that any comments which I do not wish to be included in my named report can be included as confidential comments to the editors, which will not be published.

Choose an item.

To further support our reviewers, we have joined with Publons, where you can gain additional credit to further highlight your hard work (see: https://publons.com/journal/530/gigascience). On publication of this paper, your review will be automatically added to Publons, you can then choose whether or not to claim your Publons credit. I understand this statement.

Yes Choose an item.
